# The Allosteric Regulator Inositol Phosphate Dramatically Affects the Efficacy and Selectivity of Inhibitors for Different HDAC Complexes

**DOI:** 10.1021/jacs.5c08929

**Published:** 2025-09-25

**Authors:** Wiktoria A. Pytel, Urvashi Patel, Joshua P. Smalley, Christopher J. Millard, Edward A. Brown, Aline R. Pavan, Siyu Wang, Jay H. Kalin, Jean Leandro dos Santos, Philip A. Cole, James T. Hodgkinson, John W. R. Schwabe

**Affiliations:** † Institute for Structural and Chemical Biology, 4488 University of Leicester, Leicester LE1 7RH, U.K.; ‡ Department of Molecular and Cell Biology, 4488 University of Leicester, Leicester LE1 7RH, U.K.; § School of Chemistry, 4488 University of Leicester, Leicester LE1 7RH, U.K.; ∥ 28108 São Paulo State University (UNESP), School of Pharmaceutical Sciences, 14800-903 Araraquara, Brazil; ⊥ Division of Genetics, Department of Medicine, Brigham and Women’s Hospital and Department of Biological Chemistry and Molecular Pharmacology, 1811 Harvard Medical School, Boston, Massachusetts 02115, United States

## Abstract

Class I histone deacetylases regulate gene transcription and are established therapeutic targets. HDAC1–3 form the catalytic subunit in several distinct multiprotein complexes; however, HDAC inhibitors are rarely studied in the context of these complexes. We evaluated multiple inhibitors, using seven HDAC complexes, and found that the inhibition profiles were highly complex-dependent, despite targeting the same enzyme. We also investigated the effect of the allosteric regulator inositol phosphate on these inhibitors. We observed very large, complex-selective reductions in the potency of benzamides bearing a “foot-pocket group”, proposed to be selective for HDAC1/2. The potencies of these compounds are likely to be profoundly different *in vivo* compared with *in vitro* potencies in the absence of inositol phosphates. Our findings are supported by cell-based assays evaluating histone acetylation and HDAC degradation, highlighting the importance of evaluating HDACi in the context of HDAC complexes and inositol phosphates.

## Graphical abstract

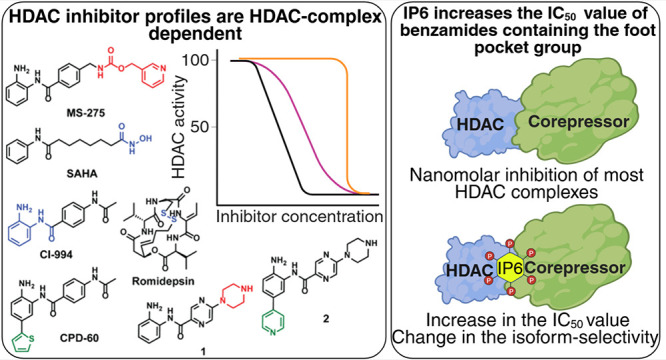

Histone deacetylases (HDACs) are important epigenetic erasers, removing acetyl groups from lysine residues in histone tails. This leads to a more compact chromatin state, prevents binding of acetyl-lysine readers, leaving lysines available for methylation.^1,2^ Dysregulation and abnormal HDAC expression have been implicated in cancer, cardiac diseases, and neurological disorders.^1,3,4^

Six HDAC inhibitors (HDACi) (vorinostat (SAHA), belinostat, panobinostat, romidepsin, chidamide, and givinostat) have been used to treat cutaneous and peripheral T-cell lymphoma, multiple myeloma and Duchenne muscular dystrophy.^5–10^ The majority of clinically approved HDACi have a hydroxamic acid zinc-binding group and inhibit all 11 zinc-dependent HDACs. None of the approved inhibitors exhibit isoform-specificity, likely contributing to their adverse side effects.^11,12^ Class I HDACs are attractive therapeutic targets, given their role in pathways contributing to oncogenesis, diabetes, cardiac disorders, and neurodegenerative diseases.^3,13,14^ However, targeting a specific class I HDAC is challenging due to the similarity of active sites and surrounding surfaces. A strategy to gain HDAC1/2 selectivity over HDAC3/8 exploits the so-called “foot-pocket”, an internal hydrophobic cavity in HDAC1&2. A tyrosine/tryptophan in HDAC3/8 (vs serine in HDAC1&2), partially occlude the cavity.^13–16^ Most of the HDACi with foot-pocket groups (FPG) are benzamides and have been reported to have nanomolar inhibition for HDAC1&2 and micromolar inhibition for HDAC3.^16,19^

In cells, HDAC1–3 are found in large, multiprotein complexes with HDAC1&2 forming part of the REST corepressor (CoREST), mitotic deacetylase complex (MiDAC), mesoderm induction early response (MIER), nucleosome remodelling deacetylase (NuRD), arginine glutamic acid repeat (RERE) and switch-independent 3 (SIN3) complexes, whereas HDAC3 solely exists in the nuclear receptor corepressor complex (NCoR/SMRT).^15,18–32^ These complexes possess distinct biological activities, with little redundancy, and different acetyl-lysine specificities within nucleosome substrates.^27,33–37^ The highest enzymatic activity requires HDACs to be associated with their complex partners.^17^ The additional protein subunits direct complexes to specific genomic loci and substrates.^33–35^ However, class I HDACi are rarely evaluated in the context of assembled HDAC complexes, potentially a critical oversimplification given that differences between complexes could have important implications in the development of HDAC-based therapies.^38–41^

Additionally, several studies have demonstrated the importance of inositol phosphates (IPs) in regulating HDAC1–3 activity.^17,18,23^ The structure of HDAC3-SMRT showed inositol 1,4,5,6-tetrakisphosphate bound in a conserved basic pocket sandwiched between HDAC3 and the corepressor.^17^ Further studies demonstrated that other class I HDAC complexes are activated by IPs *in vitro*, and that IP binding is conserved from yeast to man.^18,25,26,28,42,43^ Mutation of key IP binding residues in HDAC1 reduces enzymatic activity *in vivo*.^44^ Intriguingly, binding of HDACi enhances IP binding, which suggests a crosstalk between the two sites.^42^ This allosteric relationship may influence HDAC inhibition, yet class I HDACi have not been assessed in the presence of IPs. The concentration of IPs in cells is thought to be between 0 and 100 μM, in the range of the dissociation constant for HDAC complexes at physiological salt concentrations.^42,45^ Although the fraction of the various HDAC complexes bound to IPs under physiological conditions is not known, it is important to consider whether IPs affect inhibition of class I HDAC complexes.

Here, we report distinct inhibition profiles of class I-targeting HDACi against the seven HDAC1–3 corepressor complexes in the presence and absence of inositol hexaphosphate (InsP_6_). We assessed FDA-approved SAHA and romidepsin, and novel small molecules, thought to selectively inhibit HDAC1/2. These inhibitors showed markedly varied inhibition depending on the HDAC complex and we observed significant differences in inhibition when InsP_6_ was present, particularly very large increases in IC_50_ values (>1,000-fold) of benzamides bearing the FPG in the presence of InsP_6_.

Class I HDAC complexes were expressed and purified from mammalian HEK293F cells.^46^ We used HDAC complexes with the most components that could be stably expressed, with full-length proteins where feasible. We explored seven different HDAC complexes: five with HDAC1 (CoREST, MIER1, MiDAC, NuRD, and RERE); one with HDAC2 (CoREST), and one with HDAC3 (SMRT). Complexes ranged from having two components (HDAC1-RERE) to four components (HDAC1-NuRD) (Figure 1A, Table S1). The “standard” Boc-Lys-(Ac)-AMC peptide substrate assay revealed that the activity of all the complexes is similar (normalized by HDAC concentration), albeit the HDAC2-CoREST and HDAC1-MiDAC complexes showed somewhat lower activity (Figure 1B). In the presence of InsP_6_ all complexes exhibited increased activity.

**Figure 1:**
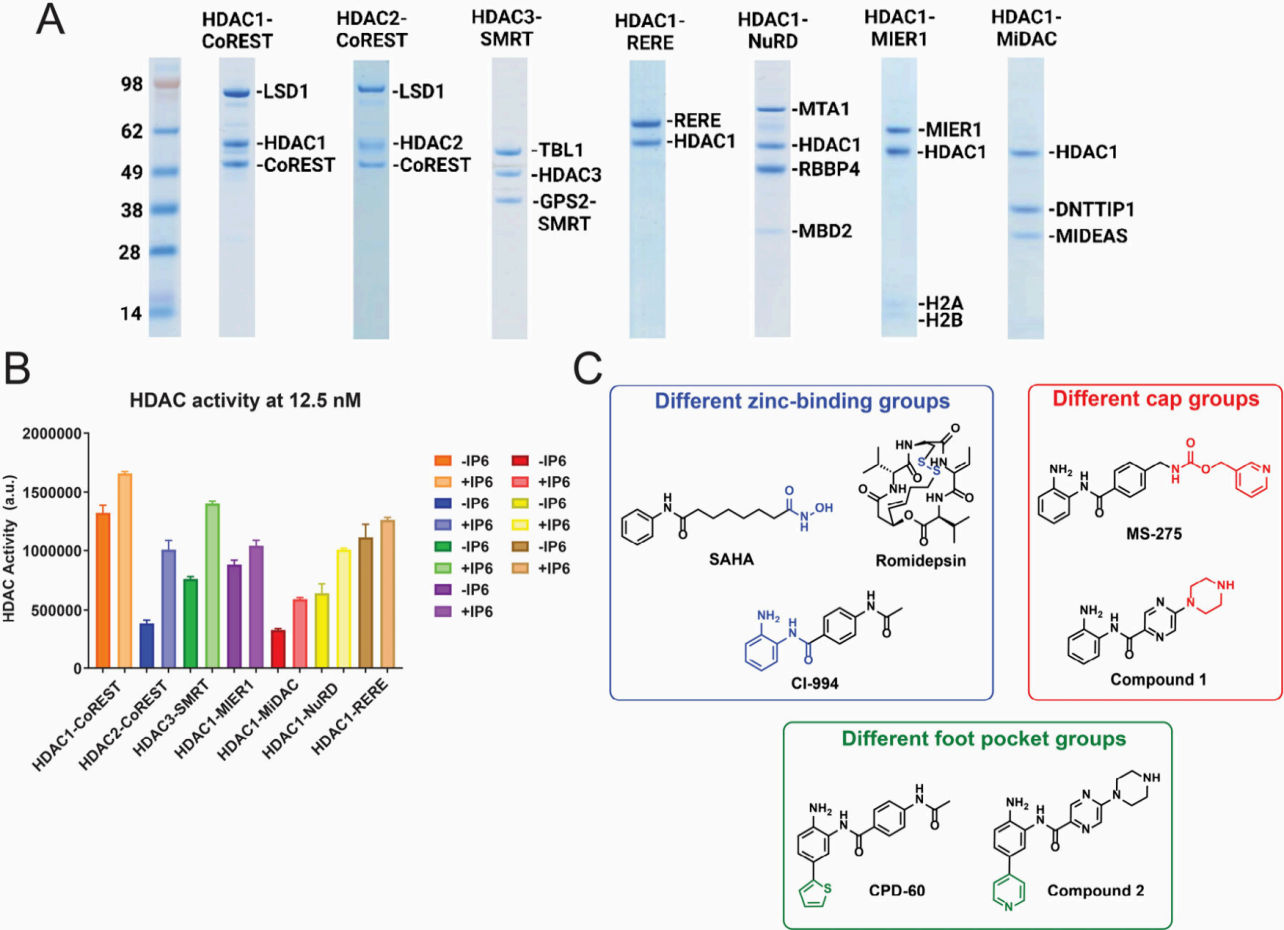
(A) SDS-PAGE of class I HDAC complexes. (B) Intrinsic HDAC activity of HDAC complexes at 12.5 nM (mean ± SEM, *n* = 3; technical replicates). (C) Chemical structures of HDACi. See Figure S25–39 for synthesis and characterization of compounds.

We investigated the inhibition of clinically relevant HDACi with hydroxamate, thiol, and benzamide zinc-binding groups (Figure 1C). The hydroxamic acid inhibitor SAHA inhibited all the complexes (IC_50_ = 28–100 nM), likely due to chemical similarity to the Boc-Lys-(Ac)-AMC substrate (Figure 2A). In contrast, romidepsin strongly favored HDAC1/2-CoREST and HDAC3-SMRT (IC_50_ = 1–3 nM) in comparison to other HDAC1-containing complexes (IC_50_ = 12–78 nM) (Figure 2B). This complex-selective inhibition is likely a consequence of the large cyclic surface group that may not be accommodated in all HDAC complexes. The benzamide-based MS-275 was a relatively potent inhibitor of the HDAC1/2-CoREST, HDAC3-SMRT, and HDAC1-MIER1 complexes (IC_50_ = 43–210 nM) (Figure 2C). However, unlike romidepsin, MS-275 showed comparatively weak inhibition of HDAC1-MiDAC, HDAC1-NuRD and HDAC1-RERE (IC_50_ = 5.8–29 μM). HDAC1-RERE was particularly refractory to inhibition by MS-275.

**Figure 2:**
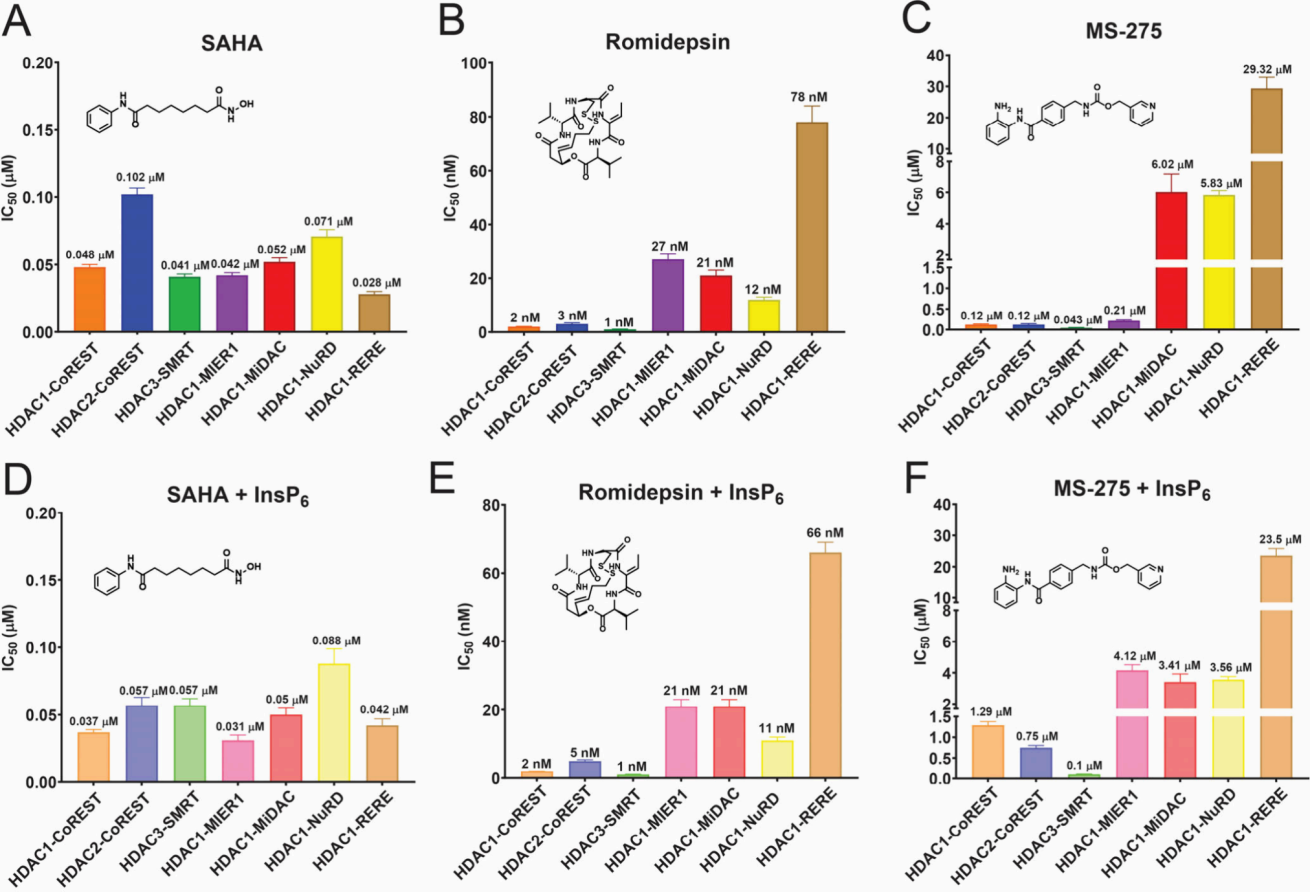
Inhibition of HDAC complexes with SAHA, Romidepsin and MS-275 ± InsP_6_ (mean ± SEM, *n* = 3; technical replicates). See also Figures S2–7.

To determine whether other benzamides would exhibit a similar pattern of specificity to MS-275, we tested the simpler benzamide (CI-994) and a novel inhibitor (compound 1), based on the (piperazin-1-yl) pyrazine benzamide scaffold.^47^ We found that the inhibition profiles were similar to MS-275 with the greatest potency against HDAC1/2-CoREST, HDAC3-SMRT, and HDAC1-MIER1, lower efficacy against HDAC1-MiDAC and HDAC1-NuRD and weak inhibition of HDAC1-RERE (Figure 3A,C). A chemoproteomic study showed similar observations regarding *ortho*-aminoanilines.^22,48^

**Figure 3:**
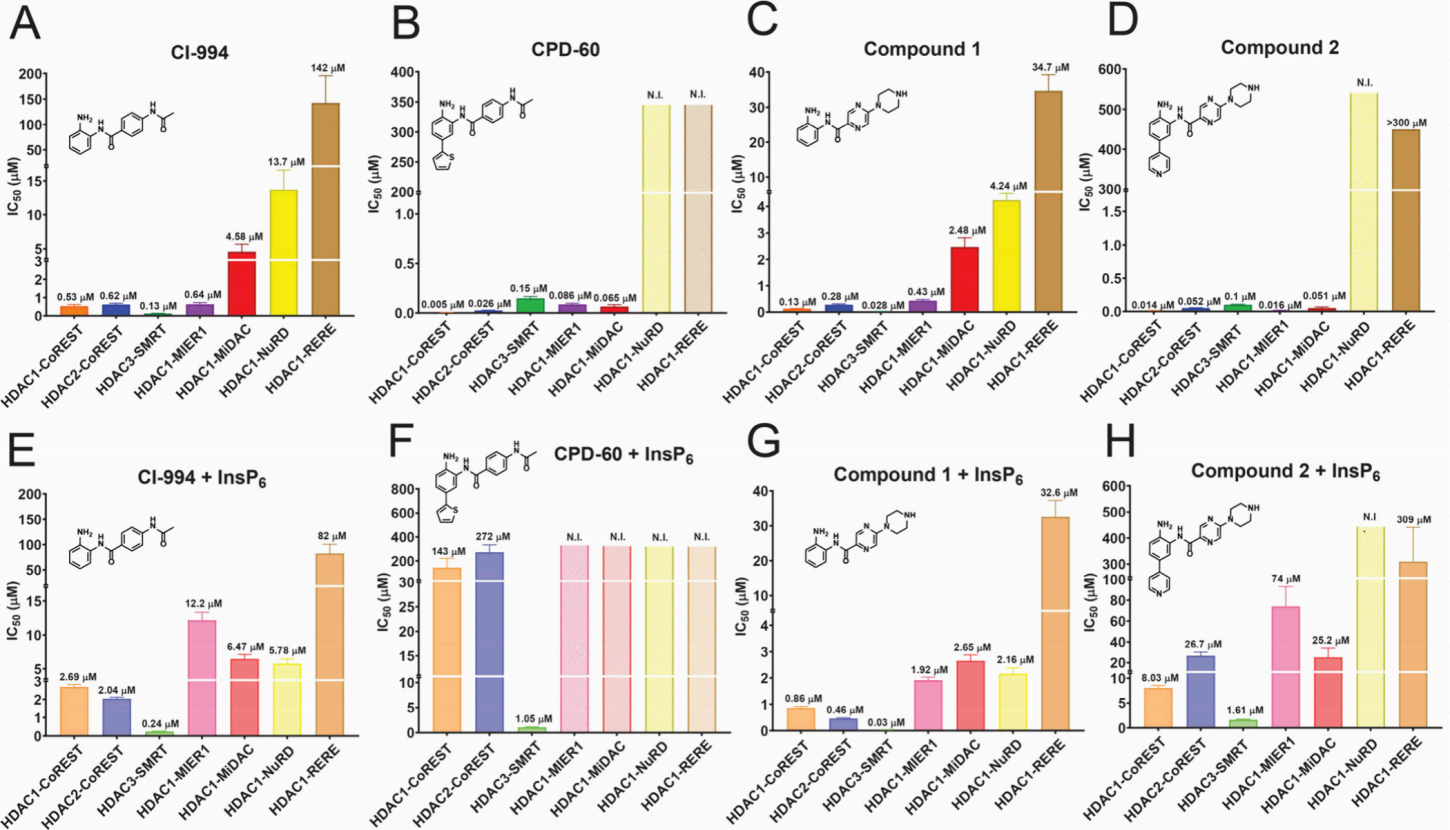
Inhibition of HDAC complexes with CI-994, CPD-60, and Compounds 1 and 2 (mean ± SEM, *n* = 3; technical replicates). See also Figures S8–15.

We synthesized two additional inhibitors with a FPG to evaluate their HDAC1/2 selectivity in a multiprotein environment. Compound 2, an analogue of compound 1, and CPD-60, based on CI-994 (Figure 1C), showed yet another pattern of inhibition (Figure 3B,D).^19,47,49^ As shown previously, the FPG inhibitors are significantly more potent (5–100 fold) than the parent compounds against HDAC1/2-CoREST, and HDAC1-MIER1.^16,47,50^ Strikingly, compound 2 and CPD-60 are highly effective inhibitors of HDAC1-MiDAC, whereas the parent compounds only inhibited moderately. Interestingly, the FPG inhibitors showed no detectable inhibition of HDAC1-NuRD and HDAC1-RERE, but they retained good inhibition of HDAC3-SMRT, in contrast to previous reports.^14,44–46^ This discrepancy may arise from using isolated HDAC3 or HDAC3 with the minimal activation domain of SMRT. Our findings align with the recent study by Payne et al. in that CPD-60 lacks HDAC1/2 selectivity over HDAC3. Both of our studies challenge the traditional understanding of how isoform-selective HDACi perform, revealing that the biological context critically influences perceived selectivity.^51^

We investigated the potencies of HDACi in the presence of InsP_6_, since it is present in mammalian cells and binds to HDAC complexes.^42,52^ To ensure saturation, 100 μM of InsP_6_ was used. For SAHA and romidepsin, we observed no significant effects of InsP_6_ (Figure 2D,E, Table 1). In contrast, there was a significant increase in the IC_50_ values of the benzamides (MS-275 and CI-994), with notable increases observed against HDAC1/2-CoREST and HDAC1-MIER1 (3.3–19 fold) (Figure 2F and Figure 3E). Inhibition of HDAC3-SMRT, HDAC1-MiDAC, HDAC1-NuRD, and HDAC1-RERE was not significantly affected by InsP_6_. Compound 1 exhibited the same inhibition pattern as CI-994 but was less sensitive to InsP_6_ (Figure 3G).

**Table 1:** Summary of the IC_50_ Values ± InsP_6_ ^a^

	IC_50_ (μM)					
HDAC Complex	SAHA		Romidepsin		MS-275	
	-IP6	+IP6	-IP6	+IP6	-IP6	+IP6
HDAC1- CoREST	**0.048** ± 0.002	**0.037** ± 0.002	**0.002** ± 0.0001	**0.002** ± 0.0001	**0.12** ± 0.02	**1.29** ± 0.09
HDAC2- CoREST	**0.1** ± 0.005	**0.057** ± 0.006	**0.003** ± 0.0004	**0.005** ± 0.0004	**0.12** ± 0.03	**0.75** ± 0.06
HDAC3- SMRT	**0.041** ± 0.002	**0.057** ± 0.005	**0.001** ± 0.0001	**0.001** ± 0.0001	**0.043** ± 0.01	**0.1** ± 0.01
HDAC1- MIER1	**0.042** ± 0.002	**0.031** ± 0.004	**0.027** ± 0.002	**0.021** ± 0.002	**0.21** ± 0.02	**4.12** ± 0.36
HDAC1- MiDAC	**0.052** ± 0.003	**0.05** ± 0.005	**0.021** ± 0.002	**0.021** ± 0.002	**6.02** ± 1.18	**3.41** ± 0.51
HDAC1- NuRD	**0.071** ± 0.005	**0.088** ± 0.011	**0.012** ± 0.001	**0.011** ± 0.001	**5.83** ± 0.28	**3.56** ± 0.2
HDAC1- RERE	**0.028** ± 0.002	**0.042** ± 0.005	**0.078** ± 0.006	**0.066** ± 0.003	**29.3** ± 3.6	**23.5** ± 2.26

a See also Figures S2–7.

Strikingly, we observed very large increases in the IC_50_ (275–28,600 fold) of benzamides bearing the FPG (compound 2 and CPD-60) against HDAC1/2-CoREST, HDAC1-MIER1, and HDAC1-MiDAC (Figure 3F,H). No detectable inhibition of HDAC1-MIER1 and HDAC1-MiDAC was observed with CPD-60. Remarkably, InsP_6_ also reversed the selectivity of compound 2 and CPD-60 for HDAC1/2 over HDAC3. Inhibition of HDAC3-SMRT only decreased by 3.5–16 fold compared with >500 fold decreases with complexes containing HDAC1/2 (Table 2).

**Table 2:** Summary of the IC_50_ Values ± InsP_6_ ^a^

	IC_50_ (μM)							
HDAC Complex	CI-994		CDP-60		Compound 1		Compound 2	
	-IP6	+IP6	-IP6	+IP6	-IP6	+IP6	-IP6	+IP6
HDAC1- CoREST	**0.53** ± 0.09	**2.69** ± 0.13	**0.005** ± 0.001	**143** ± 78	**0.13** ± 0.01	**0.86** ± 0.06	**0.014** ± 0.001	**8.03** ± 0.51
HDAC2- CoREST	**0.62** ± 0.07	**2.04** ± 0.08	**0.026** ± 0.002	**272** ± 62	**0.28** ± 0.03	**0.46** ± 0.03	**0.052** ± 0.003	**26.7** ± 3.53
HDAC3- SMRT	**0.13** ± 0.01	**0.24** ± 0.02	**0.15** ± 0.019	**1.05** ± 0.09	**0.028** ± 0.003	**0.03** ± 0.004	**0.1** ± 0.008	**1.61** ± 0.1
HDAC1- MIER1	**0.64** ± 0.09	**12.2** ± 1.16	**0.086** ± 0.012	N.I.	**0.43** ± 0.04	**1.92** ± 0.11	**0.016** ± 0.002	**74** ± 19
HDAC1- MiDAC	**4.58** ± 1.11	**6.47** ± 0.66	**0.065** ± 0.019	N.I.	**2.48** ± 0.35	**2.65** ± 0.23	**0.051** ± 0.017	**25.2** ± 8.84
HDAC1- NuRD	**13.7** ± 2.9	**5.78** ± 0.68	N.I.	N.I.	**4.24** ± 0.26	**2.16** ± 0.23	N.I.	N.I.
HDAC1- RERE	**142** ± 53	**82** ± 18	N.I.	N.I.	**34.7** ± 4.6	**32.6** ± 4.7	**>300**	**309** ± 131

a See also Figures S8–15.

To compare the efficacy of the benzamides and the FPG-benzamides in a cellular context, we monitored H3K56 acetylation.^39,40,53^ HCT116 cells were treated with increasing concentrations of pairs of related inhibitors (Figure 4A-D). Notably, the FPG-benzamides were 2–3 fold less effective at increasing maximal H3K56ac than the non-FPG-benzamides. Importantly, all four reached saturation at similar concentrations, suggesting no significant difference in cell permeability.

**Figure 4:**
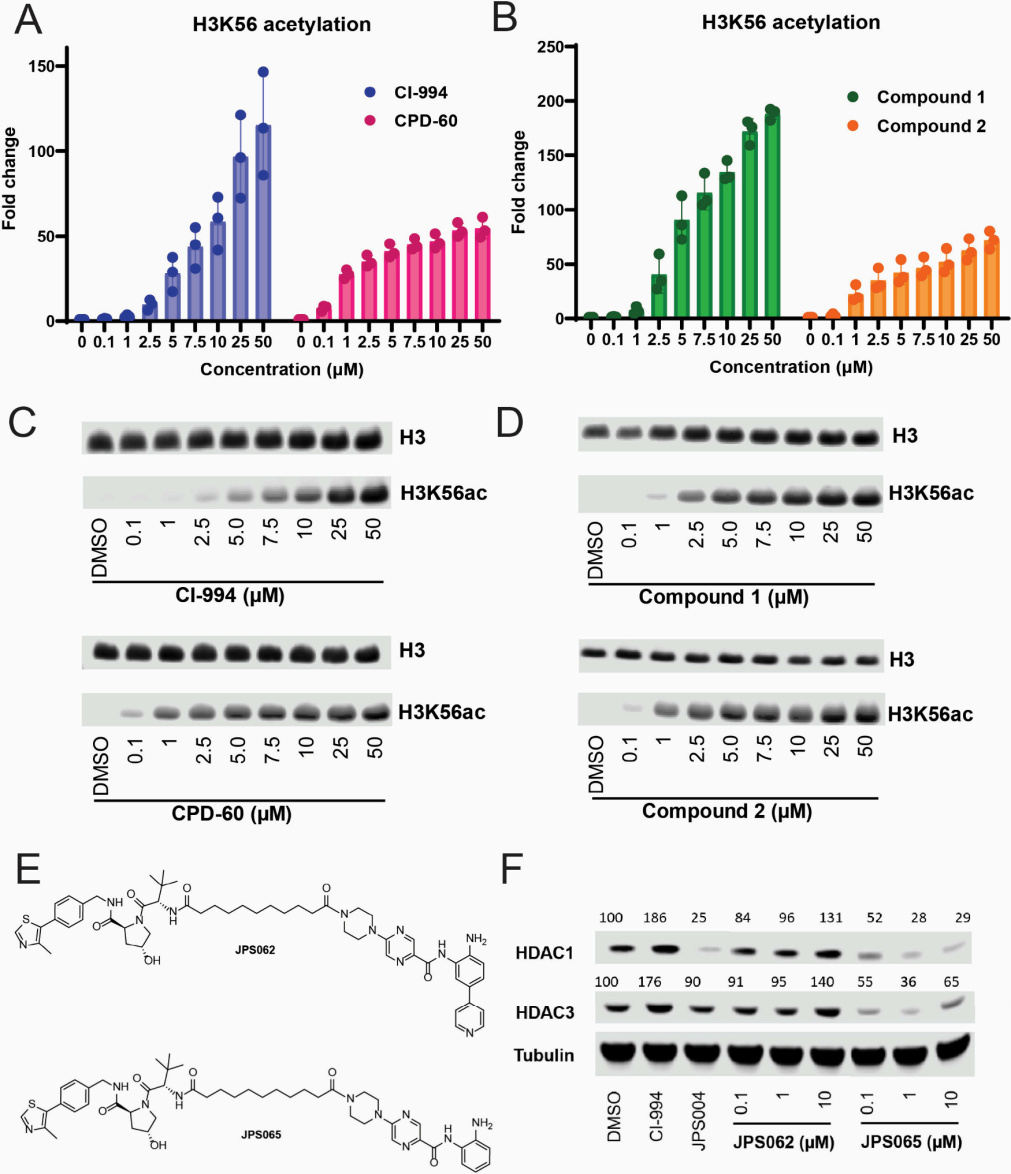
(A–D) Fold changes in H3K56ac levels in HCT116 cells following treatment with increasing concentrations of CI-994, CDP-60, compounds 1 and 2, and their representative immunoblots. Error bars represent SEM from three biological replicates. (E) PROTACs using compounds 1 and 2 as HDAC ligands. (F) Immunoblot showing protein levels of HDAC1, HDAC3, and α-tubulin in HCT116 cells following 24 h treatment with JPS062 and JPS065. Protein levels are compared to DMSO control. JPS004 is a previously HDAC1-3 PROTAC.^39^ See also Figures S16–24.

To confirm the surprising lack of efficacy of FPG-benzamides, we compared the degradation efficacy of two PROTACs (JPS065 based on compound 1 and JPS062 based on compound 2) in HCT116 cells (Figure 4E). At 1 μM, JPS065 showed significant degradation of HDAC1 (72%) and HDAC3 (64%) (Figure 4F). Remarkably, we observed no degradation of HDAC1 or HDAC3 (even at 10 μM) with JPS062. This finding mirrors the poor inhibition of H3K56 deacetylation by compound 2.

Here, we report that the efficacy of HDACi is highly dependent on the particular multiprotein environment. The diversity of inhibition is striking, particularly in the case of benzamides, despite the complexes sharing the same HDAC. Remarkably, FPG-benzamides show essentially no inhibition of HDAC1-NuRD and HDAC1-RERE. It has been previously shown that benzamides exhibit slow on/off kinetics and their binding leads to a rearrangement of the HDAC active site.^54,55^ The complex-dependent inhibition by benzamides is likely influenced by the complex partners changing the dynamic behavior of the HDAC catalytic unit. It is worth noting that our inhibition experiments used the minimal Boc-Lys-(Ac)-AMC substrate. Different inhibition profiles might be observed when using more physiological substrates such as acetylated histones or nucleosomes.

The finding that InsP_6_ significantly increases the IC_50_ values of benzamides has important implications for drug development. Particularly noteworthy is that the clinical candidate MS-275 exhibits markedly decreased potency in the presence of InsP_6_. Furthermore, FPG-benzamides showed striking increases in the IC_50_ values against all class I HDAC complexes, resulting in high micromolar inhibition values. Consistent with the *in vitro* data FPG-benzamides are less effective at increasing H3K56ac in HCT116 cells, suggesting a potential influence of IPs *in vivo*. Alternatively, the H3K56ac patterns may result from preferential targeting of a subset of HDAC complexes. An equally important observation is that in the absence of InsP_6_, FPG-benzamides possess some selectivity for HDAC1/2-containing complexes over HDAC3-SMRT, however in the presence of InsP_6_, the isoform-selectivity is flipped. This raises the question of the HDAC1/2-selectivity of these ligands *in vivo*.

It has been suggested that the foot-pocket is a transit route for acetate and/or water during the deacetylation reaction.^56–59^ Strikingly, residues in the foot pocket cavity and residues that interact with IP, are very close to each other, forming part of the same loop (Figure 5). Functional studies shown that IP increases HDAC activity and deacetylation efficiency, however no structural changes were observed upon InsP_6_ binding.^17,42^ Hence, it is likely that the mechanism of IP activation is entropically driven, altering the dynamics of the HDAC complex and HDAC active site, particularly the foot-pocket, and thereby the dynamics of inhibitor binding.^42^

**Figure 5:**
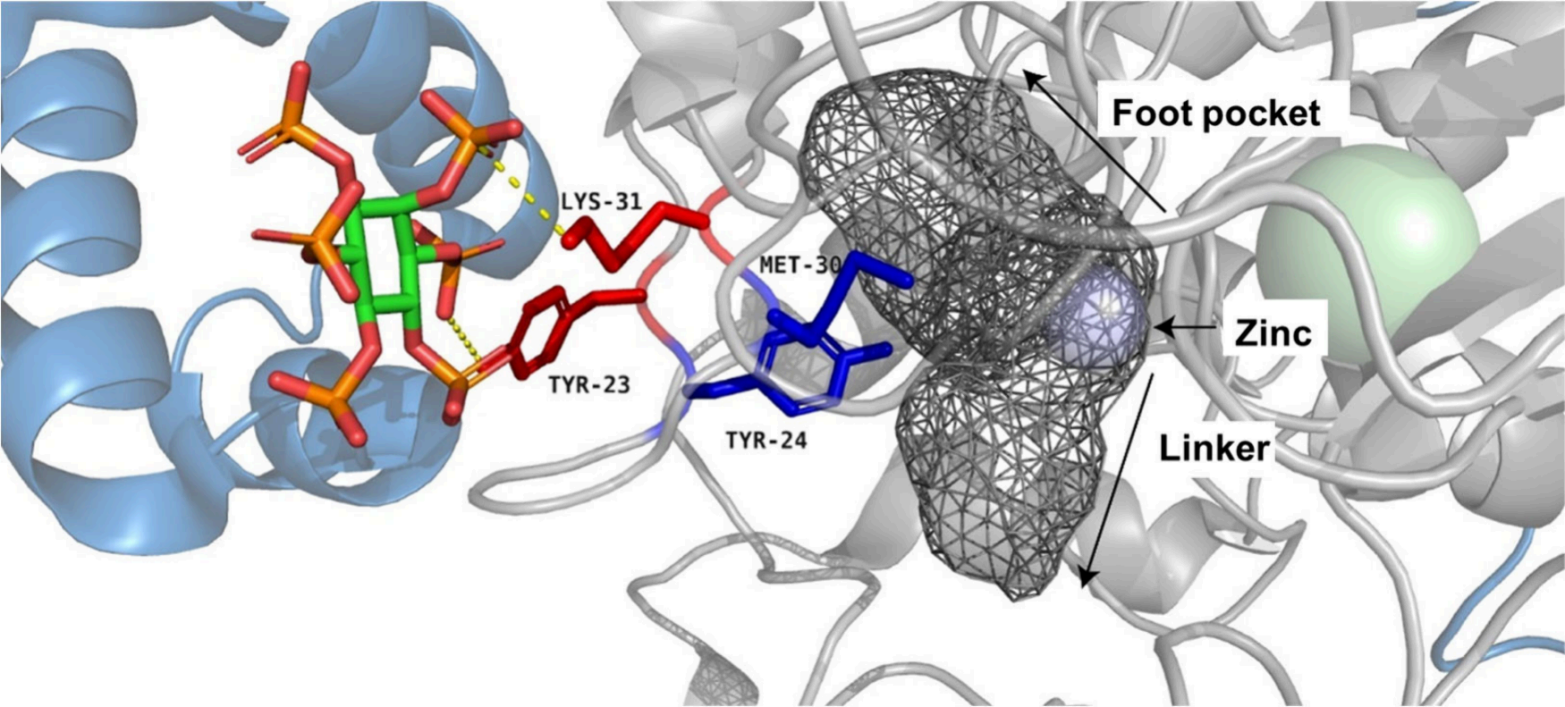
Published HDAC1-MTA1­(ELM2-SANT) structure with InsP_6_ (PBD:5ICN).^18^ The figure shows residues interacting with InsP_6_ (red), foot-pocket residues (blue), and the active site cavity as a mesh.

Numerous FPG-benzamides have been developed as potential HDAC1/2-selective inhibitors. We found that IP not only significantly reduces their potency, but also reverses their isoform-selectivity pattern. Therefore, such compounds may not be the most effective HDAC ligands *in vivo*, where the concentration of IPs can reach 100 μM.^45,52^ Importantly, HDAC1-NuRD and HDAC1-RERE are not inhibited by the FPG-benzamides. This is especially important since the NuRD complex is highly abundant in cells.^60^ Overall, this study highlights the importance of evaluating HDACi against class I HDACs that are assembled into their specific complexes since the different partner proteins and IPs strongly influence the potency and selectivity of HDACi.

## Supplementary Material



## Notes

Details of chemical syntheses and analyses, protein expression and purification, original inhibition curves, etc. (PDF)
