# Supplementary material for: The Allosteric Regulator Inositol Phosphate Dramatically Affects the Efficacy and Selectivity of Inhibitors for Different HDAC Complexes
Source: J Am Chem Soc. 2025 Sep 25;147(40):36044–52. doi: 10.1021/jacs.5c08929 (PMC12512194; doi:10.1021/jacs.5c08929)
Supplement: Supplementary file 1 [file ja5c08929_si_001.pdf]

Supporting Information

The allosteric regulator inositol phosphate dramatically affects the efficacy and selectivity of inhibitors for different HDAC complexes

Wiktoria A. Pytel[1,2], Urvashi Patel[1,3], Joshua P. Smalley[1,3], Christopher J. Millard[1,2] Edward A. Brown[1,2], Aline R. Pavan[3,4], Siyu Wang[1,2], Jay H. Kalin[5], Jean Leandro dos Santos[4], Philip A. Cole[5], James T. Hodgkinson[1,3]*, John W.R. Schwabe[1,2]*

1. Institute for Structural and Chemical Biology, University of Leicester, LE1 7RH. UK.

2. Department of Molecular and Cell Biology, University of Leicester, LE1 7RH. UK.

3. School of Chemistry, University of Leicester, LE1 7RH. UK.

4. São Paulo State University (UNESP), School of Pharmaceutical Sciences, 14800-903 Araraquara, Brazil.

5. Division of Genetics, Department of Medicine, Brigham and Women's Hospital and Department of Biological Chemistry and Molecular Pharmacology, Harvard Medical School, Boston, MA 02115. USA.

*Co-corresponding authors

Table of Content

Supporting Information 1
Table of Content 1
Supporting Methods: Chemistry 2
1. General Information 2
2. Synthesis of HDACi 4
2.1 CI-994 4
2.2 CPD-60 8
2.3 Compound 1 13
2.4 Compound 2 17
3. Synthesis of PROTACs 22
Supporting Methods - Biology 27
4. Expression and purification of class I HDAC complexes in HEK293F cells 27
4.1 Culture of HEK293F cells 27
4.2 Transfection 27
4.3 Protein Purification 27
5. HDAC assay 28
6. Culture of HCT116 cells 29
7. Compound treatment of HCT116 cells 29
8. Protein Extraction 29
9. Western Blotting 30
Supporting Figures and Tables 31
Components of HDAC complexes 31
Inhibition Curves 33
Processed histone acetylation immunoblots 47
Unprocessed immunoblots 51
Analytical data of HDAC inhibitors 56
References: 63

Supporting Methods: Chemistry

1. General Information

All solvents and reagents were obtained from Fisher Scientific, Sigma Aldrich, Acros Organics and Fluorochem. SAHA, MS-275, and Romidepsin were purchased from MedChemExpress. VH 032 amide-alkylC9-acid was acquired from Tocris Bioscience. Macroporous polystyrene-co-divinylbenzene (MP) carbonate resin (3.02 mmol/g loading capacity) was used for neutralizing amine TFA salts and scavenging excess TFA during Boc-deprotection reactions. For moisture sensitive reaction, the required glassware was dried in oven at 100 °C for 12 hours and anhydrous solvents were used. Dry DCM and THF were dried *via* Innovative Technology inc. PureSolv solvent purification system. Unless otherwise stated reactions, were performed under nitrogen. Room temperature indicates ambient temperature. Temperatures of 0 °C were maintained using an ice-water bath. Analytical TLCs were run on aluminium backed silica gel or neutral alumina plates. Purifications were conducted through flash column chromatography with Silica Gel 60 (230-400 mesh) or neutral activated Brockmann I grade alumina (150 mesh), using commercial solvents. Unless otherwise stated, purifications were performed with Silica Gel 60. All evaporations *in vacuo* were carried out under reduced pressure utilising a Büchi rotary evaporator. Chemical names of synthesised compounds have been generated using ChemDraw Professional.

NMR spectroscopy (^{1}H and ^{13}C) were recorded on Bruker AV400 machine at ambient temperatures in deuterated solvents ($CDCl_3$, MeOD-d_4 or DMSO-d_6). The solvent used is outlined in the individual compound data. ^{1}H NMR chemical shifts (δ) are written in parts per million (ppm) and reported to the nearest 0.01 ppm with tetramethylsilane used as a reference. Coupling constants (J) are calculated in Hertz (Hz) to the nearest 0.1 Hz and found using ACD Labs/Spectrus Processor 2019.1.1. ^{13}C NMR spectra were recorded through broadband proton decoupling. ^{13}C NMR chemical shifts (δ) are written in ppm and are reported to the nearest 0.1 ppm. NMR data are reported in the following order: chemical shift, integration, multiplicity (b, broad; s, singlet; d, doublet; t, triplet; q, quartet; quin, quintet; sept, septet; m, multiplet) or as a combination (dd, doublet of doublets; dt, doublet of triplets;), coupling constant(s) and individual atom assignments. 2D NMR spectroscopy (COSY, HSQC, HMBC) was used to fully assign the ^{1}H NMR and ^{13}C NMR spectra. Mass spectrometry was performed on a Micromass Quatro LC electrospray spectrometer. HRMS was conducted on a Waters Acquity XEVO Q Time of flight spectrometer with either electrospray (ES+) or Atmospheric Solids Analysis Probe (ASAP) ionisation. Analytical HPLC was conducted using an UltiMate 3000 Diode Array Detector (Thermo Scientific) monitoring UV and Visible wavelengths coupled to an UltiMate 3000 HPLC system (Thermo Scientific) with a Luna LC C18 Column (100 Å, 5 µm, 250 x 4.6 mm, Phenomenex). The flow rate was 1 mL/min, and a gradient of: 95% Solvent A (5% acetonitrile in water) with 95% Solvent B

(5% water in acetonitrile) held constant for 5 minutes, followed by a linear gradient of 100% Solvent B over 30 minutes. 20 μL of sample was injected per run, with detectors monitoring at wavelengths: 214 nm, 260 nm, 310 nm, and 330 nm. All samples were dissolved in HPLC grade methanol.

2. Synthesis of HDACi

2.1 CI-994

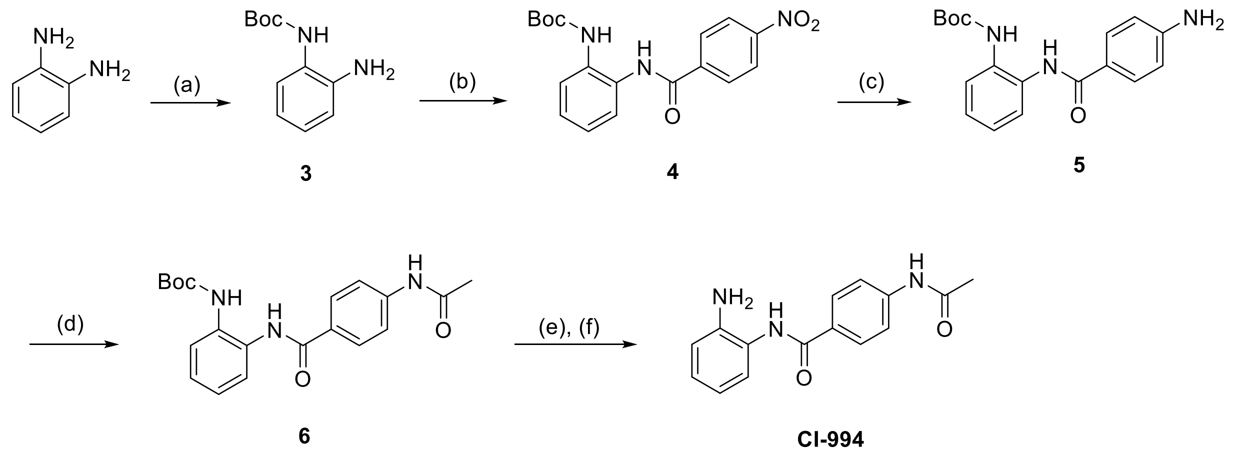

Scheme 1 - Synthesis of CI-994. Reagents and conditions: (a) Boc_2O, NEt_3, THF, 0 °C to rt, 18 h, 70%; (b) 4-nitrobenzoyl chloride, DIPEA, DCM, 0 °C to rt, 16 h, 74 %; (c) H_2, 10% Pd/C, MeOH/THF (1:1), rt, 17 h, 95 %; (d) AcCl, NEt_3, THF, 0 °C to rt, 21 h, 67 %; (e) TFA, DCM, 0 °C to rt, 20 h; (f) MP-carbonate resin, MeOH, rt, 3 h, 95%.

Tert-butyl (2-aminophenyl) carbamate (3)

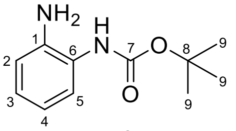

3

A solution of di-tert-butyldicarbonate (6.11 g, 28.0 mmol) in THF (50 mL) was added dropwise over 3 hours to a solution of o-phenylenediamine (3.07 g, 28.4 mmol) and triethylamine (4.6 mL, 33.0 mmol) in THF (50 mL) at 0 °C. The mixture was stirred at room temperature for 19 hours. Reaction mixture was concentrated *in vacuo* to afford a grey/white/yellow crystalline solid. The solid was re-dissolved in EtOAc, washed with water (2 x 40 mL) and saturated NaCl (2 x 40 mL), filtered over Na_2SO_4, and concentrated *in vacuo* to afford a pale yellow/grey solid. The crude solid was purified by flash column chromatography (dry load, 25% EtOAc in hexane) to yield **3** as a white/cream solid (4.08 g, 13.5 mmol, 70%).

^{1}H NMR (400 MHz, $CDCl_3$) δH ppm 7.29 (d, J = 8.0 Hz, 1H, H5), 7.01 (td, J = 7.7 Hz, 1.5 Hz, 1H, H3), 6.83-6.76 (m, 2H, H2, H4), 6.33 (br s, 1H, NH), 3.76 (br s, 2H, NH_2), 1.54 (s, 9H, H9).

^{13}C NMR (100 MHz, $CDCl_3$) δC ppm 153.9 (C7), 140.0 (C1), 126.2 (C3, C6), 124.8 (C5), 119.6 (C4), 117.6 (C2), 80.5 (C8), 28.4 (C9).

HRMS (ESI) m/z: $[M+H]^+$ calculated for $C_{11}H_{17}N_2O_2$: 209.1290, found 209.1290.

***Tert*-butyl (2-(4-nitrobenzamido)phenyl)carbamate (4)**

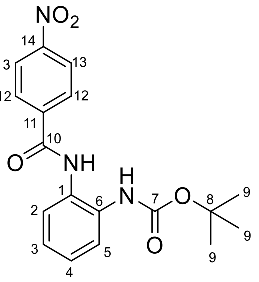

4

3 (2.95 g, 14.2 mmol) was dissolved in dry DCM (90 mL) and the solution was cooled down to 0 °C. DIPEA (4 mL, 23.0 mmol) was added and the solution was stirred for 20 minutes at 0 °C. Next, 4-nitrobenzoyl chloride (2.93 g, 15.8 mmol) dissolved in DCM (30 mL) was added dropwise to the reaction mixture at 0 °C and stirred for 20 minutes at 0 °C and then at room temperature for 16 hours. The reaction mixture was then diluted with more DCM (20 mL) and washed with saturated $NaHCO_3$ (100 mL), 1M HCl (100 mL) and saturated NaCl (100 mL). The organic layer was dried over Na_2SO_4 and concentrated *in vacuo* to yield a crude yellow solid (5.41 g, 15.1 mmol). The crude product was triturated in EtOH and then filtered to afford **4** as a pale-yellow solid (3.66 g, 10.2 mmol, 74%).

^{1}H NMR (400 MHz, $CDCl_3$) δH ppm 9.76 (br s, 1H, NH), 8.32 (app d, J = 8.9 Hz, 2H, H13), 8.15 (d, J = 8.7 Hz, 2H, H12), 7.92 (d, J = 8.2 Hz, 1H, H2), 7.29-7.25 (m, 1H, H4), 7.20-7.14 (m, 2H, H3, H5), 6.75 (br s, 1H, NH), 1.53 (s, 9H, H9).

^{13}C NMR (100 MHz, $CDCl_3$) δC ppm 163.2 (C10), 155.0 (C7), 149.8 (C11), 140.0 (C14), 130.7 (C6), 129.4 (C1), 128.6 (C12), 126.4 (C4), 126.3 (C3) 125.9 (C2), 124.4 (C5), 123.8 (C13), 82.0 (C8), 28.3 (C9).

HRMS (ESI) m/z: $[M+Na]^+$ calculated for $C_{18}H_{19}N_3O_5Na$: 380.1222, found 380.1223.

***Tert*-butyl (2-(4-aminobenzamido)phenyl)carbamate (5)**

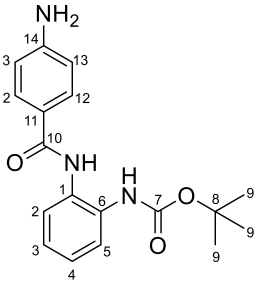

5

4 (2.90 g, 8.1 mmol) was dissolved in MeOH/THF (1:1, 80 mL) and 10% wt Pd/C (0.30 g) was added. The reaction flask was evacuated and filled with nitrogen three times using a Schlenk line. Next, a

balloon of hydrogen was added and the reaction mixture was stirred vigorously for 17 hours. The balloon of hydrogen was removed and the flask was evacuated and filled with nitrogen. The reaction mixture was filtered through celite and then the celite was washed further with MeOH (3 x 50 mL). The filtrate was concentrated *in vacuo* to yield **5** as a white crystalline solid (2.51 g, 7.7 mmol, 95%).

^{1}H NMR (400 MHz, $CDCl_3$) δH ppm 8.77 (br s, 1H, NH), 7.79 (app. d, *J* = 8 Hz, 2H, H12), 7.69 (dd, *J* = 7.8 Hz, 1.3 Hz, 1H, H5), 7.30 (dd, *J* = 7.8 Hz, 1.6 Hz, 1H, H2), 7.21-7.12 (m, 2H, H3, H4), 6.86 (br s, 1H, NH), 6.69 (app d, *J* = 8.0 Hz, 2H, H13), 4.02 (br s, 2H, H15), 1.51 (s, 9H, H9).

^{13}C NMR (100 MHz, $CDCl_3$) δC ppm 165.5 (C10), 154.5 (C7), 150.0 (C11), 131.2 (C1), 130.1 (C6), 129.3 (C12), 125.9 (C5), 125.7 (C4, C3), 124.5 (C2), 123.7 (C14), 114.1 (C13), 81.1 (C8), 28.3 (C9).

HRMS (ESI) m/z: $[M+Na]^+$ calculated for $C_{18}H_{21}N_3O_3Na$: 350.1481, found 350.1483.

***Tert*-butyl (2-(4-acetamidobenzamido)phenyl)carbamate (6)**

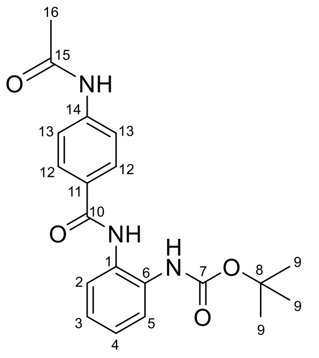

6

5 (0.16 g, 0.5 mmol) was dissolved in dry THF (10 mL) and the reaction mixture was cooled to 0 ˚C. NEt_3 (0.2 mL, 1.4 mmol) was then added at 0 ˚C, followed by a dropwise addition of acetyl chloride (0.05 mL, 0.7 mmol). The resultant mixture was stirred at 0 ˚C for 15 minutes and then at room temperature for 21 hours. The reaction mixture was then concentrated *in vacuo* to yield a crude white solid (0.40 g, 1.1 mmol). The crude product was purified by flash column chromatography (dry load, 100 % EtOAc) to afford **6** as a white solid (0.12 g, 0.3 mmol, 67%).

^{1}H NMR (400 MHz, DMSO-d_6) δH ppm 10.26 (s, 1H, NH), 9.76 (s, 1H, NH), 8.70 (br s, 1H, NH), 7.92 (d, *J* = 8.7 Hz, 2H, H12), 7.73 (d, *J* = 8.7 Hz, 2H, H13), 7.55-7.51 (m, 2H, H2, H5), 7.21-7.13 (m, 2H, H3, H4), 2.09 (s, 3H, H16), 1.45 (s, 9H, H9).

^{13}C NMR (100 MHz, DMSO-d_6) δC ppm 169.3 (C15) 165.2 (C9), 154.0 (C7), 143.0 (C14), 132.1 (C1), 130.4 (C6), 129.0 (C12), 128.7 (C11), 126.5 (C2/5), 126.0 (C3/4), 124.6 (C2/5), 124.4 (C3/4), 118.6 (C13), 80.1 (C8), 28.5 (C9), 24.6 (C16).

HRMS (ESI) m/z: $[M+H]^+$ calculated for $C_{20}H_{24}N_3O_4$: 370.1767, found 370.1765.

4-acetamido-N-(2-aminophenyl)benzamide (CI-994)

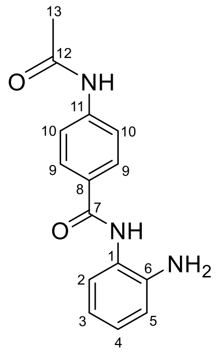

CI-994

6 (0.08 g, 0.2 mmol) was dissolved in dry DCM (15 mL) and the flask was cooled down to 0 ˚C. Next, TFA (0.2 mL, 2.6 mmol) was added dropwise at 0 ˚C and the reaction mixture was stirred for 20 hours. After an overnight stir, the reaction was concentrated *in vacuo* to afford a brown oil with a white precipitate (0.13 g, 0.5 mmol). The compound was re-dissolved in MeOH (25 mL) and agitated in MP-carbonate resin (3.02 mmol/g loading capacity, 0.35 g) for 3 hours. The reaction mixture was then filtered and the filtrate was concentrated *in vacuo* to yield crude **CI-994** as a pale yellow/white solid (0.07 g, 0.2 mmol). The crude product was purified on column chromatography (dry load, silica, 1% MeOH in EtOAc) to afford pure **CI-994** as a white solid (0.06 g, 0.2 mmol, 95%).

^{1}H NMR (400 MHz, DMSO-d_6) δH ppm 10.19 (s, 1H, NH), 9.56 (s, 1H, NH), 7.94 (d, *J* = 8.7 Hz, 2H, H9), 7.69 (d, *J* = 8.7 Hz, 2H, H10), 7.16 (d, *J* = 7.7 Hz, 1H, H2), 6.96 (t, *J* = 7.7 Hz, 1H, H4), 6.78 (dd, *J* = 8.0 Hz, 1.3 Hz, 1H, H5), 6.59 (t, *J* = 7.6 Hz, 1H, H3), 4.87 (s, 2H, NH_2), 2.09 (s, 3H, H13).

^{13}C NMR (100 MHz, DMSO-d_6) δC ppm 169.2 (C12) 165.2 (C7), 143.6 (C6), 142.6 (C11), 129.2 (C8), 129.1 (C9), 127.1 (C2), 126.8 (C4), 124.0 (C1), 118.5 (C10), 116.7 (C3), 116.6 (C5), 24.6 (C13).

HRMS (ESI) m/z: $[M+Na]^+$ calculated for $C_{15}H_{15}N_3O_2Na$: 292.1062, found 292.1061.

Spectroscopic data consistent with literature.[1]

2.2 CPD-60

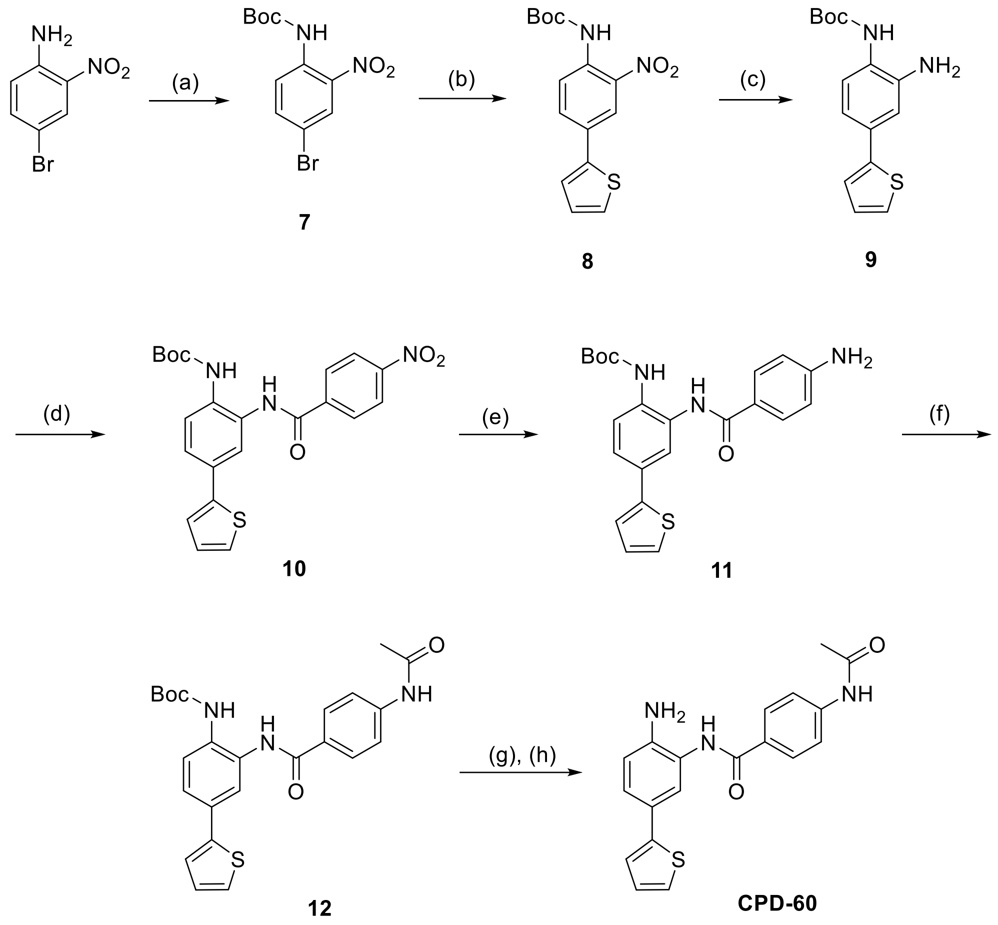

Scheme 2 – Synthesis of CPD-60. Reagents and conditions: (a) Boc_2O, NEt_3, DMAP, THF, 0-60 °C, 16 h, 47%; (b) Thiophen-2-ylboronic acid, $Pd(PPh_3)_4$, Na_2CO_3, DME/H_2O (2:1), 110 °C, 16 h, 74%; (c) H_2, 10% Pd/C, MeOH, rt, 4 h, 98 %; (d) 4-nitrobenzoyl chloride, DIPEA, DCM, 0 °C to rt, 16 h, 76 %; (e) $SnCl_2$, MeOH/DCM (1:1), rt, 1 week, 82 %; (f) AcCl, NEt_3, THF, 0 °C to rt, 3 h, 68 %; (g) TFA, DCM, 0 °C to rt, 4 h; (h) MP-carbonate resin, MeOH, rt, 3 h, 93 %.

Tert-butyl (4-bromo-2-nitrophenyl)carbamate (7)

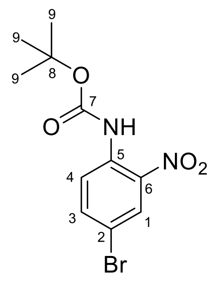

7

A solution of di-tert-butyldicarbonate (2.21 g, 10.14 mmol) in THF (5 mL) was added dropwise to a solution of 4-bromo-2-nitroaniline (2.00 g, 9.22 mmol), DMAP (0.11 g, 0.92 mmol) and triethylamine

(1.54 mL, 11.06 mmol) in THF (35 mL) at 0 °C. The mixture was stirred at 60 °C for 16 hours. The reaction mixture was concentrated *in vacuo* and then purified by column chromatography (0- 50% EtOAc in hexane) to afford **7** as a yellow crystalline solid (1.52 g, 4.76 mmol, 47%).

^{1}H NMR (400 MHz, $CDCl_3$) δH ppm 9.61 (br s, 1H, NH), 8.52 (d, *J* = 9.2 Hz, 1H, H4), 8.34 (d, *J* = 2.4 Hz, 1H, H1), 7.69 (dd, *J* = 9.2, 2.4 Hz, 1H, H3), 1.55 (s, 9H, H9).

^{13}C NMR (101 MHz, $CDCl_3$) δC ppm 151.9 (C7), 138.5 (C3), 136.1 (C6), 135.1 (C5), 128.2 (C1), 122.2 (C4), 113.7 (C2), 82.3 (C8), 28.1 (C9).

HRMS (ESI) m/z: $[(M\text{-}Boc)+H]^+$ calculated for $C_6H_6BrN_2O_4$ (^{79}Br): 216.9613, found 216.9615. HRMS (ESI) m/z: $[(M\text{-}Boc)+H]^+$ calculated for $C_6H_6BrN_2O_4$ (^{81}Br): 218.9592, found 218.9594.

Tert-butyl (2-nitro-4-(thiophen-2-yl)phenyl)carbamate (8)

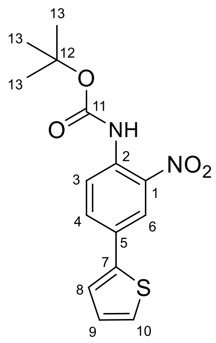

8

To a solution of DME/water (2:1, 36 mL) was added thiophen-2-ylboronic acid (0.77 g, 6.05 mmol), 31 (1.60 g, 5.05 mmol), Na_2CO_3 (0.80 g, 7.57 mmol) and $Pd(PPh_3)_4$ (0.38 g, 0.33 mmol). The resulted mixture was stirred vigorously at 110 °C for 16 hours. The reaction mixture was diluted with more water (40 mL) and then the product was extracted with EtOAc (3 x 50 mL). The organic layers were combined, washed with water (2 x 70 mL), dried over $MgSO_4$, filtered and concentrated *in vacuo* to afford a brown solid (1.92 g). The crude product was purified by column chromatography (dry load, 0-50% EtOAc in hexane) to give **8** as an orange crystalline solid (1.21 g, 3.74 mmol, 74%).

^{1}H NMR (400 MHz, $CDCl_3$) δH ppm 9.66 (s, 1H, NH), 8.60 (d, *J* = 8.9 Hz, 1H, H3), 8.40 (d, *J* = 2.1 Hz, 1H, H6), 7.82 (dd, *J* = 8.9, 2.1 Hz, 1H, H4), 7.32 - 7.36 (m, 2H, H8, H10), 7.11 (dd, *J* = 5.1, 3.7 Hz, 1H, H9), 1.57 (s, 9H, H13).

^{13}C NMR (101 MHz, $CDCl_3$) δC ppm 152.1 (C11), 141.3 (C7), 136.0 (C1), 134.8 (C2), 132.9 (C4), 128.7 (C5), 128.3 (C9), 125.6 (C10), 123.9 (C8), 122.3 (C6), 121.2 (C3), 82.0 (C12), 28.2 (C13).

HRMS (ESI) m/z: $[M\text{-}H]^-$ calculated for $C_{15}H_{15}N_2O_4S$: 319.0753, found 319.0753.

Tert-butyl (2-amino-4-(thiophen-2-yl)phenyl)carbamate (9)

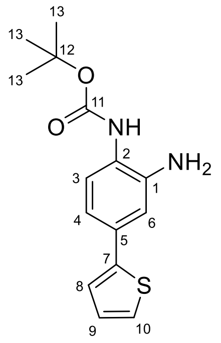

9

To a solution of **8** (1.18 g, 3.69 mmol) in MeOH (15 mL), 10% Pd/C (0.12 g) was added. The reaction flask was filled with nitrogen and evacuated 3 times using a Schlenk line, before a balloon of hydrogen was added and the resultant mixture stirred vigorously for 4 hours. The balloon of hydrogen was removed and the flask was flushed with nitrogen. The reaction mixture was filtered through a glass microfiber filter and then the filtrate was concentrated *in vacuo* to afford **9** as a brown solid (1.06 g, 3.60 mmol, 98%).

^{1}H NMR (400 MHz, $CDCl_3$) δH ppm 7.28-7.31 (m, 1H, H3), 7.24 (dd, *J* = 5.1, 1.1 Hz, 1H, H8), 7.22 (dd, *J* = 3.6, 1.1 Hz, 1H, H10), 7.01 - 7.09 (m, 3H, H4, H6, H9), 6.35 (br s, 1H, NH), 3.53 (br s, 2H, NH_2), 1.52 (s, 9H, H13).

^{13}C NMR (101 MHz, $CDCl_3$) δC ppm 153.8 (C11), 144.0 (C7), 139.5 (C1), 132.3 (C5), 127.9 (C9), 124.9 (C3), 124.5 (C8), 122.8 (C10), 120.7 (C8), 117.8 (C4), 115.2 (C6), 80.9 (C12), 28.3 (C13).

HRMS (ESI) m/z: $[M+H]^+$ calculated for $C_{15}H_{19}N_2O_2S$: 291.1167, found 291.1167.

Tert-butyl (2-(4-nitrobenzamido)-4-(thiophen-2-yl)phenyl)carbamate (10)

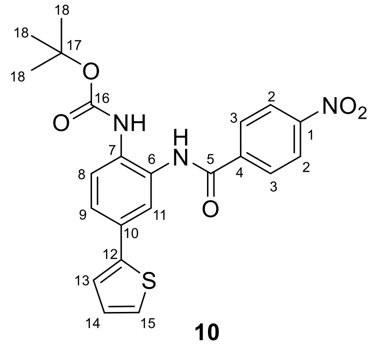

10

DIPEA (0.4 mL, 2.1 mmol) was added to a solution of **9** (0.4 g, 1.38 mmol.) in dry DCM (3 mL) at 0 °C, followed by the dropwise addition of 4-nitrobenzoyl chloride (0.28 g, 1.53 mmol) as a solution in dry DCM (2 mL). The mixture was stirred at 0 °C for 30 minutes, then at room temperature for 16 hours. The reaction mixture was diluted with DCM (10 mL) and then washed with sat. $NaHCO_4$ (2 x 20

mL), 1M HCl (2 x 20 mL) and sat. NaCl (20 mL). The organic layer was then dried over Na_2SO_4 and concentrated *in vacuo*. then purified accordingly to afford the desired compound. The crude product was triturated in EtOH and then filtered to afford **10** as a pale green solid (0.46 mg, 1.04 mmol, 76%).

^{1}H NMR (400 MHz, $CDCl_3$) δH ppm 10.19 (s, 1H, NH), 8.81 (s, 1H, NH), 8.40 (d, J = 8.8 Hz, 2H, H2), 8.23 (d, J = 8.8 Hz, 2H, H3), 7.79 (d, J = 2.0 Hz, 1H, H11), 7.70 (d, J = 8.4 Hz, 1H, H8), 7.54 (dd, J = 8.4, 2.0 Hz, 1H, H9), 7.52 (dd, J = 5.0, 1.0 Hz, 1H, H13), 7.46 (dd, J = 3.6, 1.0 Hz, 1H, H15), 7.13 (dd, J = 5.0, 3.6 Hz, 1H, H14), 1.45 (s, 9H, H18).

^{13}C NMR (101 MHz, $CDCl_3$) δC ppm 164.1 (C5), 153.2 (C16), 149.3 (C4), 142.6 (C12), 140.1 (C1), 132.0 (C10), 129.4 (C3), 129.3 (C6), 129.1 (C7), 128.5 (C14), 125.4 (C13), 123.7 (C8), 123.5 (C2), 123.4 (C15), 123.4 (C11), 123.1 (C9), 79.7 (C17), 28.0 (C18).

HRMS (ESI) m/z: $[M+H]^+$ calculated for $C_{22}H_{22}N_3O_5S$: 440.1280, found 440.1280.

Tert-butyl (2-(4-aminobenzamido)-4-(thiophen-2-yl)phenyl)carbamate (11)

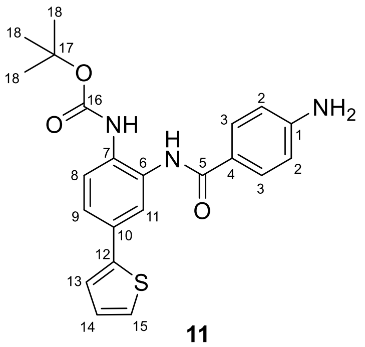

11

10 (0.33 g, 0.76 mmol) and $SnCl_2$ (0.86 g, 4.55 mmol) in MeOH/DCM (1:1, 120 mL) were stirred at room temperature for 1 week. The reaction mixture was then cooled to 0 °C, and saturated Na_2CO_3 (40 mL) was added slowly. The product was extracted into DCM (4 x 40 mL), the organic layers were combined and washed with saturated NaCl (2 x 80 mL). The organic layer was dried over Na_2SO_4, filtered and concentrated *in vacuo* to afford **11** as a yellow crystalline solid (0.28 g, 0.62 mmol, 82%).

^{1}H NMR (400 MHz, DMSO-d_6) δH ppm 9.59 (s, 1H, NH), 8.72 (br s, 1H, NH), 7.82 (d, J = 2.1 Hz, 1H, H11), 7.71 (d, J = 8.4 Hz, 2H, H3), 7.55 (d, J = 8.5 Hz, 1H, H8), 7.52 (dd, J = 5.0, 1.0 Hz, 1H, H13), 7.47 (dd, J = 8.5, 2.1 Hz, 1H, H9), 7.44 (dd, J = 3.6, 1.0 Hz, 1H, H15), 7.13 (dd, J = 5.0, 3.6 Hz, 1H, H14), 6.62 (d, J = 8.4 Hz, 2H, H2), 5.85 (br s, 2H, NH_2), 1.47 (s, 9H, H18).

^{13}C NMR (101 MHz, DMSO- d_6) δC ppm 165.4 (C5), 153.4 (C16), 152.6 (C4), 142.8 (C12), 130.8 (C6), 130.7 (C10), 129.7 (C7), 129.4 (C3), 128.5 (C14), 125.4 (C13), 124.3 (C8), 123.4 (C15), 122.4 (C11), 122.0 (C9), 120.0 (C1), 112.6 (C2), 79.8 (C17), 28.0 (C18).

HRMS (ESI) m/z: [M+H]+ calculated for $C_{22}H_{24}N_3O_3S$: 410.1538, found 410.1530.

Tert-butyl (2-(4-acetamidobenzamido)-4-(thiophen-2-yl)phenyl)carbamate

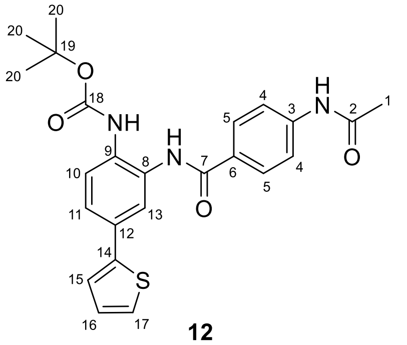

12

NEt_3 (0.1 mL, 0.71 mmol) was added to a solution of **11** (0.1 g, 0.24 mmol)) in dry THF (5 mL) at 0 °C, followed by the dropwise addition of AcCl (0.02 mL, 0.28 mmol). The mixture was stirred at 0 °C for 30 minutes, then at room temperature for 2 hours. The reaction mixture was concentrated *in vacuo* to afford a green solid (0.18 g). The crude product was purified by column chromatography (0-100% EtOAc) to afford **12** (0.07 g, 0.16 mmol, 68% yield) as a pale yellow/green solid.

^{1}H NMR (400 MHz, DMSO-d_6) δH ppm 10.25 (s, 1H, NH), 9.83 (s, 1H, NH), 8.73 (br s, 1H, NH) 7.94 (d, J = 8.7 Hz, 2H, H5), 7.82 (d, J = 2.0 Hz, 1H, H13), 7.74 (d, J = 8.7 Hz, 2H, H4), 7.60 (d, J = 8.5 Hz, 1H, H10), 7.48-7.54 (m, 2H, H11, H17), 7.45 (d, J = 3.7 Hz, 1H, H15), 7.13 (dd, J = 4.9, 3.7 Hz, 1H, H16), 2.10 (s, 3H, H1), 1.46 (s, 9H, H20).

^{13}C NMR (101 MHz, DMSO-d_6) δC ppm 168.8 (C2), 165.0 (C7), 153.3 (C18), 142.7 (C14), 142.6 (C3), 131.1 (C9), 130.1 (C8), 129.6 (12), 128.6 (C5), 128.5 (C16), 128.2 (C6), 125.4 (C17), 124.2 (C10), 123.4 (C15), 122.8 (C13), 122.5 (C11), 118.1 (C4), 79.8 (C19), 28.0 (C20), 24.1 (C1).

HRMS (ESI) m/z: $[M+H]^+$ calculated for $C_{24}H_{26}N_3O_4S$: 452.1644, found 452.1643.

4-acetamido-N-(2-amino-5-(thiophen-2-yl)phenyl)benzamide

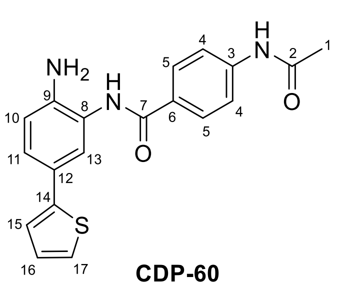

CDP-60

TFA (0.16 mL) was added to a stirring solution of **12** (0.05 g, 0.12 mmol) in DCM (1 mL) and the resulting reaction mixture was stirred at room temperature for 4 hours. The reaction mixture was concentrated *in vacuo* to afford a brown oil (0.06 g). The crude oil was dissolved in MeOH (1 mL), agitated in MP-carbonate resin (3.02 mmol/g loading capacity, 0.17 g) for 2.5 hours and then filtered.

The filtrate was concentrated *in vacuo* to afford **CPD-60** (0.04 g, 0.1 mmol, 93% yield) as a pale yellow/green solid.

^{1}H NMR (400 MHz, DMSO-d_6) δ_H ppm 10.21 (s, 1H, NH), 9.63 (s, 1H, NH), 7.96 (d, J = 8.7 Hz, 2H, H5), 7.71 (d, J = 8.7 Hz, 2H, H4), 7.47 (d, J = 2.2 Hz, 1H, H13), 7.35 (dd, J = 5.1, 1.0 Hz, 1H, H17), 7.29 (dd, J = 8.3, 2.2 Hz, 1H, H11), 7.24 (dd, J = 3.6, 1.0 Hz, 1H, H15), 7.05 (dd, J = 5.1, 3.6 Hz, 1H, H16), 6.81 (d, J = 8.3 Hz, 1H, H10), 5.13 (s, 2H, NH_2), 2.09 (s, 3H, H1).

^{13}C NMR (101 MHz, DMSO-d_6) δ_C ppm 168.8 (C2), 164.9 (C7), 144.3 (C14), 143.0 (C9), 142.2 (C3), 128.7 (C5) 128.6 (C6), 128.2 (C16), 123.9 (C13), 123.8 (C11), 123.6 (C8), 123.2 (C17), 122.3 (C12), 121.0 (C15), 118.0 (C4), 116.4 (C10), 24.1 (C1).

HRMS (ESI) m/z: $[M+H]^+$ calculated for $C_{19}H_{18}N_3O_2S$: 352.1120, found 352.1120.

2.3 Compound 1

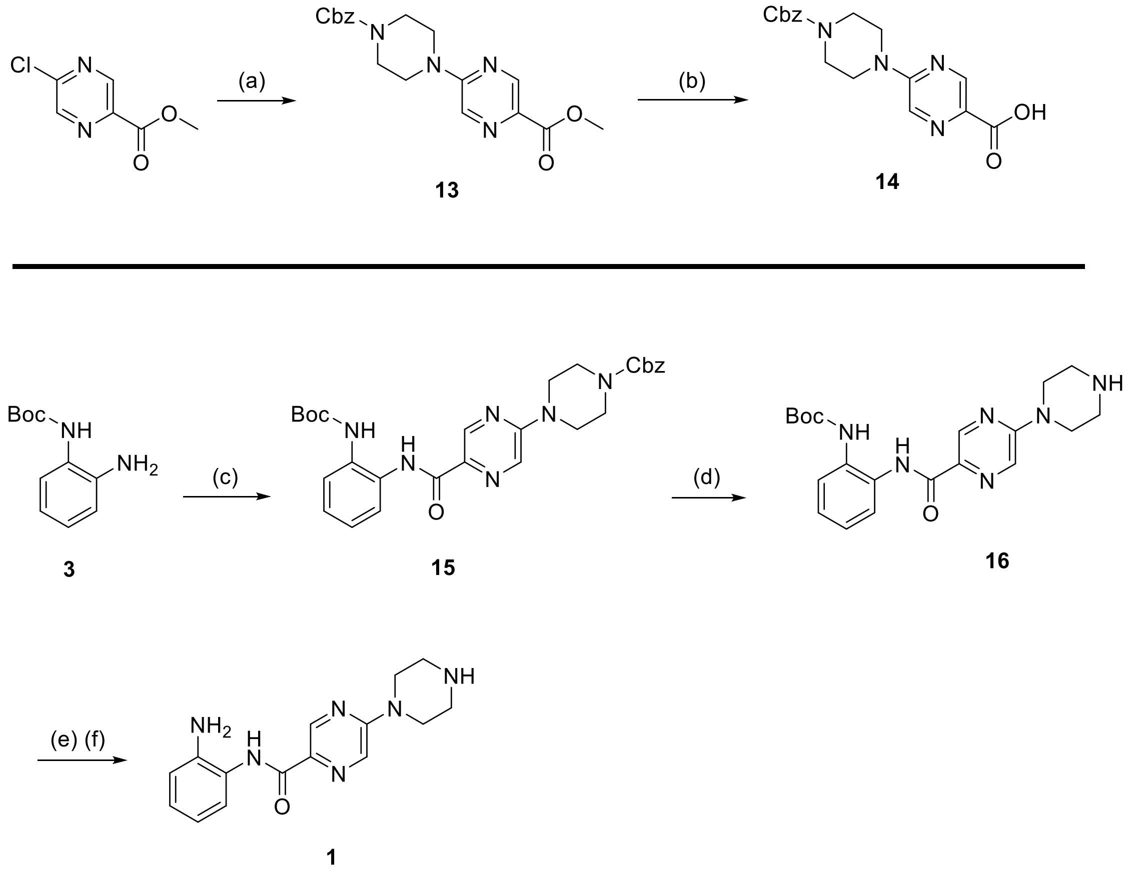

Scheme 3 – Synthesis of **1**. Reagents and conditions: (a) (a) Cbz-piperazine, DIPEA, 1,4-dioxane, 20 h, 110 °C, 94 %; (b) 2M NaOH, MeOH:THF (1:1), 48 h, rt, 87 %; (c) **14**, HATU, DIPEA, rt, 48 h, 75%; (d) H_2, 10% Pd/C, rt, 48 h, 85%; (e) TFA, DCM, rt, 24 h; (f) MP-carbonate resin, MeOH, rt, 4 h, 85%.

Methyl 5-(4-((benzyloxy)carbonyl)piperazin-1-yl)pyrazine-2-carboxylate (13)

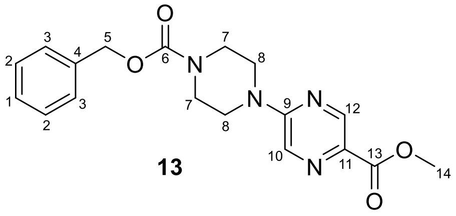

Methyl 5-chloropyrazine-2-carboxylate (2.97 g, 17.2 mmol), Cbz-piperizine (7.54 g, 34.2 mmol) and DIPEA (6 mL, 34.3 mmol) in 1,4-dioxane (30 mL) were heated at 110 ˚C for 20 hours. The reaction mixture was then concentrated *in vacuo* to yield an orange/brown solid, which was re-dissolved in DCM (120 mL) and washed with H_2O (100 mL) and sat. NaCl (100 mL). The organic layer was collected, dried over Na_2SO_4, filtered and the solvent removed *in vacuo* to afford a crude red/brown solid (7.27 g, 20.4 mmol). The solid was washed under vacuum with diethyl ether to afford **13** as an orange/brown solid (5.74 g, 16.1 mmol, 94%).

^{1}H NMR (400 MHz, DMSO-d_6) δH ppm 8.67 (d, J = 1.3 Hz, 1H, H12), 8.37 (d, J = 1.3 Hz, 1H, H10), 7.41-7.35 (m, 5H, H1, H2, H3), 5.13 (s, 2H, H5), 3.82 (s, 3H, H14), 3.78-3.76 (m, 4H, H8), 3.54-3.52 (m, 4H, H7).

^{13}C NMR (100 MHz, DMSO-d_6) δC ppm 165.0 (C13), 155.0 (C9), 154.95 (C6), 144.9 (C12), 137.2 (C4), 131.0 (C10), 130.5 (C11), 128.9 (C2/3), 128.4 (C1), 128.1 (C2/3), 66.9 (C5), 52.2 (C14), 43.8 (C8), 43.3 (C7).

HRMS (ESI) m/z: $[M+H]^+$ calculated for $C_{18}H_{21}N_4O_4$: 357.1563, found 357.1563.

5-(4-((benzyloxy)carbonyl)piperazin-1-yl)pyrazine-2-carboxylic acid (14)

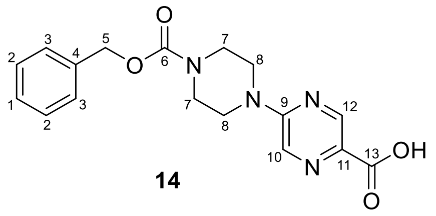

13 (3.89 g, 10.9 mmol) was dissolved in MeOH:THF solution (1:2, 150 mL) and 2M NaOH (aqueous, 16.5 mL, 33.3 mmol) was added to the reaction flask. The resulting mixture was stirred vigorously at room temperature for 48 hours. The reaction mixture was then concentrated *in vacuo* to yield a pale-yellow suspension, which was re-suspended in EtOAc (300 mL) and washed with sat. $KHSO_4$ (100 mL) and sat. NaCl (2 x 50 mL). The organic layer was collected, dried over Na_2SO_4, filtered and the solvent removed *in vacuo* to afford **14** as a white/pale-yellow solid (3.24 g, 9.5 mmol, 87%).

^{1}**H NMR** (400 MHz, DMSO-d$_6$) δH ppm 12.74 (br s, 1H, OH), 8.66 (d, *J* = 1.3 Hz, 1H, H12), 8.36 (d, *J* = 1.3 Hz, 1H, H10), 7.41-7.33 (m, 5H, H1, H2, H3), 5.13 (s, 2H, H5), 3.78-3.76 (m, 4H, H8), 3.55-3.49 (m, 4H, H7).

^{13}**C NMR** (100 MHz, DMSO-d$_6$) δC ppm 166.0 (C13), 155.0 (C9), 154.96 (C6), 144.8 (C12), 137.3 (C4), 131.4 (C10), 130.8 (C11), 128.9 (C2/3), 128.4 (C1), 128.1 (C2/3), 66.9 (C5), 43.9 (C8), 43.3 (C7).

HRMS (ESI) m/z: $[M+H]^+$ calculated for $C_{17}H_{19}N_4O_4$: 343.1406, found 343.1406.

Benzyl 4-(5-((2-((tert-butoxycarbonyl)amino)phenyl)carbamoyl)pyrazin-2-yl) piperazine-1-carboxylate (15)

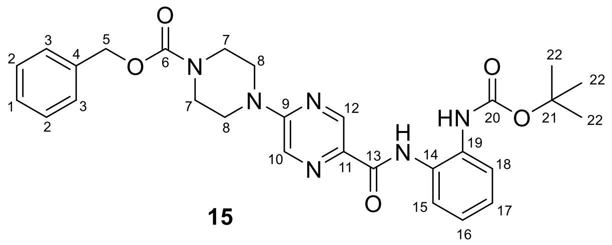

15

DIPEA (0.2 mL, 2.1 mmol) and HATU (0.71 g, 1.8 mmol) were added to a solution of **14** (0.59 g, 1.7 mmol) in anhydrous DMF (10 mL) and stirred at room temperature for 15 minutes. **3** (0.3 g, 1.4 mmol), dissolved in anhydrous DMF (3 mL), was then added dropwise, and the reaction mixture was stirred at room temperature for 48 hours. The reaction mixture was then diluted in EtOAc (100 mL) and washed with saturated $NaHCO_3$ (2 x 50 mL) and H_2O (1 x 50 mL). The product precipitated in the organic layer and was then filtered under vacuum to afford **15** as a yellow solid (0.58 g, 1.1 mmol, 75%).

^{1}**H NMR** (400 MHz, DMSO- *d*$_6$) δ 10.00 (s, 1H, NH), 9.03 (s, 1H, NH), 8.75 (d, *J* = 1.3 Hz, 1H, H12), 8.25 (d, *J* = 0.6 Hz, 1H, H10), 7.96 (d, *J* = 7.4 Hz, 1H, H15), 7.41 – 7.31 (m, 5H, H1, H2, H3), 7.28 – 7.19 (m, 2H, H17, H18), 7.13 (td, *J* = 7.6, 1.5 Hz, 1H, H16), 5.13 (s, 2H. H5), 3.82 – 3.75 (m, 4H, H7), 3.56 (s, 4H, H8), 1.48 (s, 9H, H22).

^{13}**C NMR** (101 MHz, DMSO-*d*$_6$) δ 161.7 (C6), 155.1 (C13), 154.5 (C9), 153.9 (C20), 142.2 (C12), 136.8 (C4), 132.3 (C11), 132.1 (C19), 129.6 (C14), 128.8 (C10), 128.5 (C3), 127.9 (C1), 127.6 (C2), 125.6 (C17), 125.5 (C18), 124.5 (C16), 123.6 (C15), 79.7 (C21), 66.4 (C5), 43.5 (C7), 42.8 (C8), 28.1 (C22).

HRMS (ESI) m/z: $[M+H]^+$ calculated for $C_{28}H_{33}N_6O_5$: 533.2512, found 533.2512.

Tert-butyl (2-(5-(piperazin-1-yl)pyrazine-2-carboxamido)phenyl)carbamate (16)

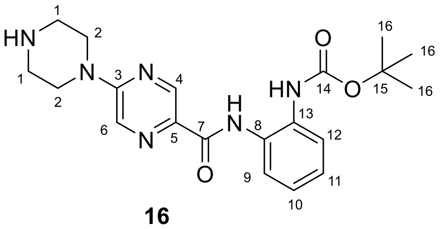

16

To a solution of **15** (0.08 g, 0.14 mmol) in MeOH (15 mL), 10% Pd/C (0.008 g) was added. The reaction flask was filled with nitrogen and evacuated 3 times using a Schlenk line, before a balloon of hydrogen was added and the resultant mixture stirred vigorously for 48 hours. The reaction mixture was filtered through celite, and the solvent was removed *in vacuo* to afford **16** as a white solid (0.05 g, 0.12 mmol, 85%).

^{1}H NMR (400 MHz, DMSO-d_6) δ 9.97 (s, 1H, NH), 9.02 (s, 1H, NH), 8.71 (d, J = 1.3 Hz, 1H, H4), 8.21 (d, J = 0.9 Hz, 1H, H6), 7.96 (d, J = 7.9 Hz, 1H, H9), 7.27 – 7.19 (m, 2H, H11, H12), 7.12 (td, J = 7.6, 1.5 Hz, 1H, H10), 3.65 (m, 4H, H2), 2.79 (m, 4H, H1), 1.48 (s, 9H, H16).

^{13}C NMR (101 MHz, DMSO-d_6) δ 161.8 (C7), 155.3 (C14), 154.0 (C3), 142.3 (C4), 132.2 (C5), 131.7 (C8), 129.5 (C13), 128.6 (C6), 125.7 (C12), 125.5 (C11), 124.5 (C10), 123.6 (C9), 79.7 (C15), 45.4 (C1), 45.2 (C2), 28.1 (C16).

HRMS (ESI) m/z: $[M+H]^+$ calculated for $C_{20}H_{26}N_6O_3$: 399.2145, found 399.2143.

N-(2-aminophenyl)-5-(piperazin-1-yl)pyrazine-2-carboxamide (1)

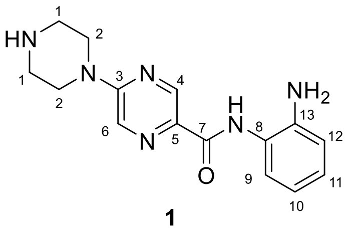

1

16 (0.05 g, 0.13 mmol) was dissolved in DCM (7 mL) and cooled to 0 °C. TFA (0.1 mL) was then added and the reaction was stirred at room temperature for 24 hours. The reaction mixture was concentrated *in vacuo* and the compound was re-dissolved in MeOH (20 mL) and agitated in MP-carbonate resin (3.02 mmol/g loading capacity, 0.25 g) for 4 hours. The reaction mixture was then filtered and the filtrate was concentrated *in vacuo*. The crude product was purified by column chromatography (alumina, 5% MeOH in DCM) to afford **1** as a crystalline white solid (0.03 g, 0.11 mmol, 85%).

^{1}**H NMR** (400 MHz, CD_3OD) δ 8.77 (d, *J* = 1.3 Hz, 1H, H4), 8.24 (d, *J* = 1.4 Hz, 1H, H6), 7.39 (dd, *J* = 7.9, 1.4 Hz, 1H, H9), 7.05 (ddd, *J* = 7.9, 7.4, 1.5 Hz, 1H, H11), 6.91 (dd, *J* = 8.0, 1.4 Hz, 1H, H12), 6.79 (td, *J* = 7.8, 1.4 Hz, 1H, H10), 3.75 (m, 4H, H2), 2.94 (m, 4H, H1).

^{13}**C NMR** (101 MHz, CD_3OD) δ 164.9 (C7), 156.9 (C3), 143.5 (C4), 142.9 (C8), 133.7 (C5), 130.0 (C6), 127.9 (C13), 126.5 (C11), 125.4 (C9), 119.8 (C12), 118.7 (C10), 46.2 (C1), 46.0 (C2).

HRMS (ESI) m/z: $[M+H]^+$ calculated for $C_{15}H_{19}N_6O$: 299.1621, found 299.1620.

Spectroscopic data consistent with literature.[2]

2.4 Compound 2

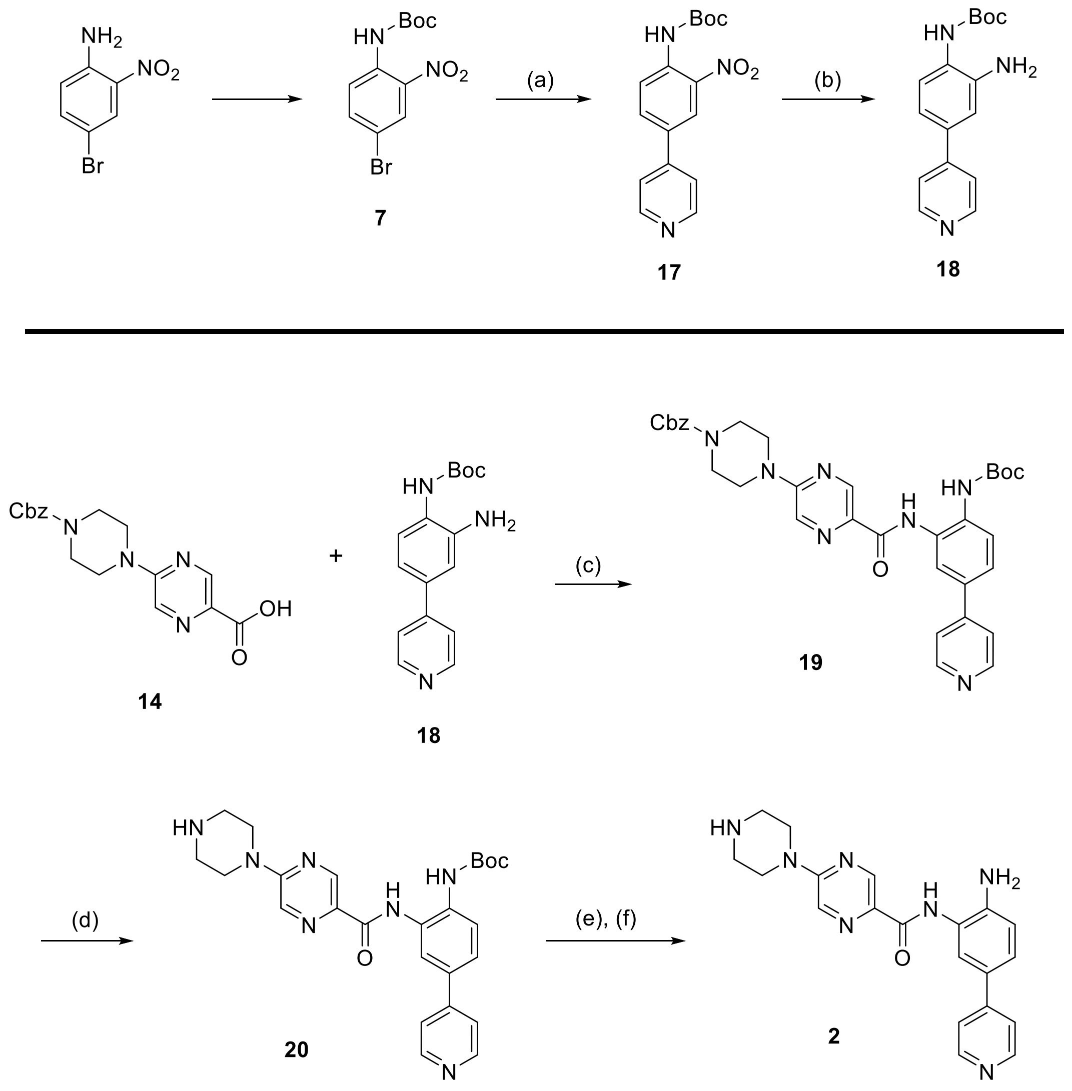

Scheme 4 - Synthesis of **2**. Reagents and conditions: (a) 4-pyridinylboronic acid, Cs_2CO_3, $Pd(PPh_3)_4$, 1,4-dioxane, 110 °C, 23 h, 79 %; (b) 10% Pd/C, H_2, EtOH, rt, 24 h, 97 %; (c) HATU, DIPEA, 0 °C to rt, 17 h, 58 %; (d) 10% Pd/C, H_2, EtOH, rt, 36 h, 86 %; (e) TFA, DCM, 0 °C to rt, 18 h; (f) MP-carbonate resin, MeOH, rt, 3 h, 94%.

***Tert*-butyl (2-nitro-4-(pyridin-4-yl)phenyl)carbamate (17)**

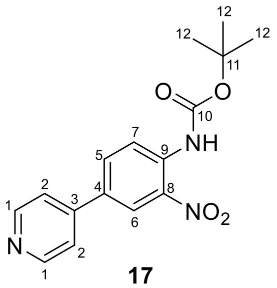

17

7 (0.89 g, 2.8 mmol), pyridin-4-yl-boronic acid (0.47 g, 3.6 mmol), Cs_2CO_3 (2.64 g, 8.4 mmol, aqueous) and $Pd(PPh_3)_4$ (0.16 g, 0.14 mmol) were dissolved in dry 1,4-dioxane (20 mL) and heated at 110 ˚C for 22 hours. The reaction mixture was then concentrated *in vacuo* to yield a dark red oil. The oil was re-dissolved in EtOAc (100 mL) and the organic layer was washed with H_2O (100 mL) and saturated NaCl (3 x 100 mL). The organic layer was collected, dried over Na_2SO_4, filtered and the solvent evaporated *in vacuo* to yield **17** as a crude brown/dark red oil (1.03 g, 3.3 mmol). The crude product was then purified by flash column chromatography (50% EtOAc in hexane) to afford **17** as a yellow solid (0.7 g, 2.2 mmol, 79%).

^{1}H NMR (400 MHz, $CDCl_3$) δH ppm 9.75 (br s, 1H, NH), 8.75-8.70 (m, 3H, H1, H7), 8.50 (d, *J* = 2.2 Hz, 1H, H6), 7.90 (dd, *J* = 8.8 Hz, 2.1 Hz, 1H, H5), 7.53-7.51 (m, 2H, H2), 1.57 (s, 9H, H12).

^{13}C NMR (100 MHz, $CDCl_3$) δC ppm 152.0 (C10), 150.7 (C1), 145.3 (C3), 136.5 (C9), 136.1 (C4), 133.8 (C5), 131.7 (C8), 124.0 (C6), 121.5 (C7), 121.0 (C2), 82.3 (C11), 28.2 (C12).

HRMS (ESI) m/z: $[M+H]^+$ calculated for $C_{16}H_{18}N_3O_4$: 316.1297, found 316.1298.

***Tert*-butyl (2-amino-4-(pyridin-4-yl)phenyl)carbamate (18)**

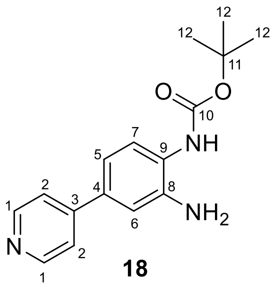

18

17 (0.47 g, 1.5 mmol) was dissolved in EtOH (70 mL) and 10% Pd/C (0.051 g) was added. The reaction flask was evacuated and filled with nitrogen three times using a Schlenk line. Next, a balloon of hydrogen was added, and the reaction mixture was stirred vigorously for 24 hours. The balloon of hydrogen was removed, and the flask was evacuated and filled with nitrogen. The reaction mixture was filtered through celite and then the celite was washed further with EtOH (3 x 50 mL). The filtrate was concentrated *in vacuo* to yield **18** as a white solid (0.41 g, 1.4 mmol, 97%).

^{1}H NMR (400 MHz, $CDCl_3$) δH ppm 8.63- 8.61 (m, 2H, H1), 7.47-7.43 (m, 3H, H2, H7), 7.09 (dd, *J* = 8.2 Hz, 2.0 Hz, 1H, H5), 7.04 (d, *J* = 2.0 Hz, 1H, H6), 6.33 (br s, 1H, NH), 3.85 (br s, 2H, NH_2), 1.53 (s, 9H, H12).

^{13}C NMR (100 MHz, $CDCl_3$) δC ppm 153.6 (C10), 150.2 (C1), 148.0 (C3), 139.8 (C8), 135.6 (C4), 126.1 (C9), 124.6 (C7), 121.4 (C2), 118.5 (C5), 116.0 (C6), 81.0 (C11), 28.3 (C12).

HRMS (ESI) m/z: $[M+H]^+$ calculated for $C_{16}H_{20}N_3O_2$: 286.1555, found 286.1555.

Benzyl 4-(5-((2-((tert-butoxycarbonyl)amino)-5-(pyridin-4-yl)phenyl)carbamoyl) pyrazin-2-yl)piperazine-1-carboxylate (19)

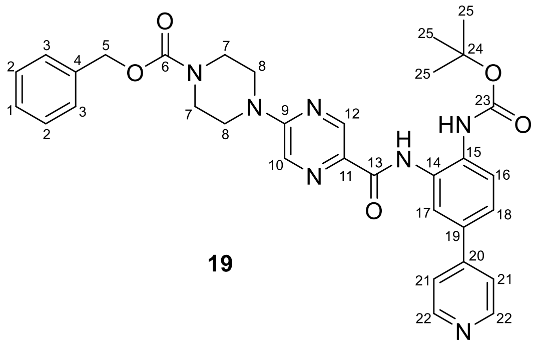

19

Solution of **14** (0.68 g, 2.0 mmol) in dry DMF (20 mL) was cooled down to 0 ˚C and HATU (0.88 g, 2.3 mmol) and DIPEA (1.5 mL, 7.5 mmol) were added. The reaction mixture was stirred at 0 ˚C for 40 minutes and then **18** (0.43 g, 1.5 mmol) in dry DMF (15 mL) was added. The resultant mixture was stirred at room temperature for 18 hours. The reaction mixture was then diluted with EtOAc (100 mL) and washed with saturated $NaHCO_3$ (2 x 50 mL) and saturated NaCl solution (2 x 50 mL). The organic layer was dried over Na_2SO_4, filtered and the solvent was evaporated *in vacuo* to yield crude brown solid (1.38 g, 2.3 mmol). The crude solid was washed under vacuum with EtOAc to afford **19** as a white solid (0.61 g, 1.0 mmol, 58%).

^{1}H NMR (400 MHz, DMSO-d_6) δH ppm 10.14 (s, 1H, NH), 9.21 (br s, 1H, NH), 8.78 (d, *J* = 1.3 Hz, 1H, H12), 8.64 (d, *J* = 5.0 Hz, 2H, H22), 8.36 (d, *J* = 2.0 Hz, 1H, H17), 8.29 (d, *J* = 1.0 Hz, 1H, H10), 7.69 (d, *J* = 5.1 Hz, 2H, H21), 7.61 (dd, *J* = 8.4 Hz, 2.2 Hz, 1H, H18), 7.46 (d, *J* = 8.4 Hz, 1H, H16), 7.41-7.32 (m, 5H, H1, H2, H3), 5.14 (s, 2H, H5), 3.85-3.77 (m, 4H, H8), 3.63-3.52 (m, 4H, H7) , 1.51 (s, 9H, H25).

^{13}C NMR (100 MHz, DMSO-d_6) δC ppm 162.5 (C13), 155.6 (C23), 155.0 (C9), 154.2 (C6), 150.8 (C22), 146.8 (C20), 142.8 (C12), 137.3 (C4), 134.3 (C19), 132.7 (C11), 132.4 (C14), 131.5 (C15), 129.3 (C10), 128.9 (C2/3), 128.4 (C1), 128.1 (C2/3), 126.2 (C16), 123.6 (C18), 122.9 (C17), 121.4 (C21), 80.5 (C24), 66.9 (C5), 44.0 (C8), 43.3 (C7), 28.5 (C25).

HRMS (ESI) m/z: $[M+H]^+$ calculated for $C_{33}H_{37}N_7O_5$: 610.2778, found 610.2784.

Tert-butyl (2-(5-(piperazin-1-yl)pyrazine-2-carboxamido)-4-(pyridin-4-yl) phenyl) carbamate (20)

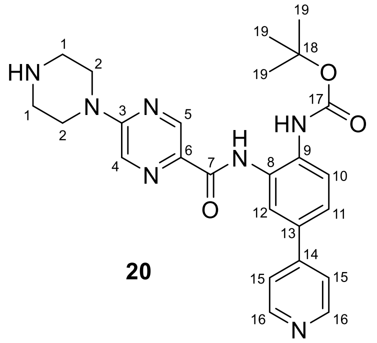

20

19 (0.36 g, 0.6 mmol) was dissolved in EtOH (90 mL) and 10% Pd/C (0.049 g) was added. The reaction flask was evacuated and filled with nitrogen three times using a Schlenk line. Next, a balloon of hydrogen was added, and the reaction mixture was stirred vigorously for 36 hours at room temperature. The balloon of hydrogen was removed, and the flask was evacuated and filled with nitrogen. The reaction mixture was filtered through celite and then the celite was washed further with EtOH (4 x 50 mL). The filtrate was concentrated *in vacuo* to yield **20** as a crude pale-yellow fluffy solid (0.35 g, 0.7 mmol). The crude product was purified by flash column chromatography (alumina, 3-5% MeOH in DCM) to afford **20** as a fluffy pale-yellow solid (0.24 g, 0.5 mmol, 86%).

^{1}H NMR (400 MHz, $CDCl_3$) δH ppm 9.65 (s, 1H, NH), 8.96 (d, J = 1.3 Hz, 1H, H5), 8.64 (br s, 2H, H16), 8.00 (d, J = 1.3 Hz, 1H, H4), 7.95 (br s, 1H, NH), 7.77 (d, J = 8.5 Hz, 2H, H15), 7.51-7.47 (m, 3H, H10, H11, H12), 3.74 (t, J = 5.0 Hz, 4H, H2), 3.02 (t, J = 4.8 Hz, 4H, H1), 1.72 (br s, 1H, NH), 1.53 (s, 9H, H19).

^{13}C NMR (100 MHz, $CDCl_3$) δC ppm 163.2 (C7), 155.4 (C17), 154.5 (C3), 149.2 (C16), 148.1 (C14), 142.5 (C5), 134.2 (C13), 132.1 (C6), 131.7 (C8), 131.4 (C9), 128.3 (C4), 125.3 (C11), 123.4 (C12), 122.4 (C15), 121.4 (C10), 80.5 (C18), 44.6 (C1), 44.3 (C2), 27.4 (C19).

HRMS (ESI) m/z: $[M+H]^+$ calculated for $C_{25}H_{30}N_7O_3$: 476.2410, found 476.2403.

N-(2-amino-5-(pyridin-4-yl)phenyl)-5-(piperazin-1-yl)pyrazine-2-carboxamide (2)

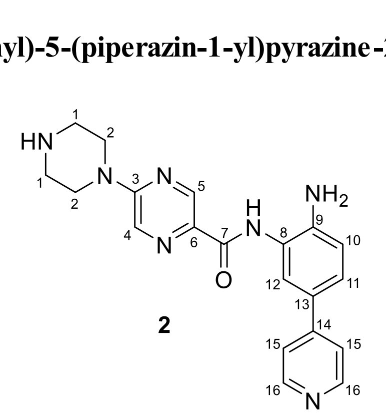

2

20 (0.021 g, 0.04 mmol) was dissolved in dry DCM (3 mL) and the reaction flask was cooled to 0 °C. TFA (0.5 mL, 6.5 mmol) was then added and the resulting reaction mixture was stirred at room temperature for 17 hours. The reaction mixture was then concentrated *in vacuo* to afford **2** as a green oil. The resulting oil was then re-dissolved in MeOH (5 mL) and agitated on MP-carbonate resin (3.02 mmol/g loading capacity. 0.2 g) for 2.5 hours. The resin was then filtered off and washed with MeOH (5 x 10 mL). The filtrate was concentrated *in vacuo* to afford crude **2** as a yellow solid (0.02 g, 0.04 mmol). The crude compound was purified using flash column chromatography (alumina, 5-7% MeOH in DCM) to afford pure **2** as a pale yellow solid (0.02 g, 0.04 mmol, 94%).

^{1}H NMR (400 MHz, MeOD-d_4) δH ppm 8.79 (d, *J* = 1.4 Hz, 1H, H5), 8.47-8.45 (m, 2H, H16), 8.26 (d, *J* = 1.3 Hz, 1H, H4), 7.86 (d, *J* = 2.1 Hz, 1H, H12), 7.67-7.63 (m, 2H, H15), 7.51 (dd, *J* = 8.4 Hz, 2.2 Hz, 1H, H11), 6.99 (d, *J* = 8.4 Hz, 1H, H10), 3.80-3.77 (m, 4H, H2), 2.99-2.96 (m, 4H, H1).

^{13}C NMR (100 MHz, MeOD-d_4) δC ppm 163.8 (C7), 155.5 (C3), 148.94 (C16), 148.88 (C9), 143.8 (C14), 142.3 (C5), 132.4 (C6), 128.7 (C4), 126.4 (C13), 125.2 (C11), 124.0 (C12), 123.7 (C8), 120.5 (C15), 117.1 (C10), 44.6 (C1), 44.3 (C2).

HRMS (ESI) m/z: $[M+H]^+$ calculated for $C_{20}H_{22}N_7O$: 376.1886, found 376.1886.

3. Synthesis of PROTACs

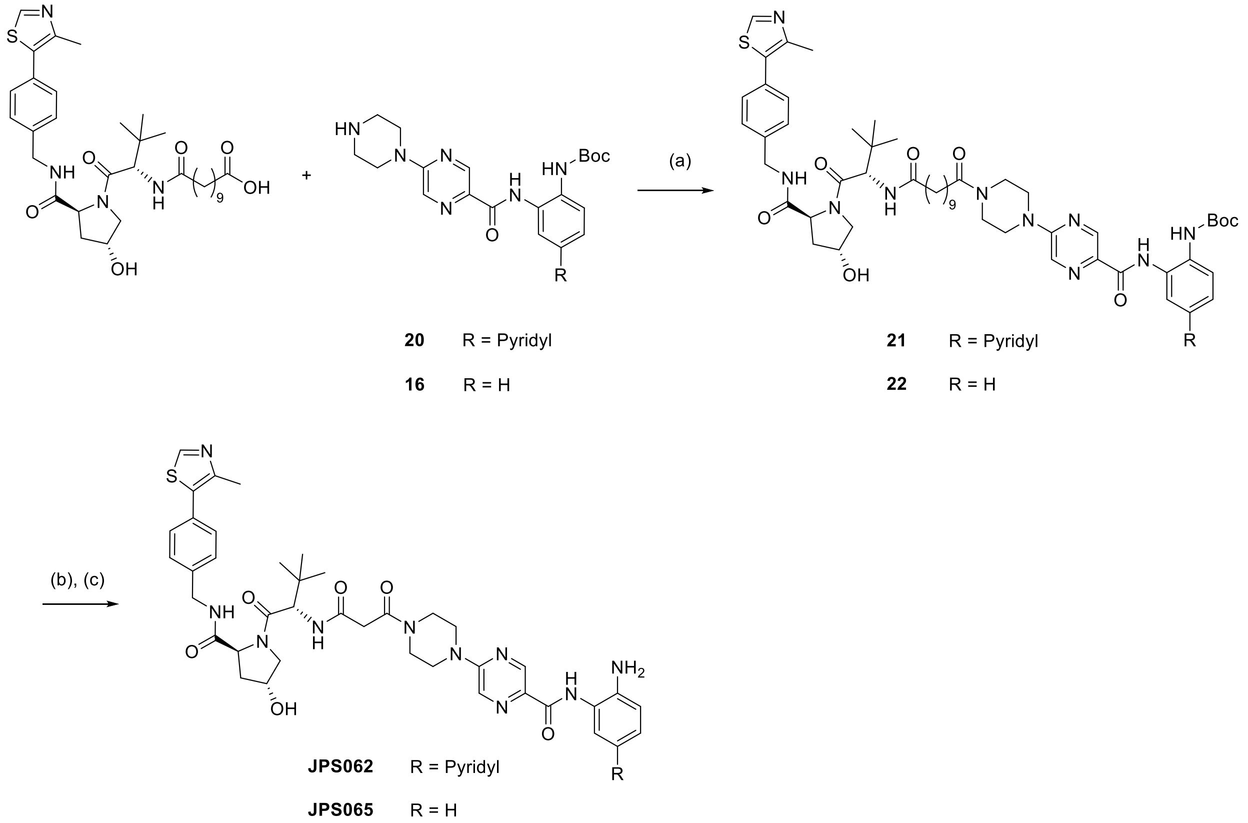

Scheme 5. Synthesis of **JPS062** and **JPS065**. Reagents and conditions: (a) HATU, DIPEA, DMF, r.t., overnight, 76-82%; (b) TFA, DCM, r.t., 3 h; (c) MP-carbonate resin, MeOH, r.t., 2 h, 83-90%

Tert-butyl(2-(5-(4-(11-(((S)-1-((2S,4R)-4-hydroxy-2-((4-(4-methylthiazol-5-yl)benzyl)carbamoyl) pyrrolidin-1-yl)-3,3-dimethyl-1-oxobutan-2-yl)amino)-11-oxoundecanoyl)piperazin-1-yl)pyrazine-2-carboxamido)-4-(pyridin-4-yl)phenyl)carbamate (21)

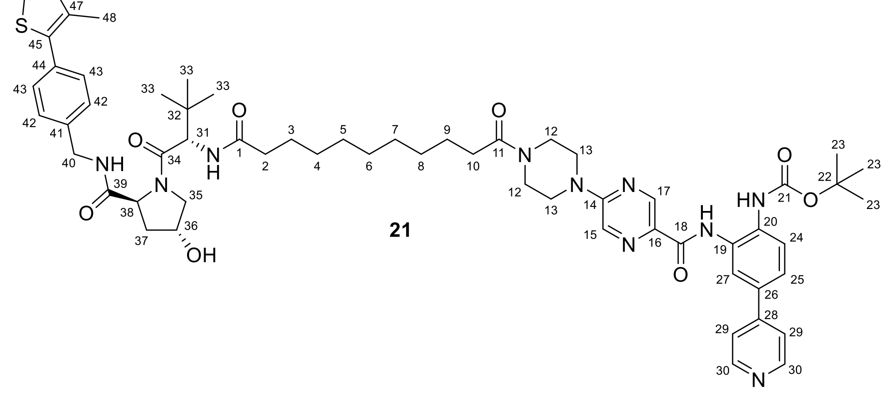

To a solution of VH 032 amide-alkylC9-acid (0.02 g, 0.039 mmol) in dry DMF (2 mL) at 0 °C, DIPEA (20.5 µL, 0.118 mmol) and HATU (0.02 g, 0.051 mmol) were added. The reaction mixture was stirred for 15 minutes, after which a solution of **20** (0.03 g, 0.039 mmol) in DMF (1 mL) was added slowly and the resultant solution stirred at room temperature for 16 hours. The reaction mixture was diluted in EtOAc (20 mL), then washed with saturated $NaHCO_3$ (2 x 10 mL) and brine (2 x 10 mL). The organic layer was dried over $MgSO_4$, filtered and concentrated *in vacuo* to afford a yellow tar. The crude product was purified by column chromatography (1-8% MeOH in DCM, alumina (basic)) to afford **21** (0.04 g, 0.032 mmol, 82% yield) as a pale-yellow solid.

^{1}H NMR (400 MHz, CD_3OD) δ_H ppm 8.86 (s, 1H, H46), 8.81 (d, *J* = 1.2 Hz, 1H, H17), 8.54 - 8.60 (m, 2H, H30), 8.34 (d, *J* = 2.0 Hz, 1H, H27), 8.19 (d, *J* = 1.2 Hz, 1H, H15), 7.70 - 7.75 (m, 2H, H29), 7.59 (dd, *J* = 8.3, 2.0 Hz, 1H, H25), 7.50 (d, *J* = 8.3 Hz, 1H, H24), 7.42 - 7.46 (m, 2H, H42), 7.37 - 7.42 (m, 2H, H43), 4.64 (s, 1H, H31), 4.55 - 4.60 (m, 1H, H38), 4.52 (d, *J* = 15.5 Hz, 1H, H40), 4.47 - 4.50 (m, 1H, H36), 4.34 (d, *J* = 15.5 Hz, 1H, H40), 3.88 - 3.93 (m, 1H, H35), 3.66 - 3.85 (m, 9H, H35, H12, H13), 2.46 (s, 3H, H48), 2.42 (t, *J* = 7.6 Hz, 2H, H10), 2.18 - 2.30 (m, 3H, H37, H2), 2.04 - 2.11 (m, 1H, H37), 1.57 - 1.65 (m, 4H, H3, H9), 1.54 (s, 9H, H23), 1.30 - 1.36 (m, 10H, H4, H5, H6, H7, H8), 1.03 (s, 9H, H33).

^{13}C NMR (101 MHz, CD_3OD) δ_C ppm 176.2 (C1), 174.65 (C11/39), 174.6 (C11/39), 172.5 (C34), 164.8 (C14), 157.0 (C18), 156.2 (C21), 153.0 (C46), 150.8 (C30), 149.9 (C28), 149.2 (C47), 144.0 (C17), 140.4 (C41), 136.1 (C26), 134.1 (C16), 133.5 (C45), 133.4 (C19/20), 133.0 (C19/20), 131.6 (C44), 130.5 (C43), 130.1 (C15), 129.1 (C42), 127.0 (C24), 125.2 (C25), 124.1 (C27), 123.1 (C29), 82.1 (C22), 71.2 (C36), 61.0 (C38), 59.1 (C31), 58.2 (C35), 46.2 (C12/13), 45.4 (C12/13), 45.2 (C12/13), 43.8 (C40), 42.4 (C12/13), 39.1 (C37), 36.8 (C32), 36.7 (C2), 34.2 (C10), 30.6 (alkyl CH_2), 30.5 (alkyl CH_2), 30.5 (alkyl CH_2), 30.45 (alkyl CH_2), 30.4 (alkyl CH_2), 28.8 (23), 27.2 (C33), 27.1 (C3), 26.6 (C9), 16.0 (C48).

Tert-butyl(2-(5-(4-(11-(((S)-1-((2S,4R)-4-hydroxy-2-((4-(4-methylthiazol-5-yl)benzyl)carbamoyl) pyrrolidin-1-yl)-3,3-dimethyl-1-oxobutan-2-yl)amino)-11-oxoundecanoyl)piperazin-1-yl)pyrazine-2-carboxamido)phenyl)carbamate (22)

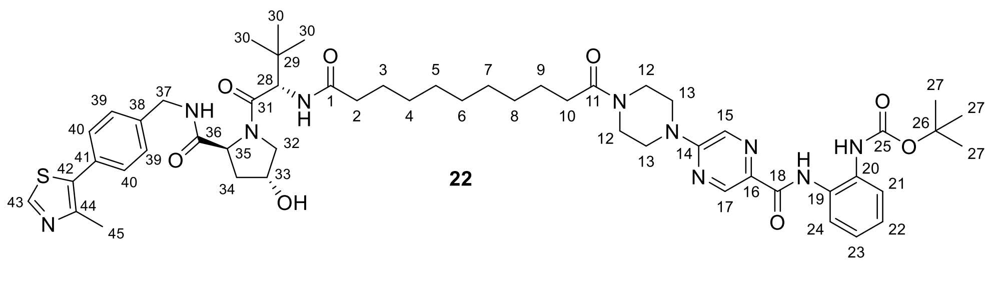

To a solution of VH 032 amide-alkylC9-acid (0.02 g, 0.050 mmol) in dry DMF (2 mL) at 0 °C, DIPEA (26.0 µL, 0.151 mmol) and HATU (0.03 g, 0.065 mmol) were added. The reaction mixture was stirred

for 15 minutes, after which a solution of **4** (0.03 g, 0.050 mmol) in DMF (2 mL) was added slowly and the resultant solution stirred at room temperature for 16 hours. The reaction mixture was diluted in EtOAc (20 mL), then washed with saturated $NaHCO_3$ (2 x 10 mL) and brine (2 x 10 mL). The organic layer was dried over $MgSO_4$, filtered and concentrated *in vacuo* to afford a yellow tar (0.07 g). The crude product was purified by column chromatography (1-8% MeOH in DCM, alumina (basic)) to afford **22** (0.04 g, 0.038 mmol, 76% yield) as a pale-yellow solid.

^{1}H NMR (400 MHz, CD_3OD) δ_H ppm 8.86 (s, 1H, H43), 8.79 (d, *J* = 1.2 Hz, 1H, H17), 8.17 (d, *J* = 1.2 Hz, 1H, H15), 7.92 (br d, *J* = 7.8 Hz, 1H, H24), 7.43 - 7.47 (m, 2H, H39), 7.38 - 7.42 (m, 2H, H40), 7.31 (dd, *J* = 7.8, 1.3 Hz, 1H, H21), 7.24 (app. td, *J* = 7.8, 1.3 Hz, 1H, H22/23), 7.18 (app. td, *J* = 7.8, 1.3 Hz, 1H, H22/23), 4.64 (s, 1H, H28), 4.55 - 4.60 (m, 1H, H35), 4.53 (d, *J* =15.5 Hz, 1H, H37), 4.47 - 4.50 (m, 1H, H33), 4.35 (d, *J* = 15.5 Hz, 1H, H37), 3.87 - 3.92 (m, 1H, H32), 3.64 - 3.85 (m, 9H, H32, H12, H13), 2.46 (s, 3H, H45), 2.43 (t, *J* = 7.6 Hz, 2H, H10), 2.18 - 2.31 (m, 3H, H34, H2), 2.04 - 2.11 (m, 1H, H34), 1.56 - 1.66 (m, 4H, H3, H9), 1.52 (s, 9H, H27), 1.30 - 1.37 (m, 10H, H4, H5, H6, H7, H8), 1.03 (s, 9H, H30).

^{13}C NMR (101 MHz, CD_3OD) δ_C ppm 176.2 (C1), 174.65 (C11/36), 174.6 (C11/36), 172.5 (C31), 164.6 (C14), 156.9 (C18), 156.5 (C25), 153.0 (C43), 149.2 (C44), 143.8 (C17), 140.4 (C38), 134.2 (C16), 133.5 (C42), 133.3 (C19/20), 131.6 (C41), 131.5 (C19/20), 130.5 (C40), 130.0 (C15), 129.1 (C39), 127.1 (C22/23), 126.8 (C21), 126.7 (C22/23), 125.5 (C24), 81.8 (C26), 71.2 (C33), 61.0 (C35), 59.1 (C28), 58.2 (C32), 46.2 (C12/13), 45.4 (C12/13), 45.2 (C12/13), 43.8 (C37), 42.3 (C12/13), 39.1 (C34), 36.8 (C29), 36.7 (C2), 34.2 (C10), 30.65 (alkyl CH_2), 30.6 (alkyl CH_2), 30.55 (alkyl CH_2), 30.5 (alkyl CH_2), 30.4 (alkyl CH_2), 28.9 (C27), 27.2 (C30), 27.1 (C3), 26.6 (C9), 16.0 (C45).

HRMS (ESI) m/z: $[M+H]^+$ calculated for $C_{53}H_{73}N_{10}O_8S$: 1009.5334, found 1009.5343.

N-(2-amino-5-(pyridin-4-yl)phenyl)-5-(4-(11-(((S)-1-((2S,4R)-4-hydroxy-2-((4-(4-methylthiazol-5-yl)benzyl)carbamoyl)pyrrolidin-1-yl)-3,3-dimethyl-1-oxobutan-2-yl)amino)-11-oxoundecanoyl)piperazin-1-yl)pyrazine-2-carboxamide (JPS062)

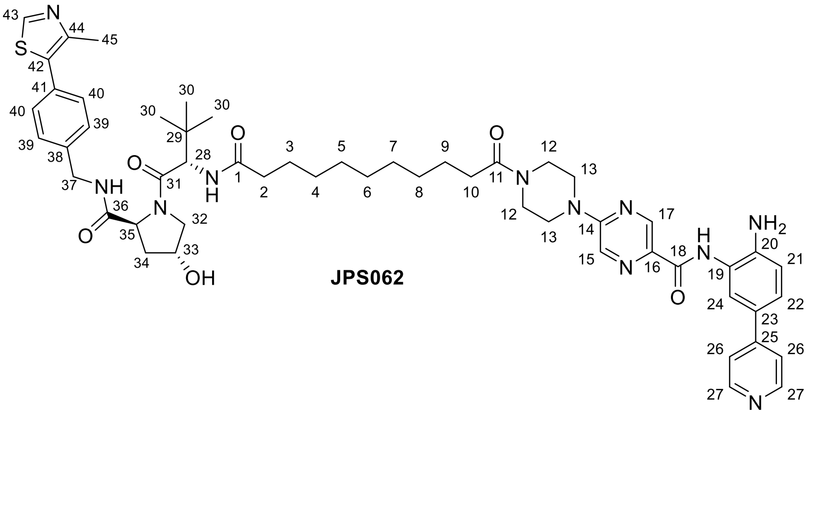

TFA (0.5 mL) was added to a stirring solution of **21** (0.04 g, 0.032 mmol) in DCM (2 mL) and the resulting reaction mixture stirred at room temperature for 4 hours. The reaction mixture was concentrated *in vacuo* to afford an orange oil (0.06 g). The crude oil was dissolved in MeOH (2 mL), agitated in MP-carbonate resin (3.02 mmol/g loading capacity, 0.22 g) for 2.5 hours and then filtered. The filtrate was concentrated *in vacuo* to afford **JPS062** (0.03 g, 0.027 mmol, 83% yield) as a pale-yellow solid. Prior to biological testing, product further purified by column chromatography (Biotage, 1-10% MeOH in DCM, alumina) and then lyophilised to afford **JPS062** (0.02 mg) as a white solid.

^{1}H NMR (400 MHz, CD_3OD) δ_H ppm 8.86 (s, 1H, H43), 8.79 (s, 1H, H17), 8.46 (d, *J* = 6.3 Hz, 2H, H27), 8.26 (s, 1H, H15), 7.86 (d, *J* = 2.0 Hz, 1H, H24), 7.64 (d, *J* = 6.3 Hz, 2H, H26), 7.50 (dd, *J* = 8.3, 2.0 Hz, 1H, H22), 7.43 - 7.47 (m, 2H, H39), 7.36 - 7.42 (m, 2H, H40), 6.99 (d, *J* = 8.3 Hz, 1H, H21), 4.64 (s, 1H, H28), 4.56 - 4.60 (m, 1H, H35), 4.52 (d, *J* = 15.5 Hz, 1H, H37), 4.47 - 4.50 (m, *J* = 1.7 Hz, 1H, H33), 4.35 (d, *J* = 15.5 Hz, 1H, H37), 3.87 - 3.94 (m, 1H, H32), 3.67 - 3.85 (m, 9H, H32, H12, H13), 2.46 (s, 3H, H45), 2.43 (t, *J* = 7.5 Hz, 2H, H10), 2.19 - 2.31 (m, 3H, H34, H2), 2.05 - 2.11 (m, 1H, H34), 1.57 - 1.64 (m, 4H, H3, H9), 1.30 - 1.35 (m, 10H, H4, H5, H6, H7, H8), 1.03 (s, 9H, H30).

^{13}C NMR (101 MHz, CD_3OD) δ_C ppm. 176.2 (C1), 174.65 (C11/36), 174.6 (C11/36), 172.5 (C31), 165.2 (C14), 156.9 (C18), 153.0 (C43), 150.45 (C27), 150.4 (C25), 149.2 (C44), 145.3 (C20), 143.8 (C17), 140.4 (C38), 134.3 (C16), 133.5 (C42), 131.6 (C41), 130.5 (C40), 130.2 (C15), 129.1 (C39), 127.9 (C23), 126.7 (C22), 125.5 (C24), 125.3 (C19), 122.1 (C26), 118.7 (C21), 71.2 (C33), 61.0 (C35), 59.1 (C28), 58.2 (C32), 46.3 (C12/13), 45.4 (C12/13), 45.2 (C12/13), 43.8 (C37), 42.4 (C12/13), 39.1 (C34), 36.8 (C29), 36.7 (C2), 34.2 (C10), 30.6 (alkyl CH_2), 30.55 (alkyl CH_2), 30.5 (alkyl CH_2), 30.45 (alkyl CH_2), 30.4 (alkyl CH_2), 27.2 (C30), 27.1 (C3), 26.6 (C9), 16.0 (C45).

HRMS (ESI) m/z: $[M+H]^+$ calculated for $C_{53}H_{68}N_{11}O_6S$: 986.5075, found 986.5074.

N-(2-aminophenyl)-5-(4-(11-(((S)-1-((2S,4R)-4-hydroxy-2-((4-(4-methylthiazol-5-yl)benzyl) carbamoyl)pyrrolidin-1-yl)-3,3-dimethyl-1-oxobutan-2-yl)amino)-11-oxoundecanoyl)piperazin-1-yl)pyrazine-2-carboxamide (JPS065)

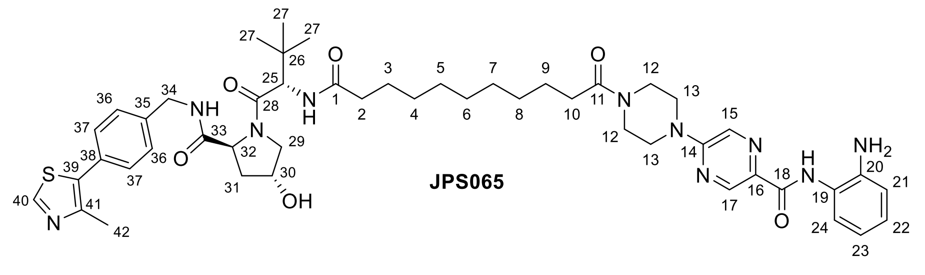

TFA (0.5 mL) was added to a stirring solution of **22** (0.04 g, 0.038 mmol) in DCM (2 mL) and the resulting reaction mixture stirred at room temperature for 4 hours. The reaction mixture was

concentrated *in vacuo* to afford an orange oil (0.07 g). The crude oil was dissolved in MeOH (2 mL), agitated in MP-carbonate resin (3.02 mmol/g loading capacity, 0.27 g) for 2.5 hours and then filtered. The filtrate was concentrated *in vacuo* to afford **JPS065** (0.03 g, 0.034 mmol, 90% yield) as a pale yellow solid. Prior to biological testing, product further purified by column chromatography (Biotage, 1-10% MeOH in DCM, alumina) and then lyophilised to afford **JPS065** (0.02 g) as a white solid.

^{1}H NMR (400 MHz, CD_3OD) δ_H ppm 8.86 (s, 1H, H40), 8.78 (d, *J* = 1.3 Hz, 1H, H17), 8.25 (d, *J* = 1.3 Hz, 1H, H15), 7.43 - 7.47 (m, 2H, H36), 7.37 - 7.42 (m, 3H, H24, H36), 7.04 (app. td, *J* = 7.8, 1.4 Hz, 1H, H22), 6.90 (dd, *J* = 7.8, 1.4 Hz, 1H, H21), 6.77 (app. td, *J* = 7.8, 1.4 Hz, 1H, H23), 4.64 (s, 1H, H25), 4.55 - 4.59 (m, 1H, H32), 4.52 (d, *J* = 15.5 Hz, 1H, H34), 4.48 - 4.50 (m, 1H, H30), 4.35 (d, *J* = 15.5 Hz, 1H, H34), 3.87 - 3.93 (m, 1H, H29), 3.65 - 3.86 (m, 9H, H32, H12, H13), 2.46 (s, 3H, H42), 2.44 (t, *J* = 7.4 Hz, 2H, H10), 2.18 - 2.31 (m, 3H, H31, H2), 2.04 - 2.11 (m, 1H, H31), 1.57 - 1.66 (m, 4H, H3, H9), 1.31 - 1.38 (m, 10H, H4, H5, H6, H7, H8), 1.03 (s, 9H, H27).

^{13}C NMR (101 MHz, CD_3OD) δ_C ppm. 176.2 (C1), 174.7 (C11/33), 174.6 (C11/33), 172.5 (C28), 164.9 (C14), 156.9 (C18), 153.0 (C40), 149.2 (C41), 143.7 (C20), 143.0 (C17), 140.4 (C35), 134.4 (C16), 133.5 (C39), 131.6 (C38), 130.5 (C37), 130.2 (C15), 129.1 (C36), 128.1 (C24), 126.6 (C22), 125.5 (C19), 120.0 (C23), 118.9 (C21), 71.2 (C30), 61.0 (C32), 59.1 (C25), 58.2 (C29), 46.3 (C12/13), 45.4 (C12/13), 45.2 (C12/13), 43.8 (C34), 42.4 (C12/13), 39.1 (C31), 36.8 (C26), 36.7 (C2), 34.2 (C10), 30.65 (alkyl CH_2), 30.6 (alkyl CH_2), 30.55 (alkyl CH_2), 30.5 (alkyl CH_2), 30.4 (alkyl CH_2), 27.2 (C27), 27.1 (C3), 26.6 (C9), 16.0 (C42).

HRMS (ESI) m/z: $[M+H]^+$ calculated for $C_{48}H_{65}N_{10}O_6S$: 909.4809, found 909.4811.

Supporting Methods - Biology

4. Expression and purification of class I HDAC complexes in HEK293F cells

4.1 Culture of HEK293F cells

The HEK293F cell stock was maintained at a density of between 0.35 × 10^6 - 2.0 x 10^6 cells per mL using a stock volume of 60 mL in a 250 mL Erlenmeyer conical flask (Corning, Fisher Scientific). A 1 litre (490 cm^3) roller bottle (Corning, Fisher Scientific) was used to grow larger scale culture (maximum working volume of 300 mL). Cells were grown in FreeStyle™ 293 expression media (+ GlutaMAX™) (Gibco). Cultures were incubated in a 5% CO_2 shaking incubator (N-BIOTEK) at 120 RPM, 37 °C.

4.2 Transfection

For a 300 mL of cells transfection, 300 µg of total DNA (in 1:1 construct ratios) was diluted in 30 mL (1/10 of the transfection volume) of sterile PBS (Sigma). 600 µL of the polyethylenimine (PEI) transfection reagent (1 mg/mL, filtered) was added to the mixture (ratio of DNA to PEI - 1 µg:2 µg). The mixture was shaken vigorously and then incubated at room temperature for 30 minutes. The volumes and quantities of reagents were scaled proportionally to the working cell volumes. The PBS:DNA:PEI mixture was added to the cells at the density of 1 x 10^6 cells per mL. The cells were incubated in a 5% CO_2 shaking incubator (N-BIOTEK) at 120 RPM, 37 °C and then harvested by spinning at 4000 RPM (3501 × g, Sorvall SLC-6000) for 15 minutes at 4 °C after a 48-hour incubation. Harvested cells were either directly used for purification or stored at -80 °C.

4.3 Protein Purification

The HDAC complexes were purified as described previously.[3] Briefly, for a 1.2 L transfection, the cell pellet was dissolved in 30 mL of lysis buffer (50 mM Tris/Cl pH 7.5, 50 mM KOAc, 5% v/v glycerol, 0.4% v/v triton X-100, Roche complete EDTA free protease inhibitor (1 tablet per 50 mL)) and then lysed by sonication (QSONICA, 4 cycles of 15 seconds on, 15 seconds off, 40% amplitude). The insoluble material was removed by spinning at 30,000 RPM for 20 minutes at 4 °C (108,000 × g, Avanti™ J-30I, Beckman Coulter, Rotor JA30.50). The lysate was then incubated for 30 minutes at 4 °C with 1 mL of pre-washed anti-FLAG M2 resin (Sigma). After incubation, the protein-resin mixture was washed once with 10 mL of lysis buffer, four times with 10 mL of wash buffer (50 mM Tris/Cl pH 7.5, 50 mM KOAc, 5% v/v glycerol) and four times with 10 mL of cleavage buffer (50 mM Tris/Cl pH 7.5, 50 mM KOAc, 5% v/v glycerol, 0.5 mM TCEP). After the washing steps, the protein-resin mixture

was resuspended in 4 mL of cleavage buffer. 30-40 µL of 1.2 mg/mL Tobacco etch virus (TEV) protease was added to the mixture and incubated overnight at 4 °C. After TEV cleavage, the protein was concentrated to 500 µL using the 4 mL Amicon®Ultra centrifugal filter (Merck Millipore) with a membrane nominal molecular weight cut off of 10 kDa. The concentrated protein was then filtered through a 0.22 µm centrifugal filter (Merck Millipore).

The complexes were purified on the AKTA purifier system (GE Healthcare) using either Superose 6 (10/300 GL) or Superdex 200 (10/300 GL) columns (GE Healthcare), which were equilibrated in the gel filtration buffer (25 mM Tris/Cl pH 7.5, 50 mM KOAc, 0.5 mM TCEP) before sample loading. Fractions were collected in a 96-well plate (0.5 mL per fraction). 10 µL of sample from the fractions, which showed absorbance at 280 nm, was then mixed with 3 µL of the 6 × SDS sample buffer (60% glycerol, 375 mM Tris/Cl pH 6.8, 12% SDS, 300 mM DTT and 0.3 mg/mL bromophenol blue) for analysis by SDS-PAGE gel. Samples were run on a NuPAGE® 4-12% Bis-Tris gel (Novex) with NuPAGE® MES SDS running buffer at 200 V/125 mA/100 W until the bromophenol blue dye was at the bottom of gel. SeeBlue Plus2 pre-stained marker (Invitrogen) was used as the molecular weight marker and the gel was stained using Instant Blue Coomassie Stain (Expedeon). Fractions containing purified protein complex were concentrated using the 0.5 mL Amicon Ultra centrifugal filter (Merck Millipore) with a membrane nominal molecular weight cut off of 10 kDa. Concentration of the protein was determined using a protein assay dye reagent (Bio-Rad). 200 µL of the reagent was diluted in 800 µL of ddH_2O and used as a reagent blank. 2 µL of the protein sample was then added into the mixture and the absorbance was measured at 595 nm. The concentration of the protein sample in mg/mL was then calculated by multiplying by '17', which represents a factor calculated from a BSA standard curve. The protein complexes were stored by flash freezing in liquid nitrogen in the presence of 25% glycerol before being transferred to a freezer at −80 °C.

5. HDAC assay

HDAC activity and inhibition experiments against class I HDAC complexes were carried out using a fluorescent HDAC assay. The assays were conducted on black 96-well plates (Corning #3915) with a final reaction volume of 50 µL. 100 µM of Boc- (Ac)Lys-AMC was used as a substrate in each well. All determinations were performed in triplicate. The inhibitors were dissolved at 50 mM in DMSO, and then further diluted with 10% DMSO HDAC assay buffer (10% DMSO, 50 mM Tris pH 7.5, 50 mM NaCl, 0.1 mg/mL BSA) to micromolar concentrations. Serial dilutions of compounds were then carried out using 10% DMSO HDAC assay buffer to afford a range of concentrations. 10 µL of each of these solutions were added to individual wells, followed by addition of 30 µL of the HDAC complex (12.5 nM final concentration) dissolved in HDAC assay buffer (50 mM Tris pH 7.5, 50 mM NaCl, 0.1 mg/mL BSA). For the inhibition experiments with inositol phosphates, HDAC complex was pre-incubated with inositol hexaphosphate (100 µM final concentration) for 30 minutes at room temperature. The HDAC

complex and inhibitor were then incubated at 20 °C, 100 RPM for 2 hours. After incubation, 10 μL of the Boc-(Ac)Lys-AMC substrate, dissolved in HDAC assay buffer, was added to each well. The plate was incubated at 30 °C, 100 RPM for 1 hour, followed by addition of 50 μL of a developer buffer (50 mM Tris pH 7.5, 100 mM NaCl, 10 mg/mL trypsin) to quench the reaction. The reaction was allowed to develop for 10 minutes at 30 °C, 100 RPM. Fluorescence intensity was determined with the Victor X5 plate reader (Perkin Elmer, λ_{ex} = 355 nm, λ_{em} = 460 nm). IC_{50} values were calculated using GraphPad Prism 7 software by non-linear regression, log (inhibitor) vs. response – variable slope (four parameters).

6. Culture of HCT116 cells

HCT116 cells were maintained on 100 mm or 60 mm culture dishes in Dulbecco's Modified Eagle Medium (Gibco) supplemented with 10% fetal bovine serum (Sigma) and 1X glutamine/penicillin/streptomycin (Gibco). Once the cells reached ~90% confluency, they were washed with warm PBS (Sigma), trypsinised using TrypLE™ Express solution (Gibco) and put into an incubator at 37°C for 5 minutes. Once dissociated, cell media was used to dilute the cells into required density, after which they were either split or seeded for inhibitor/PROTAC treatment.

7. Compound treatment of HCT116 cells

All compounds were made up to a standard solution of 10 mM in DMSO, serially diluted in DMSO, then added to fresh medium to afford the required concentrations, with a final DMSO concentration of 0.1%. 24 hours before treatment with inhibitors, HCT116 cells were seeded at a density of 1.4×10^6 cells in a 100 mm dish. For the PROTAC treatment, the cells were seeded into 6-well plates at a density of 4×10^5 cells/well. Cells were then treated with inhibitors/PROTACs at the indicated concentrations in fresh media, as well as, DMSO control. After 24 hours of treatment, the cells were harvested by removing media and washing with chilled PBS (Sigma). Small amount of PBS (Sigma) was added to the dishes and the cells were harvested by scrapping. Cells were collected and centrifuged for 4 minutes, 2000 RPM, 4 °C. The supernatant was removed and the cells were snap-frozen using liquid N_2 and stored at -80 °C.

8. Protein Extraction

The cells were lysed in cell lysis buffer (50 mM Tris-HCl, 150 mM NaCl, 0.5% NP-40 (IGEPAL), 0.5% Triton X-100) supplemented with a protease inhibitor (Roche 05056489001) (1 tablet per 25 mL of cell lysis buffer). The cell suspension was incubated on ice for 30 mins and then centrifuged (18,000 rcf, 15

mins, 4 °C) and the supernatant was collected. Protein concentrations were quantified *via* Bradford Assay using Protein Assay Dye Reagent Concentrate (BIO-RAD). For histone extraction, the pellets were washed with cell lysis buffer (3 x 200 µL) and then an equal volume of 0.4 N H_2SO_4 was added. The extracts were placed at 4 °C overnight, centrifuged (18,000 rcf, 15 min, 4 °C), and then the supernatant collected.

9. Western Blotting

Western blots were run on NuPAGE™ 4-12%, Bis-Tris gels (ThermoFisher Scientific, NP0329BOX) with 30 µg of protein or 5 µL of acid-extracted histone loaded per lane and using NuPAGE™ MES SDS (ThermoFisher Scientific, NP0002) as the running buffer. Protein samples were prepared for western blot by denaturing in SDS PAGE loading dye (6X), and boiling at 95 °C for 2-3 mins. SeeBlue™ Plus2 Pre-stained Protein Standard (ThermoFisher Scientific, LC5925) PageRuler Plus Prestained Ladder was used as the molecular weight marker. The proteins were separated by gel electrophoresis at 140 V for 75 mins and then transferred onto a nitrocellulose membrane at 30 V for 60 mins. The membrane was then dried for 1 hour, after which it was incubated in the blocking buffer (5% skimmed milk powder in PBS-T (0.05% v/v Tween)) for 1 hour at room temperature. The membranes were then incubated with the primary antibodies in 2.5% skimmed milk in PBS-T (as indicated below) overnight at 4 °C. The membranes were washed with PSB-T (3 x 30 secs and 3 x 5 mins washes). Blots were developed using complimentary IRDye®-conjugated secondary antibodies, diluted in 2.5% skimmed milk in PBS-T, for 50 mins at room temperature. The membranes were washed with PBS-T (3 x 30 secs and 3 x 5 mins washes), before being visualised using LI-COR Odyssey Infrared Imaging System. Data was analysed using LICOR Image StudioTM Lite Software.

<u>Antibody Information</u>

Antibody	Manufacturer	Dilution
HDAC1	Abcam, 109411	1:2,000
HDAC3	Abcam, 32369	1:2,000
α-tubulin	Sigma, t5168	1:10,000
H3K56ac	Active Motif, 39082	1:1,000
H3	Merck Millipore, 05-499<br>Active Motif, 39763	1:1,000<br>1:2,000
IRDye® 680LT	LI-COR Biosciences, 926-68023	1:10,000
IRDye® 800CW	LI-COR Biosciences, 926-32210	1:10,000

Supporting Figures and Tables

Components of HDAC complexes

Table S1 - HDAC complexes used for HDAC assays. Unless otherwise stated, full-length proteins were used.

HDAC Complex	Complex Components
HDAC1-CoREST	HDAC1, CoREST (86-485), LSD1
HDAC2-CoREST	HDAC2, CoREST (86-485), LSD1
HDAC3-SMRT	HDAC3, SMRT/GPS2 (1-49/220-480), TBL1 (H23A/F26A/I30A; tetramerization mutant)
HDAC1-MIER1	HDAC1, MIER1, H2A, H2B
HDAC1-MiDAC	HDAC1, DNTTIP1, MIDEAS (628-887)
HDAC1-NuRD	HDAC1, MTA1, MBD2 (145-411), RBBP4/7
HDAC1-RERE	HDAC1, RERE (1-565)

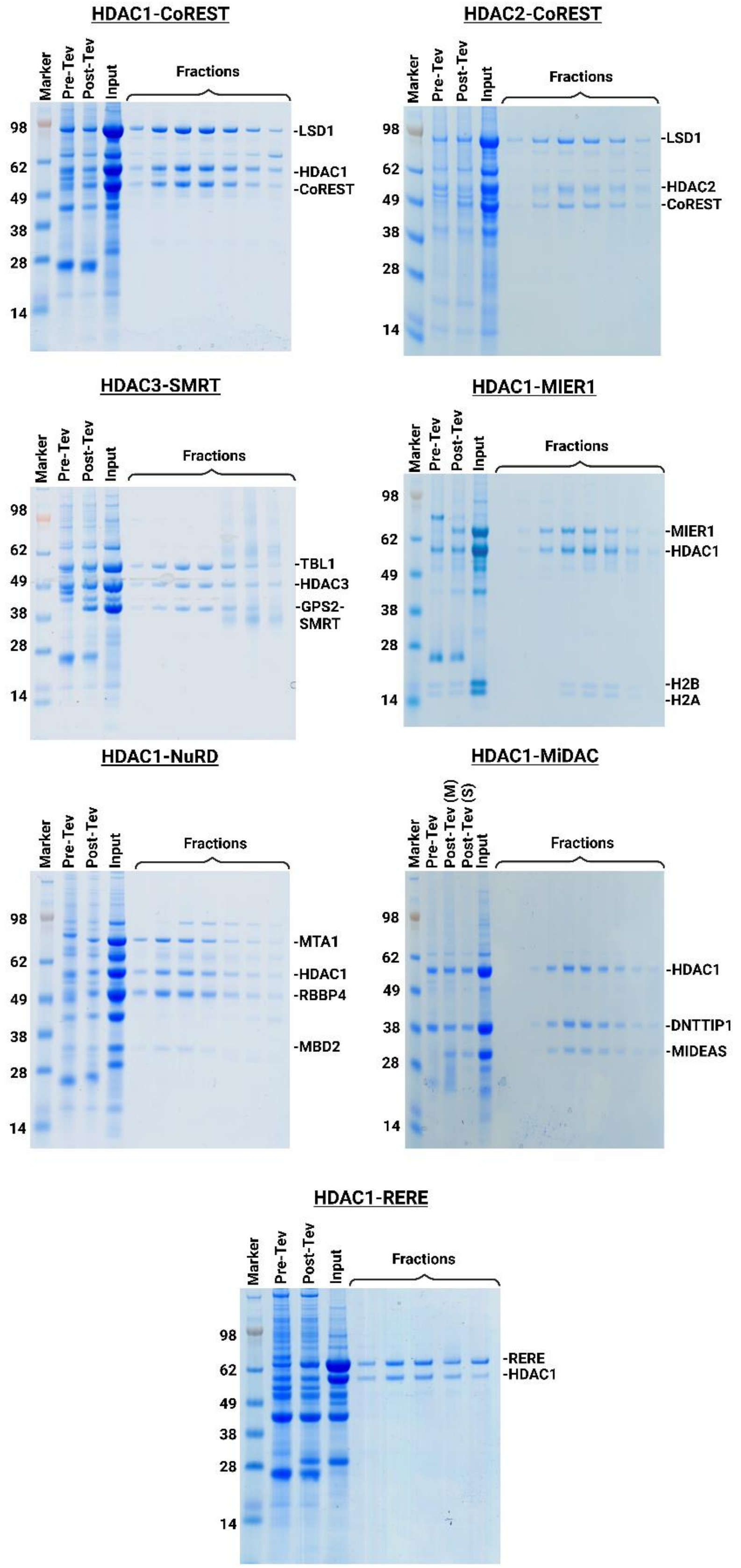

Figure S1 - SDS-PAGE gels showing purification of the class I HDAC complexes used in this study. Control samples from pre-TEV cleavage resin, TEV-cleaved resin and gel filtration input, alongside the gel filtration fractions which had a UV absorbance of 280 nm were run on a NuPAGE™ 4-12% Bis-Tris gel. The gels were stained with Coomassie Instant Blue stain.

Inhibition Curves

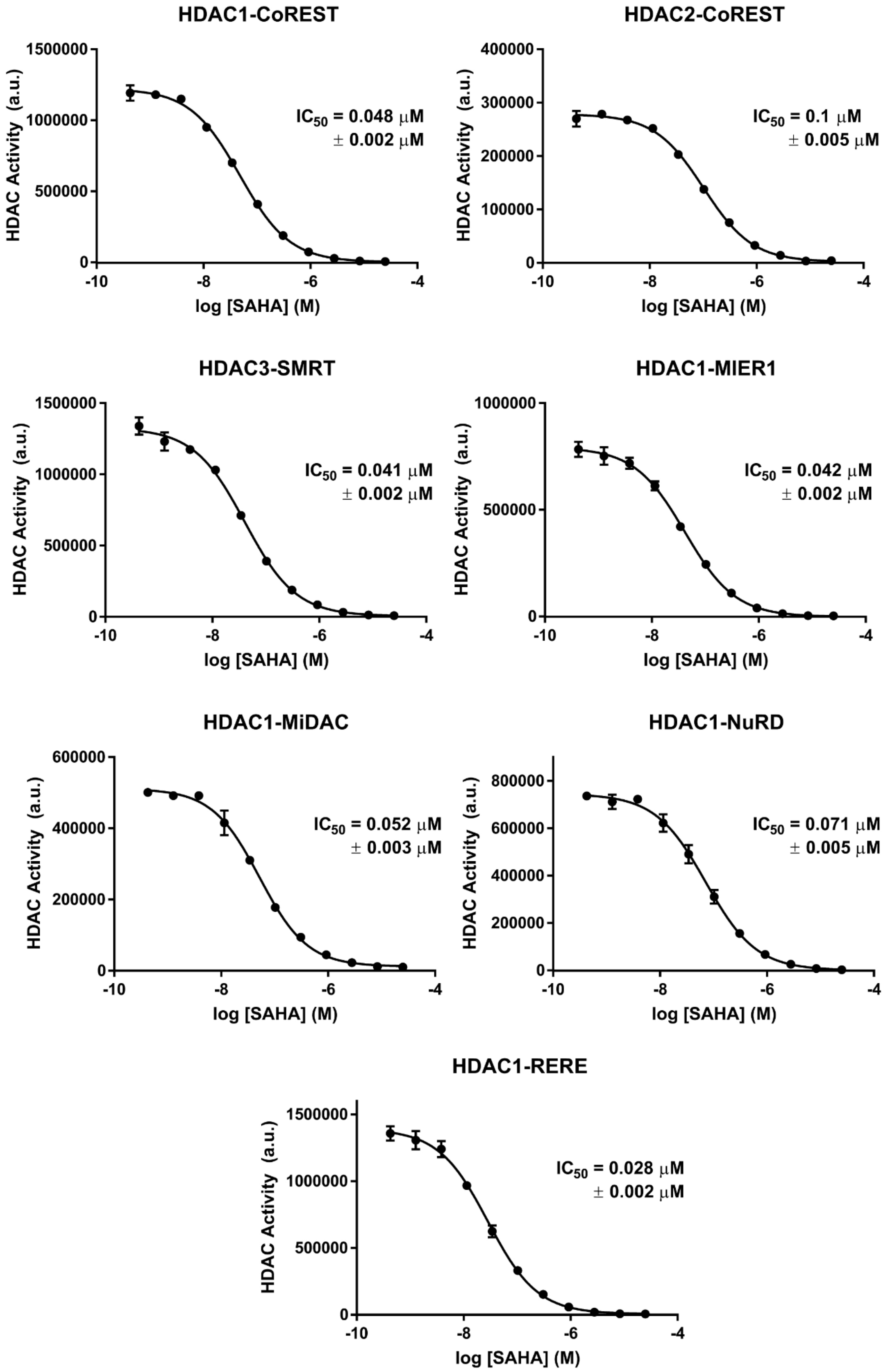

Figure S2 - Inhibition curves of SAHA against class I HDAC complexes. Experiments were performed in technical triplicate. Error bars represent ± S.E.M. (n = 3).

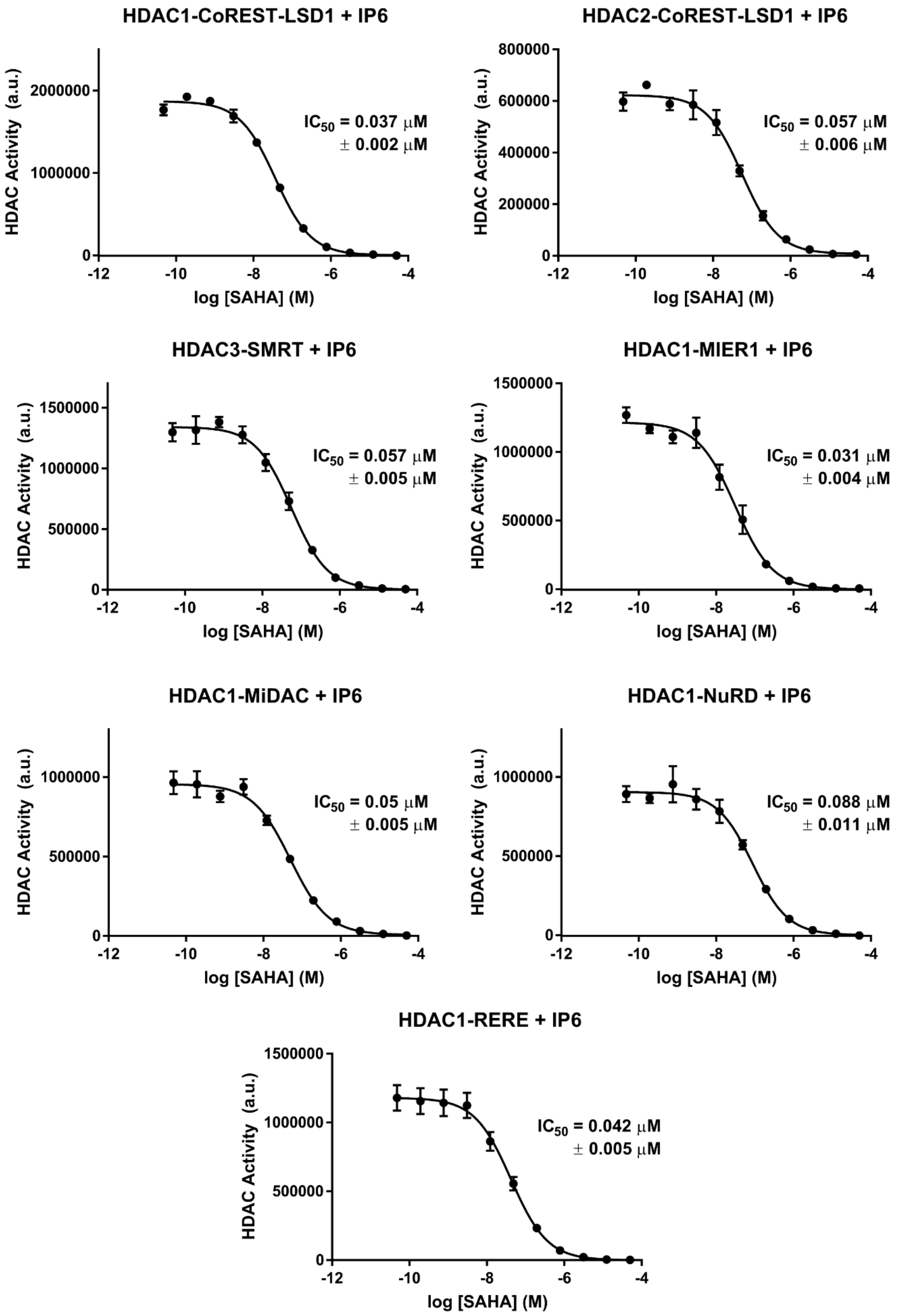

Figure S3 - Inhibition curves of SAHA, in the presence of $InsP_6$, against class I HDAC complexes. Experiments were performed in technical triplicate. Error bars represent ± S.E.M. (n = 3).

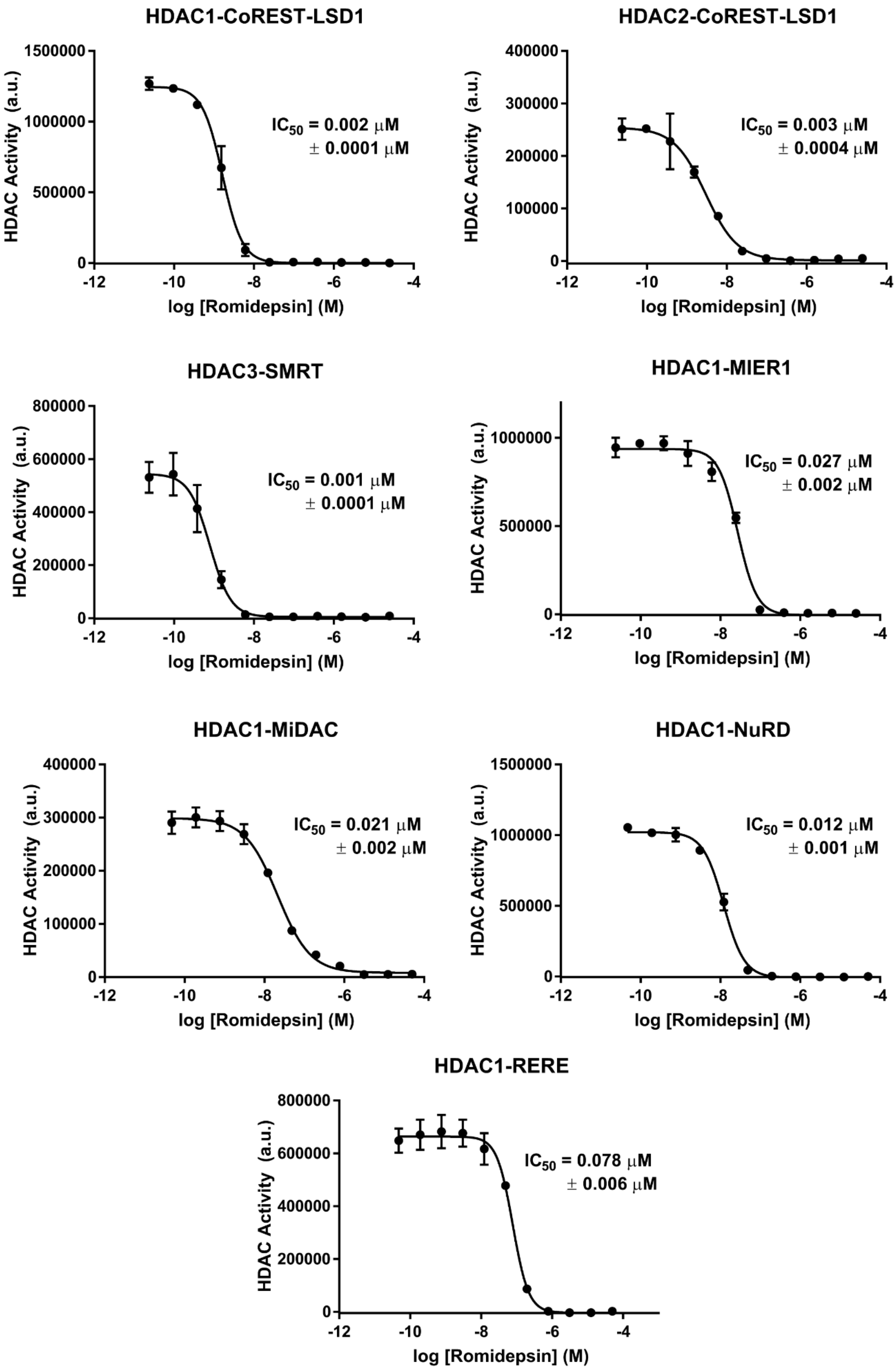

Figure S4 - Inhibition curves of Romidepsin against class I HDAC complexes. Experiments were performed in technical triplicate. Error bars represent ± S.E.M. (n = 3).

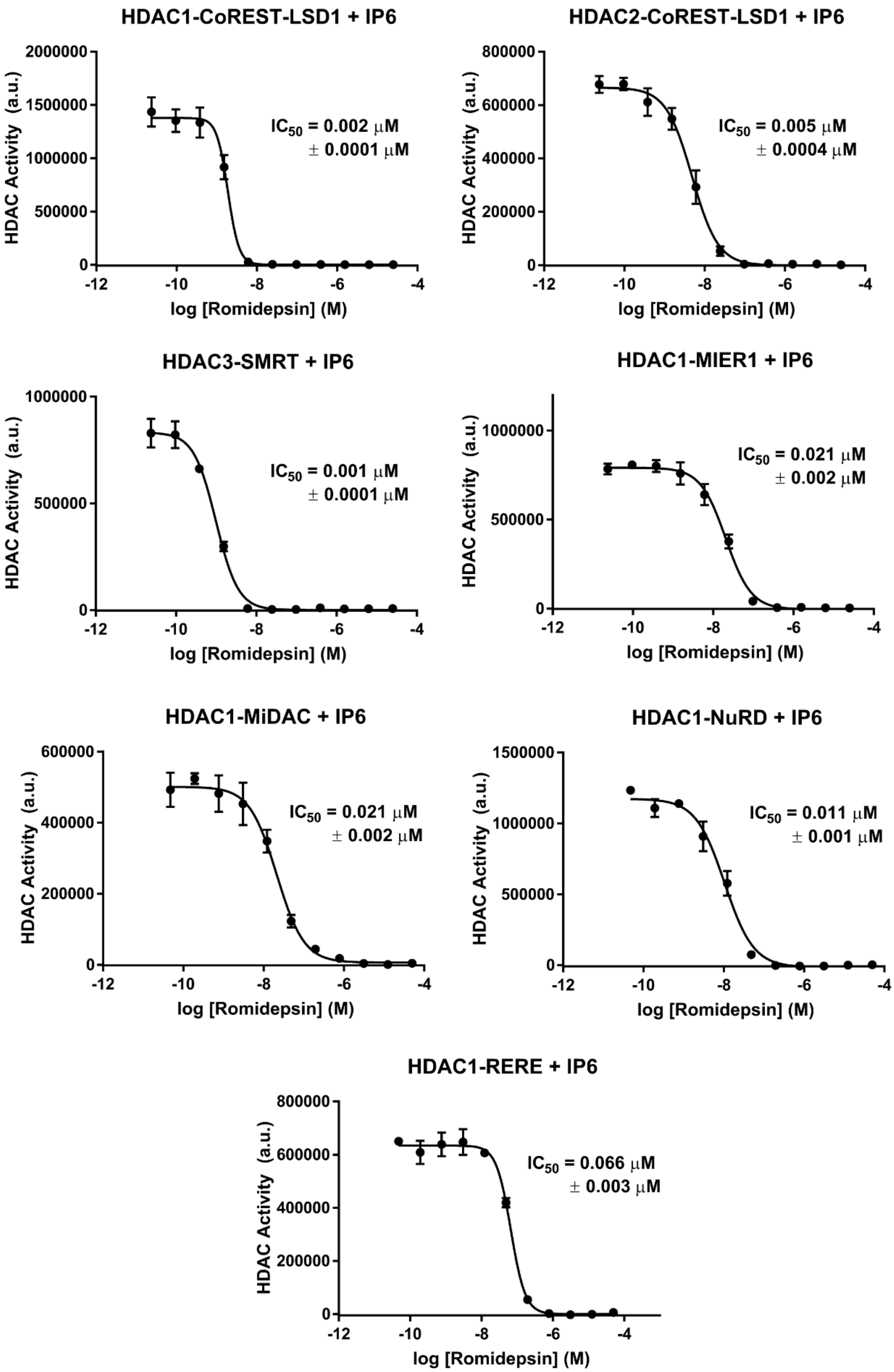

Figure S5 - Inhibition curves of Romidepsin, in the presence of $InsP_6$, against class I HDAC complexes. Experiments were performed in technical triplicate. Error bars represent ± S.E.M. (n = 3).

MS-275

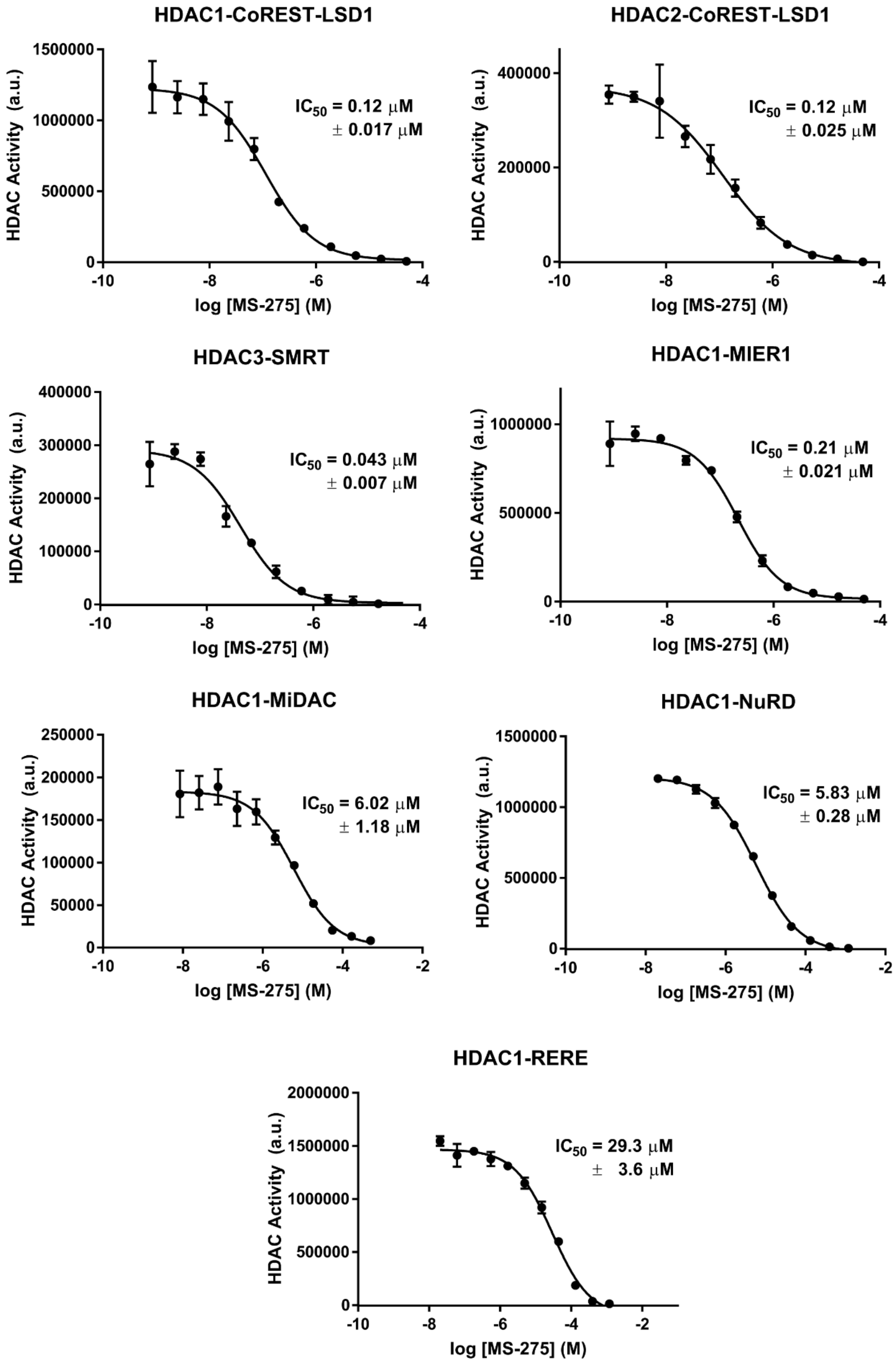

Figure S6 - Inhibition curves of MS-275 against class I HDAC complexes. Experiments were performed in technical triplicate. Error bars represent ± S.E.M. (n = 3).

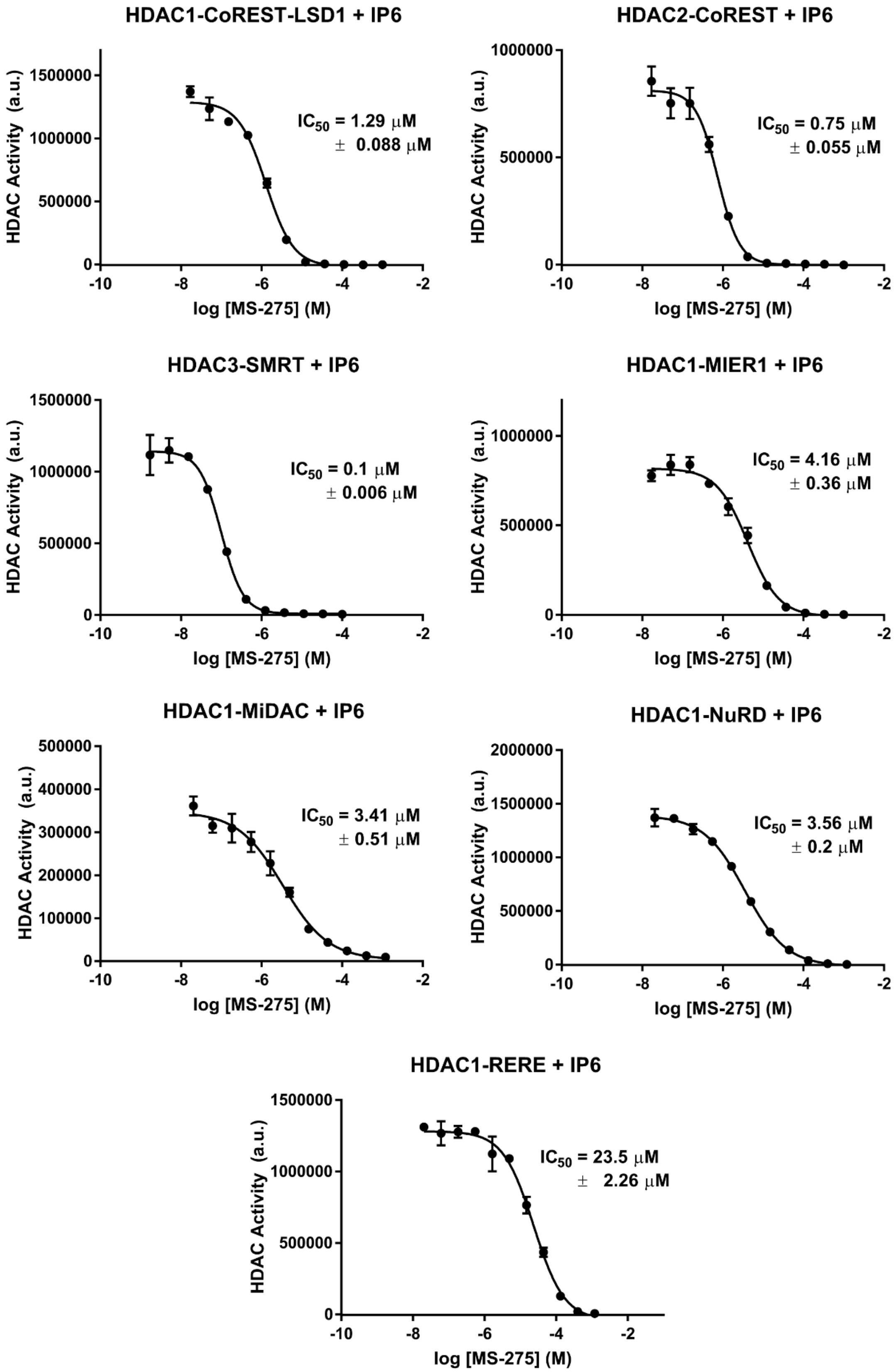

Figure S7 - Inhibition curves of MS-275, in the presence of $InsP_6$, against class I HDAC complexes. Experiments were performed in technical triplicate. Error bars represent ± S.E.M. (n = 3).

CI-994

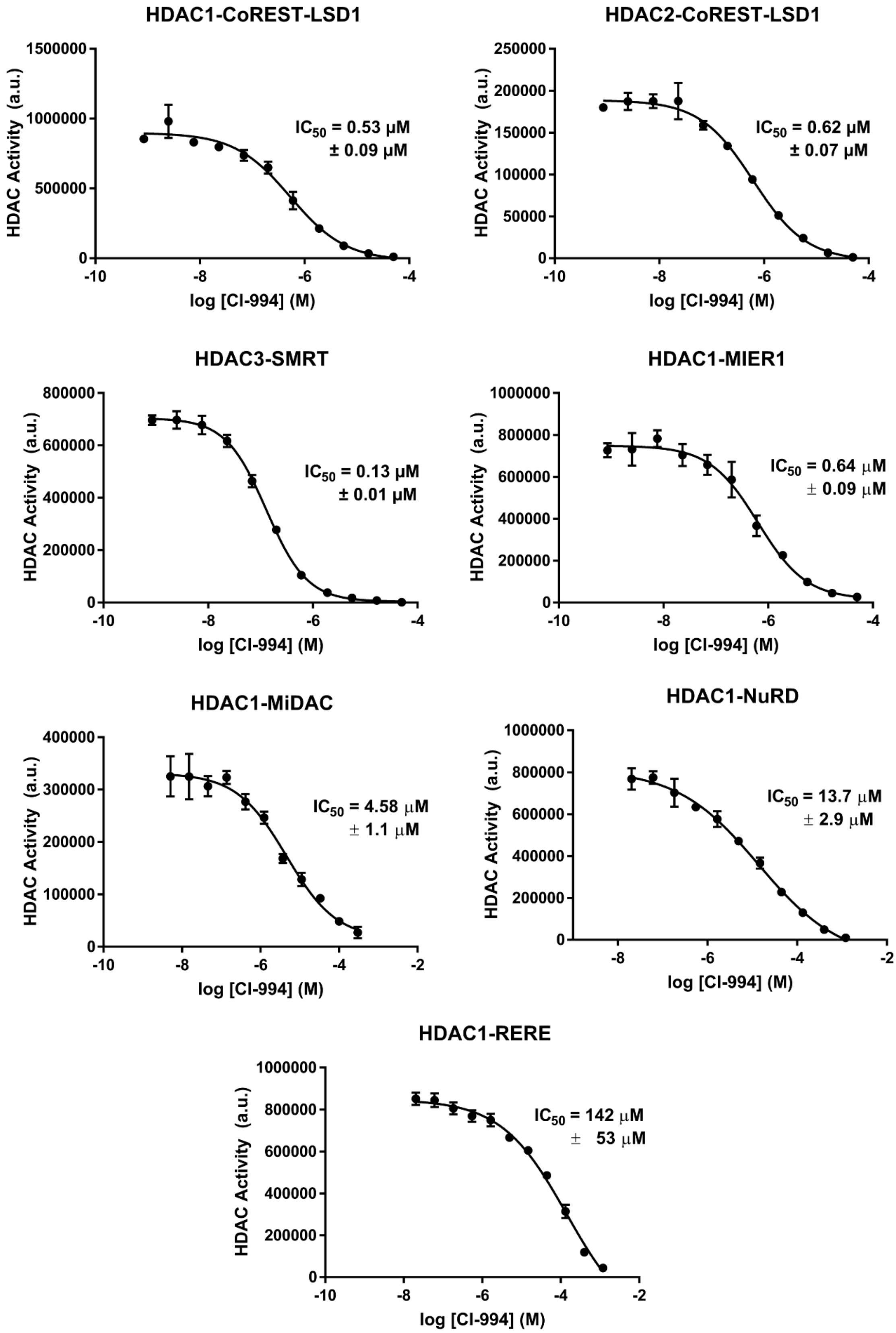

Figure S8 - Inhibition curves of CI-994 against class I HDAC complexes. Experiments were performed in technical triplicate. Error bars represent ± S.E.M. (n = 3).

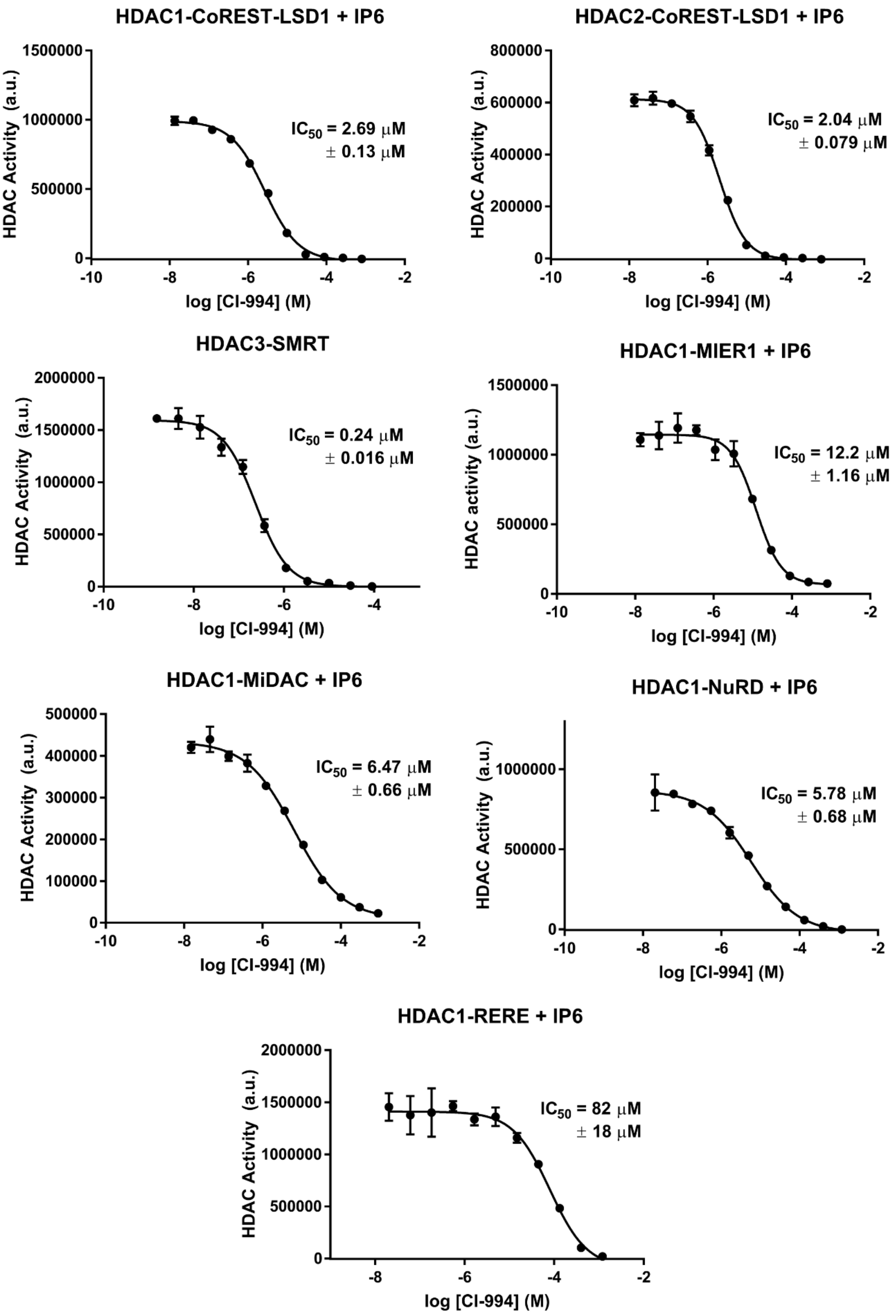

Figure S9 - Inhibition curves of CI-994, in the presence of $InsP_6$, against class I HDAC complexes. Experiments were performed in technical triplicate. Error bars represent ± S.E.M. (n = 3).

CPD-60

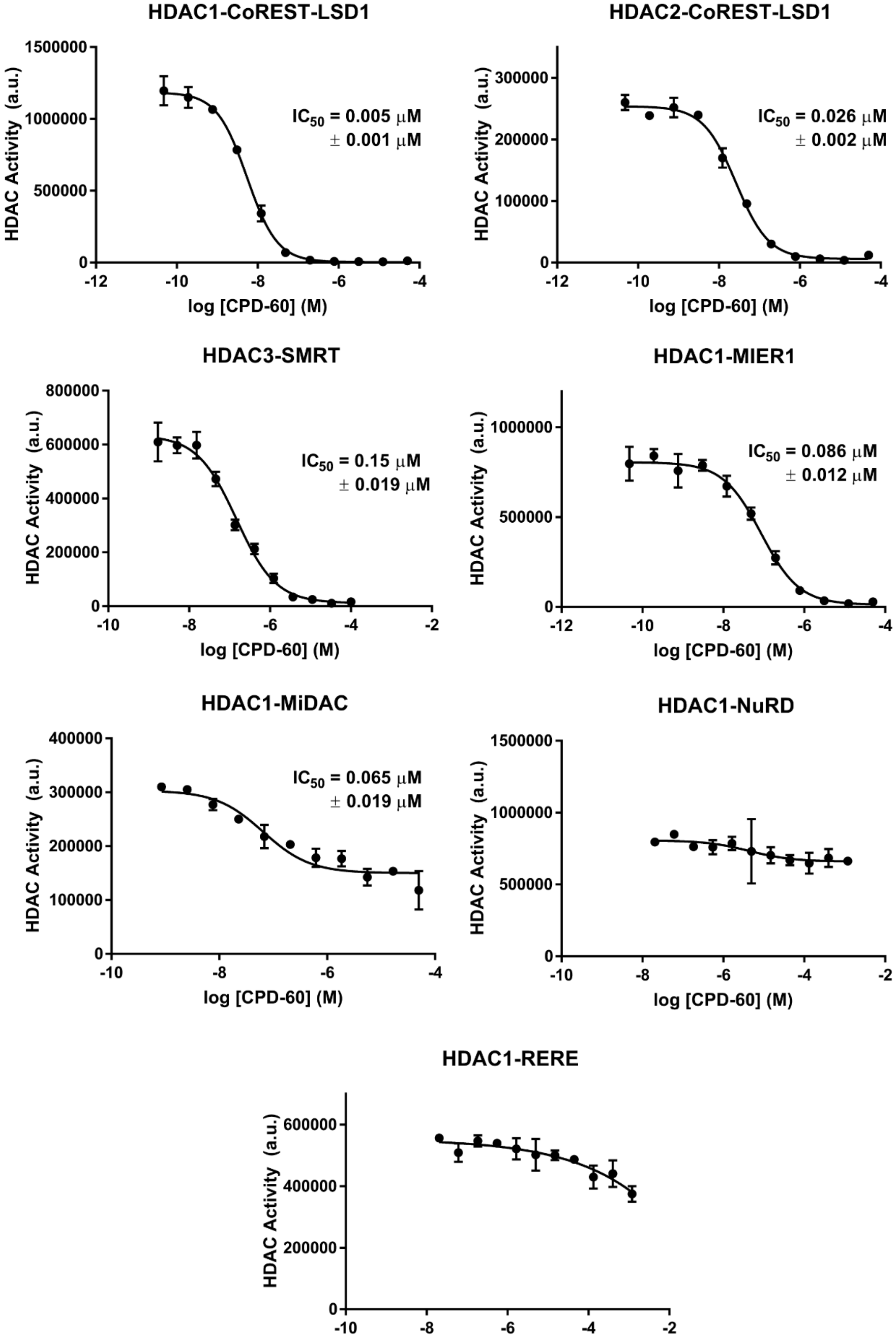

Figure S10 - Inhibition curves of CPD-60 against class I HDAC complexes. Experiments were performed in technical triplicate. Error bars represent ± S.E.M. (n = 3).

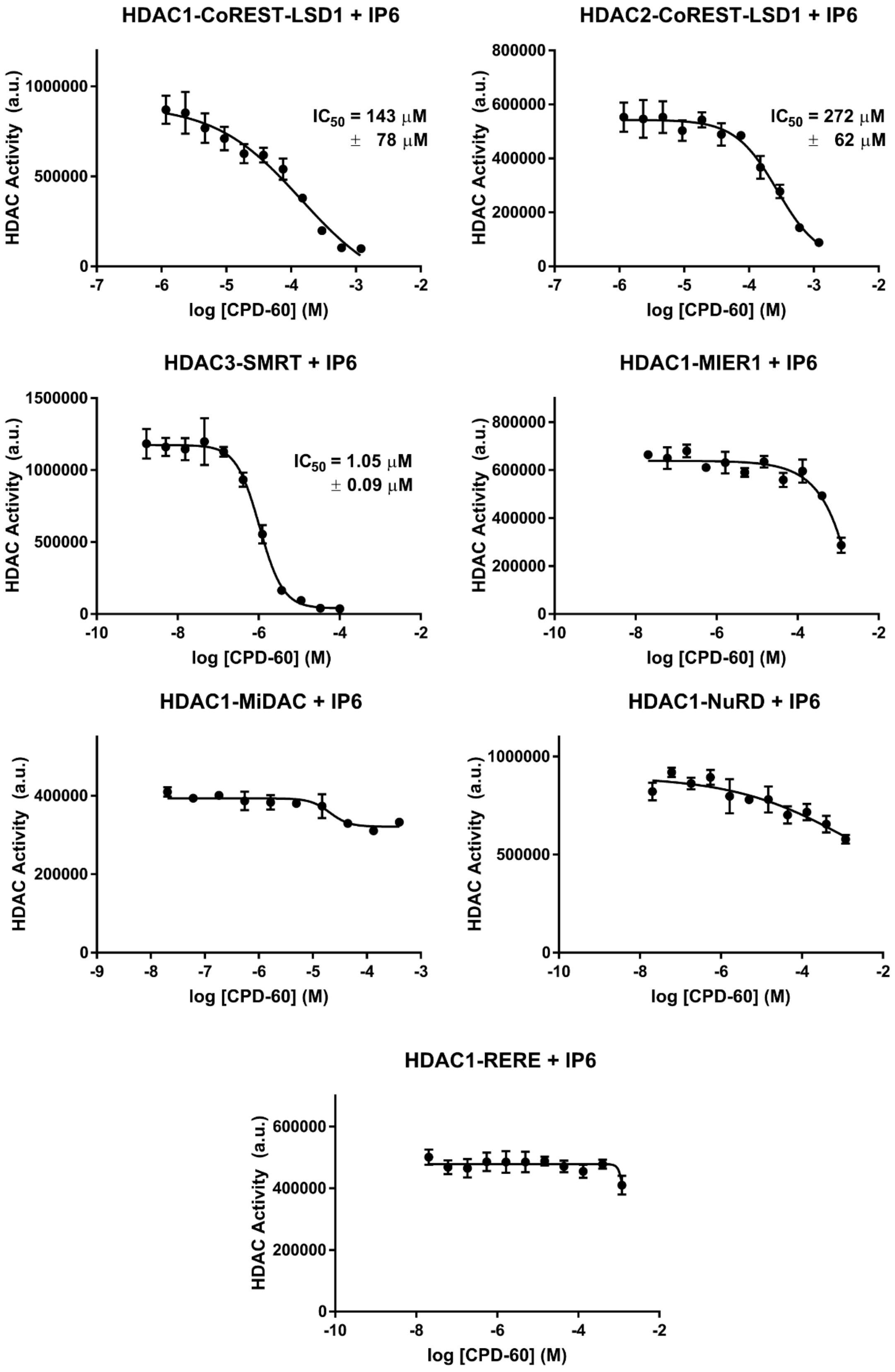

Figure S11 - Inhibition curves of CPD-60, in the presence of $InsP_6$, against class I HDAC complexes. Experiments were performed in technical triplicate. Error bars represent ± S.E.M. (n = 3).

Compound 1

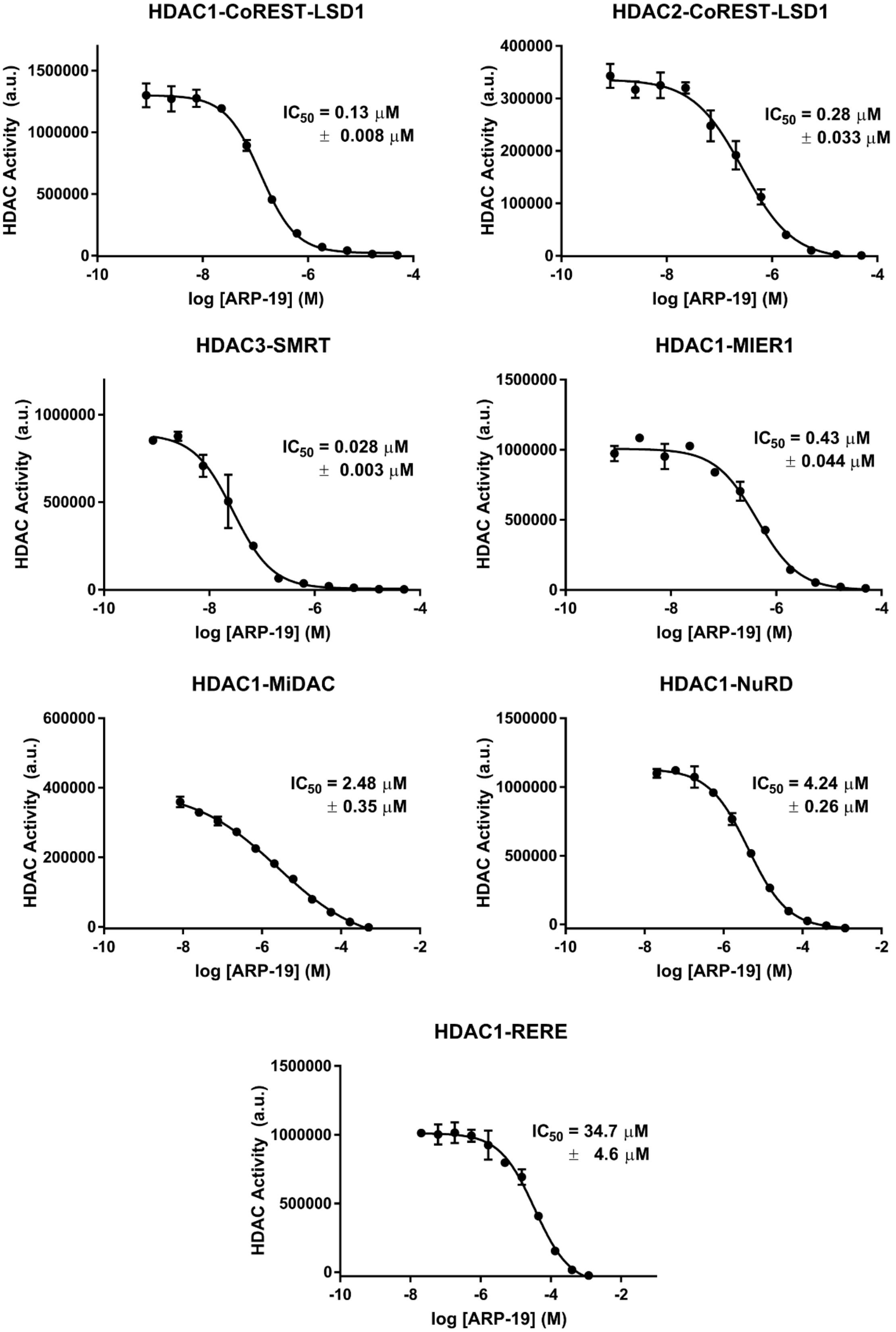

Figure S12 - Inhibition curves of Compound 1 against class I HDAC complexes. Experiments were performed in technical triplicate. Error bars represent ± S.E.M. (n = 3).

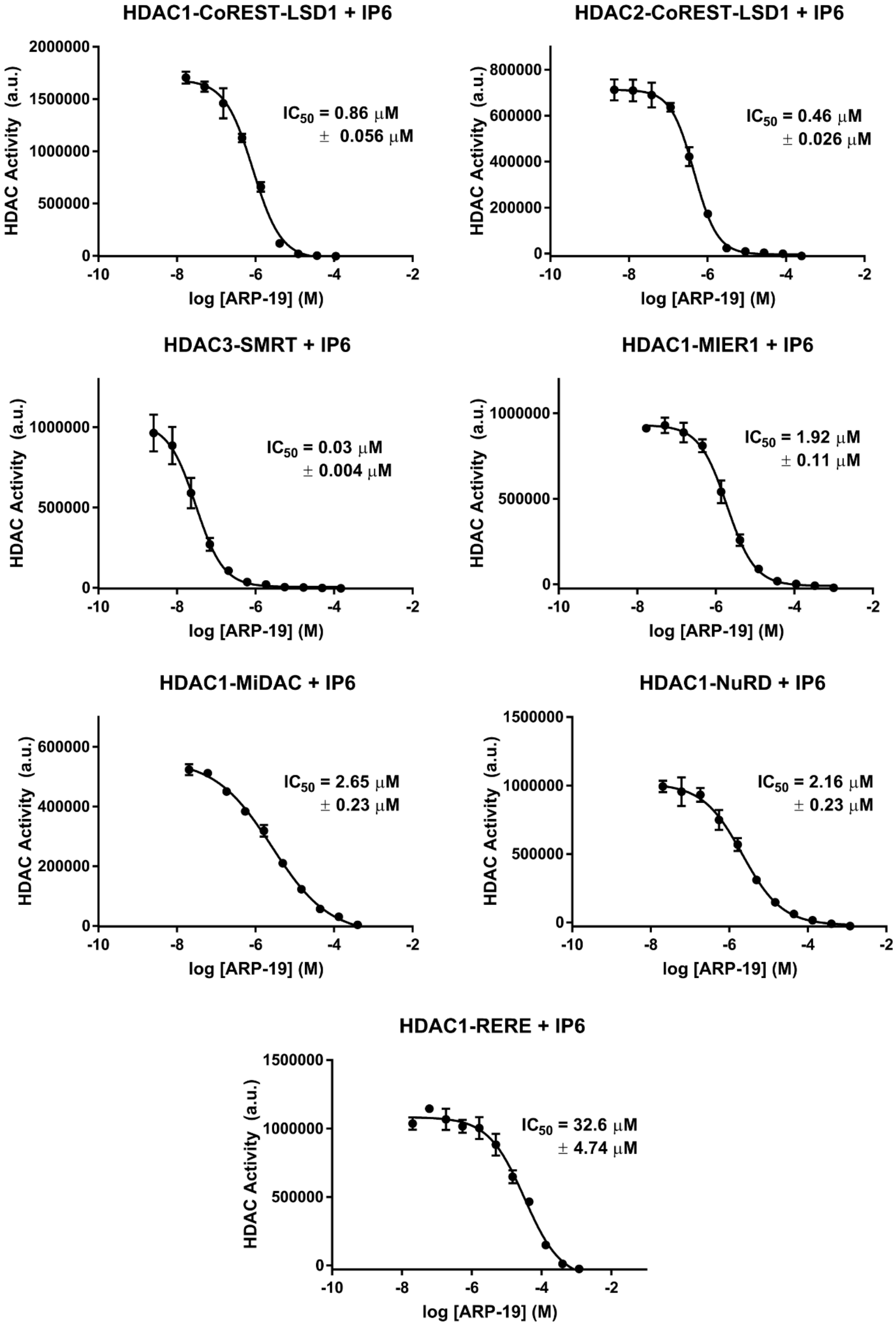

Figure S13 - Inhibition curves of Compound 1, in the presence of $InsP_6$, against class I HDAC complexes. Experiments were performed in technical triplicate. Error bars represent ± S.E.M. (n = 3).

Compound 2

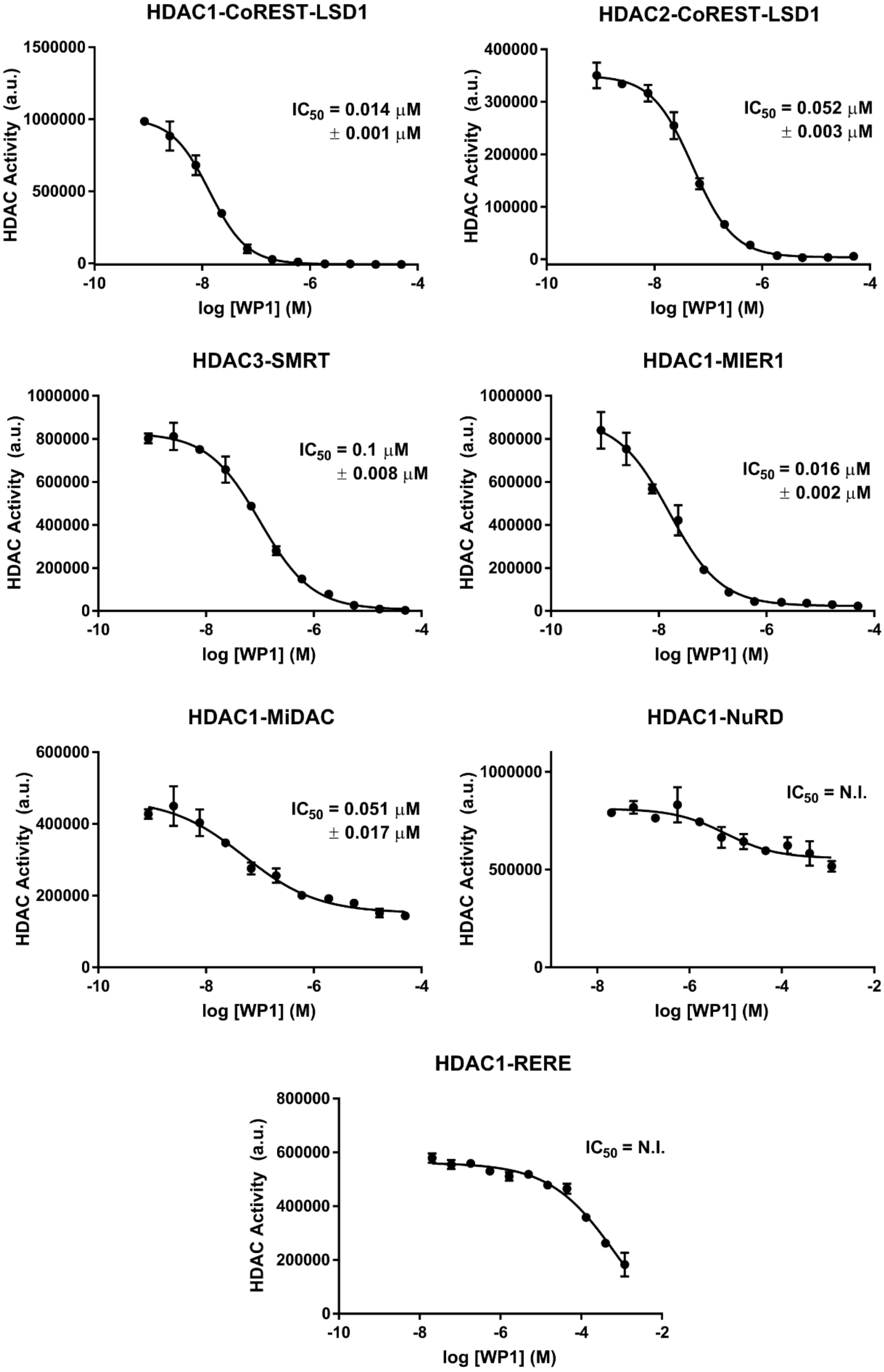

Figure S14 - Inhibition curves of Compound 2 against class I HDAC complexes. Experiments were performed in technical triplicate. Error bars represent ± S.E.M. (n = 3).

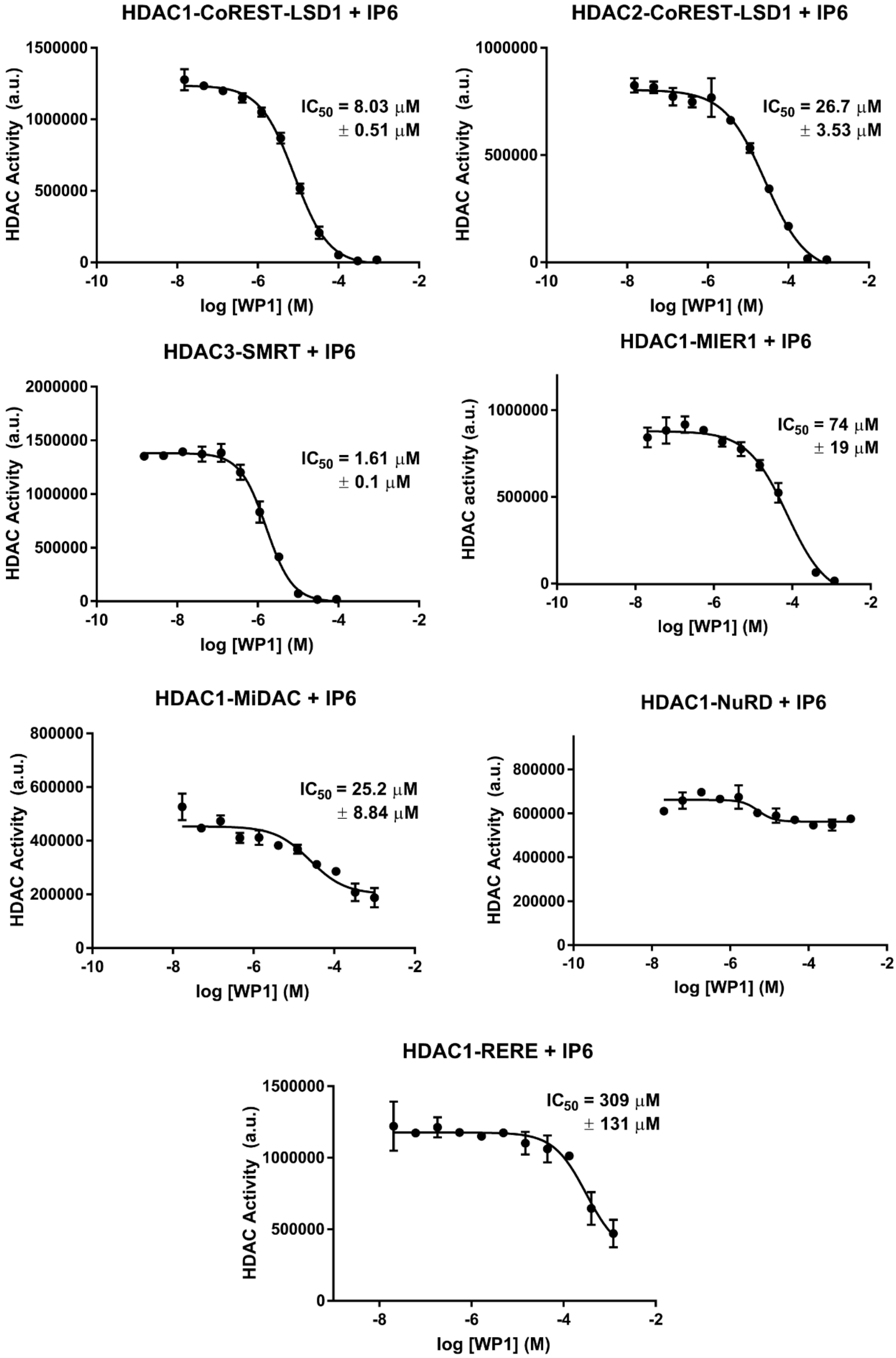

Figure S15 - Inhibition curves of Compound 2, in the presence of $InsP_6$, against class I HDAC complexes. Experiments were performed in technical triplicate. Error bars represent ± S.E.M. (n = 3).

Processed histone acetylation immunoblots

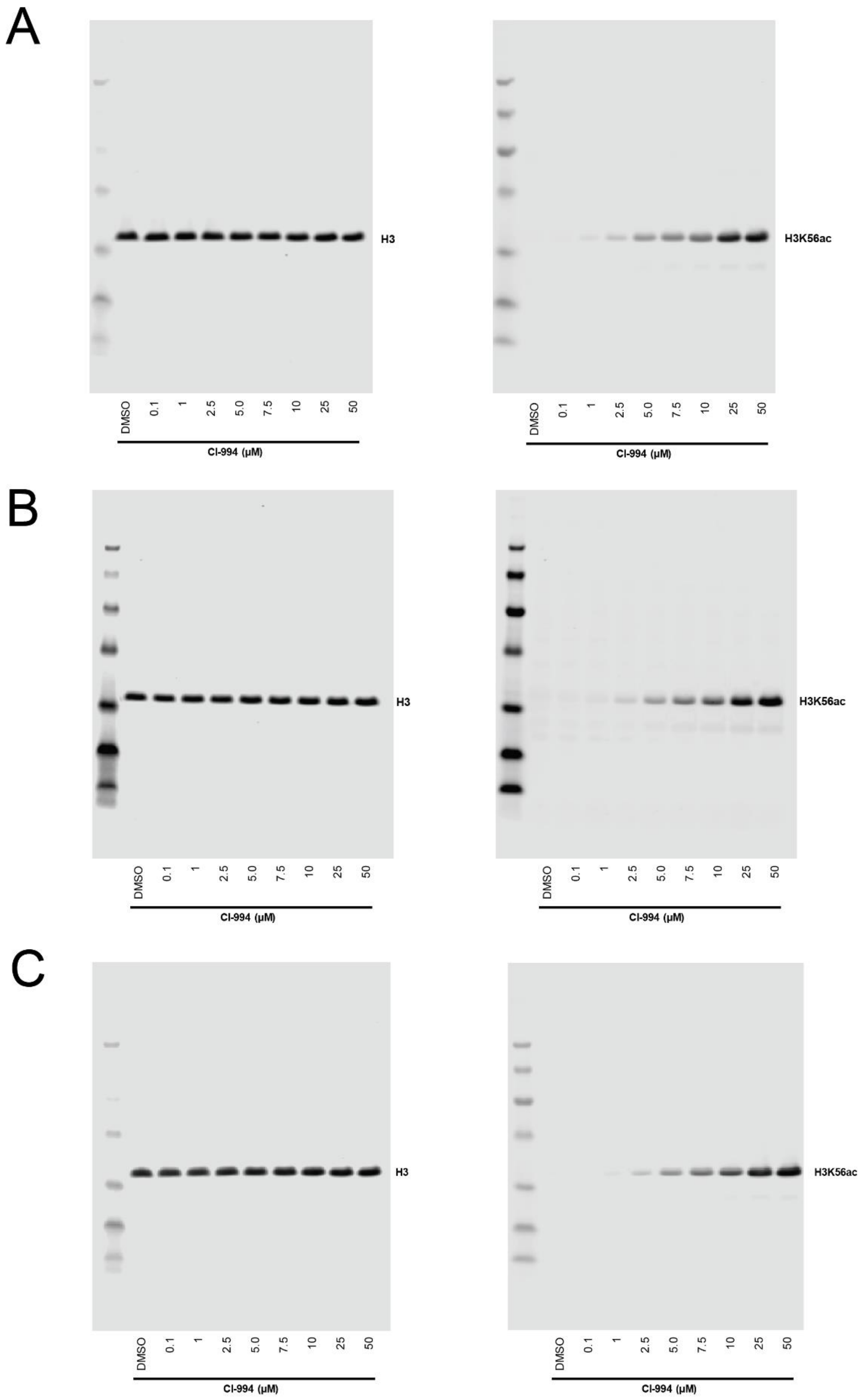

Figure S16 - Processed immunoblots of the H3K56Ac and H3 levels in HCT116 cells following a 24 h treatment with increasing concentrations of CI-994; (A) Biological replicate 1; (B) Biological replicate 2; (C) Biological replicate 3.

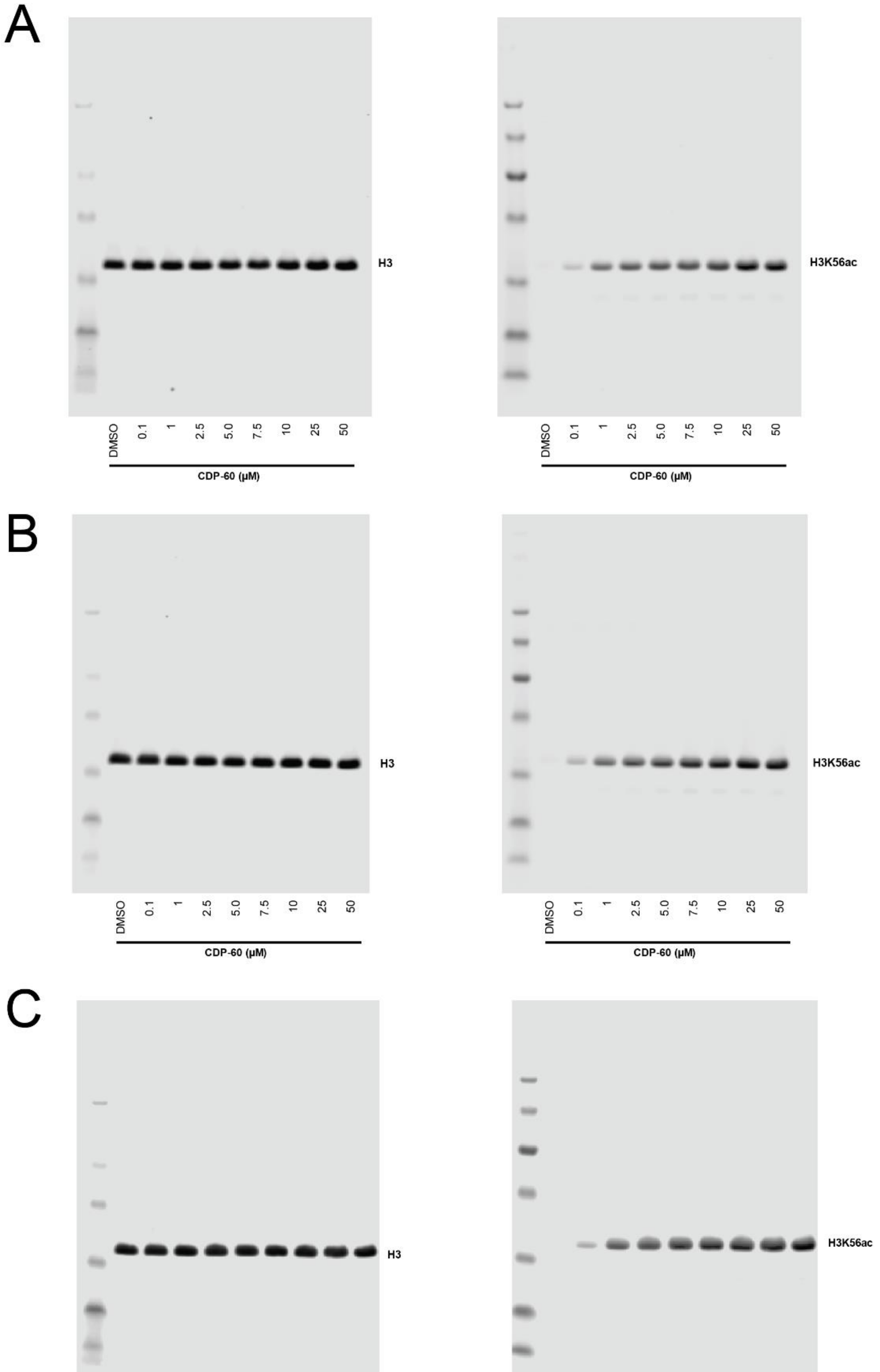

Figure S17 - Processed immunoblots of the H3K56Ac and H3 levels in HCT116 cells following a 24 h treatment with increasing concentrations of CDP-60; (A) Biological replicate 1; (B) Biological replicate 2; (C) Biological replicate 3.

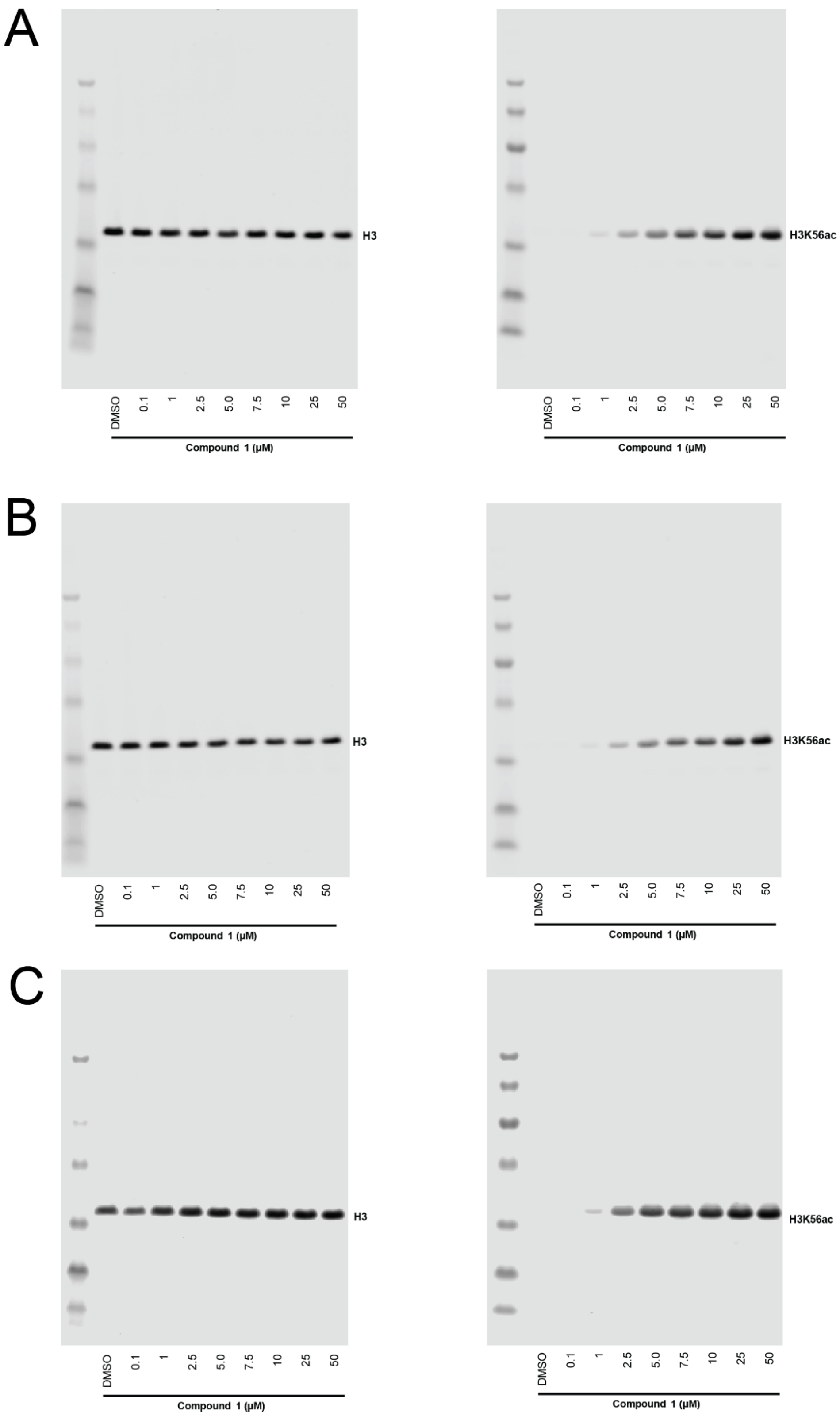

Figure S18 - Processed immunoblots of the H3K56Ac and H3 levels in HCT116 cells following a 24 h treatment with increasing concentrations of Compound 1; (A) Biological replicate 1; (B) Biological replicate 2; (C) Biological replicate 3.

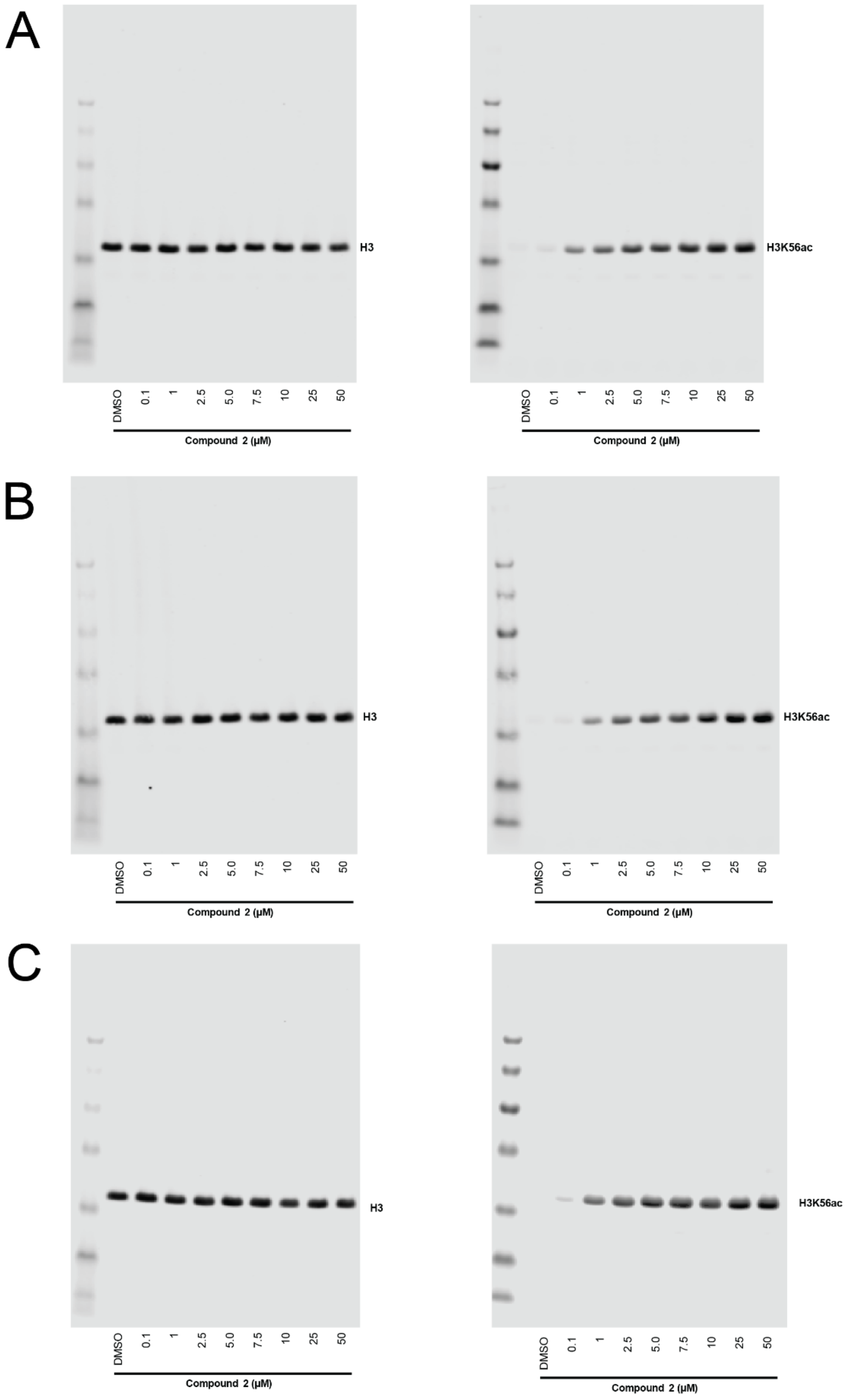

Figure S19 - Processed immunoblots of the H3K56Ac and H3 levels in HCT116 cells following a 24 h treatment with increasing concentrations of Compound 2; (A) Biological replicate 1; (B) Biological replicate 2; (C) Biological replicate 3.

Unprocessed immunoblots

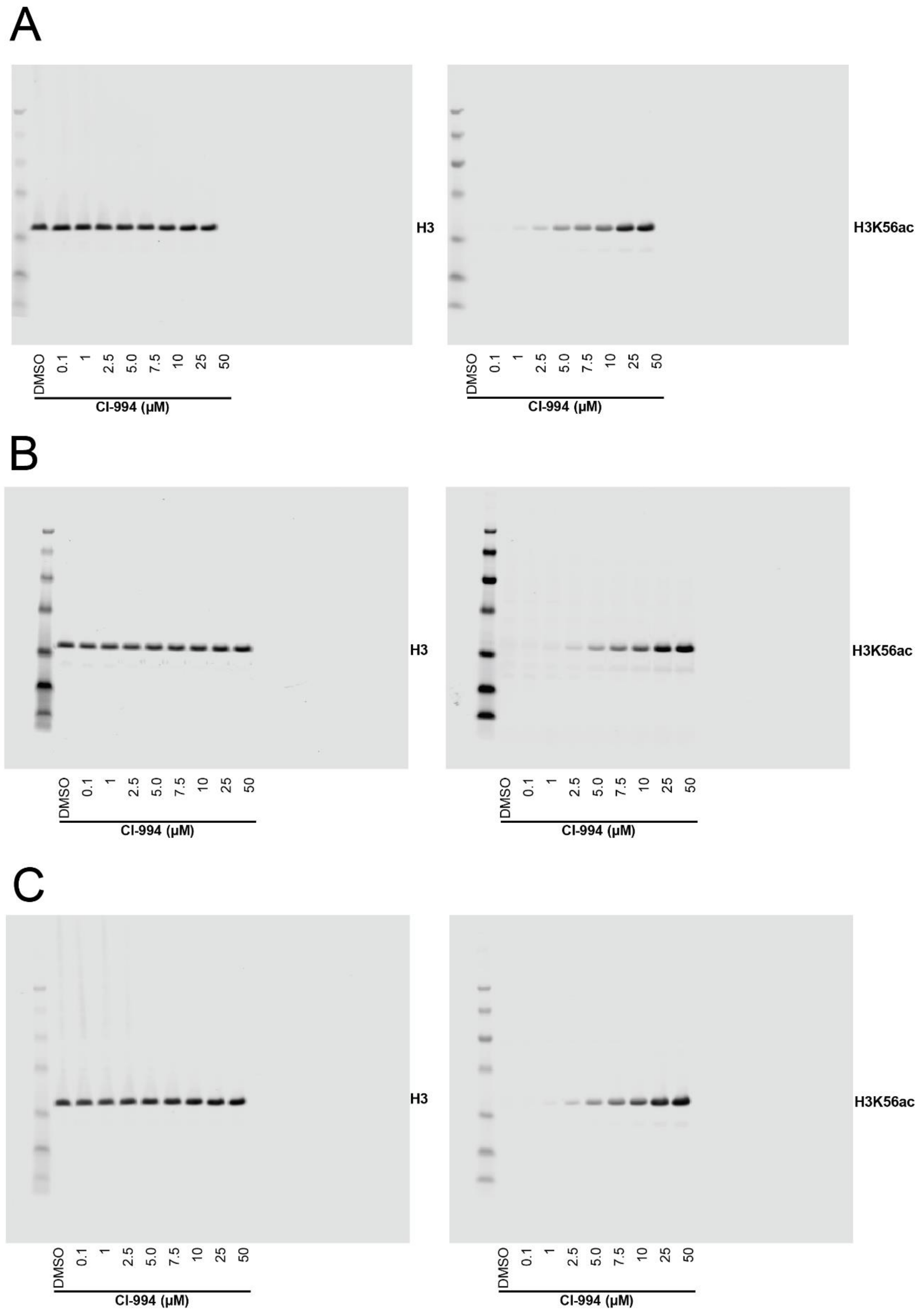

Figure S20 – Unprocessed immunoblots of the H3K56Ac and H3 levels in HCT116 cells following a 24 h treatment with increasing concentrations of CI-994; (A) Biological replicate 1 (B) Biological replicate 2 (C) Biological replicate 3.

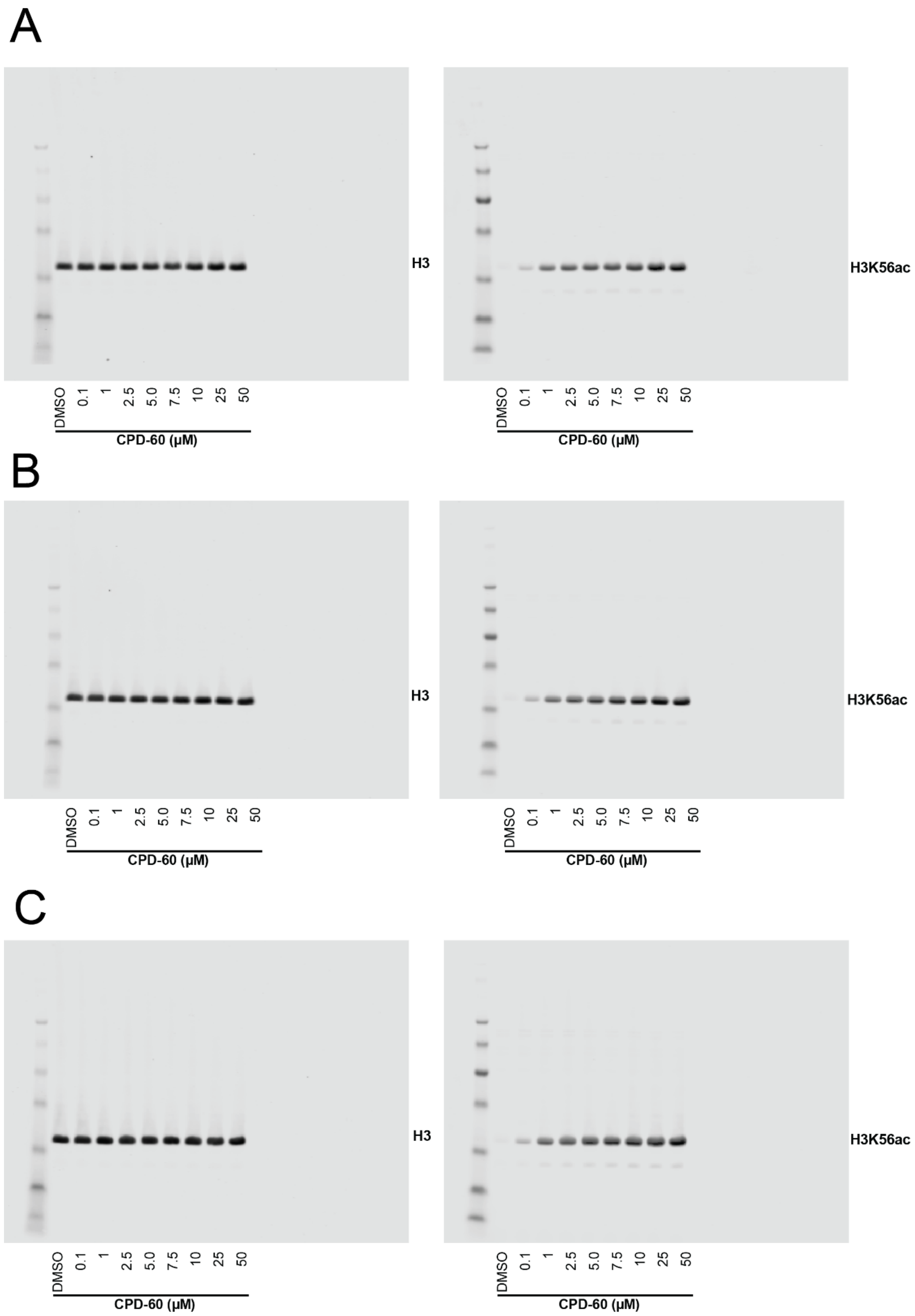

Figure S21 - (A) Unprocessed immunoblots of the H3K56Ac and H3 levels in HCT116 cells following a 24 h treatment with increasing concentrations of CDP-60; (A) Biological replicate 1 (B) Biological replicate 2 (C) Biological replicate 3.

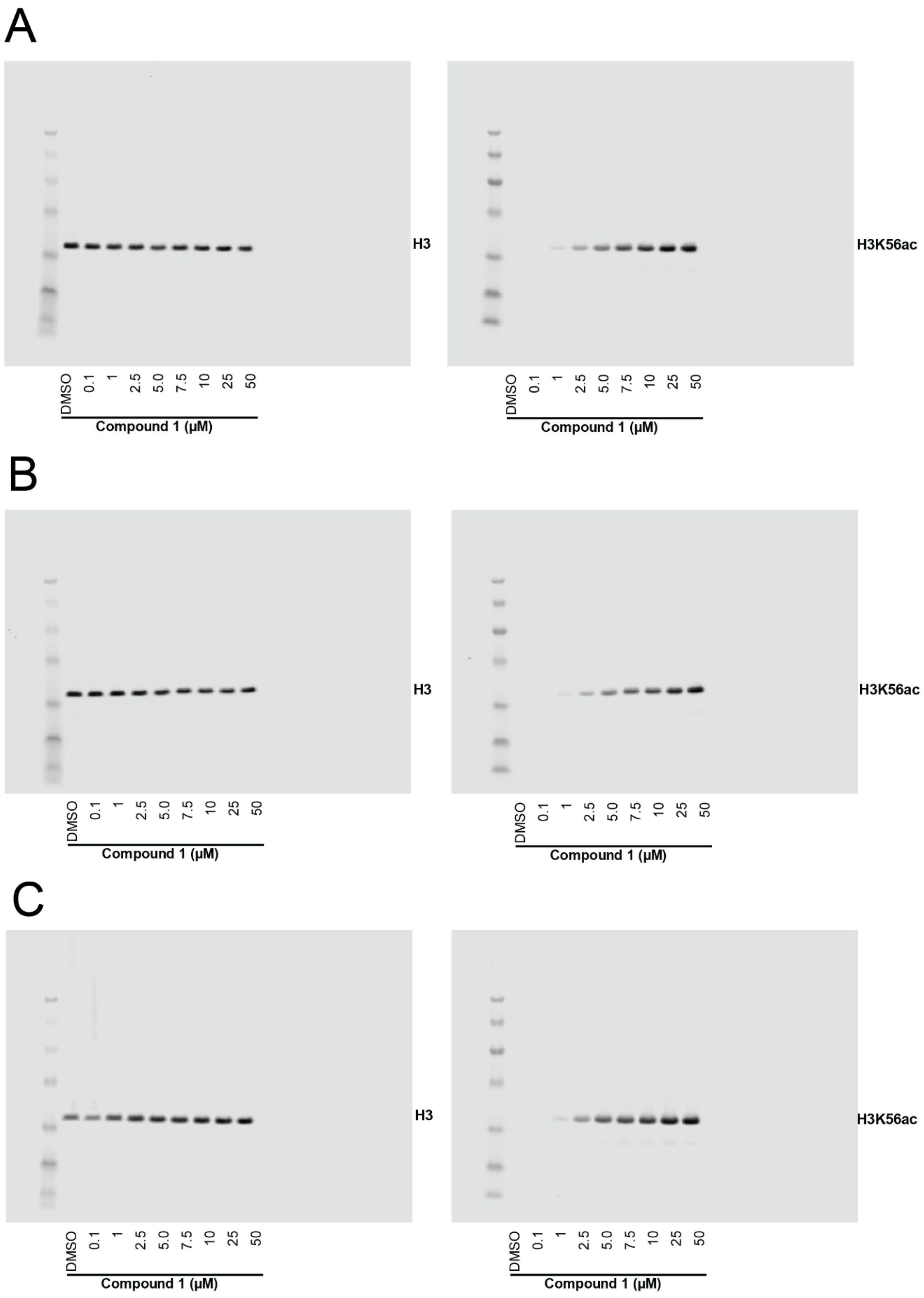

Figure S22 - (A) Unprocessed immunoblots of the H3K56Ac and H3 levels in HCT116 cells following a 24 h treatment with increasing concentrations of Compound 1; (A) Biological replicate 1 (B) Biological replicate 2 (C) Biological replicate 3.

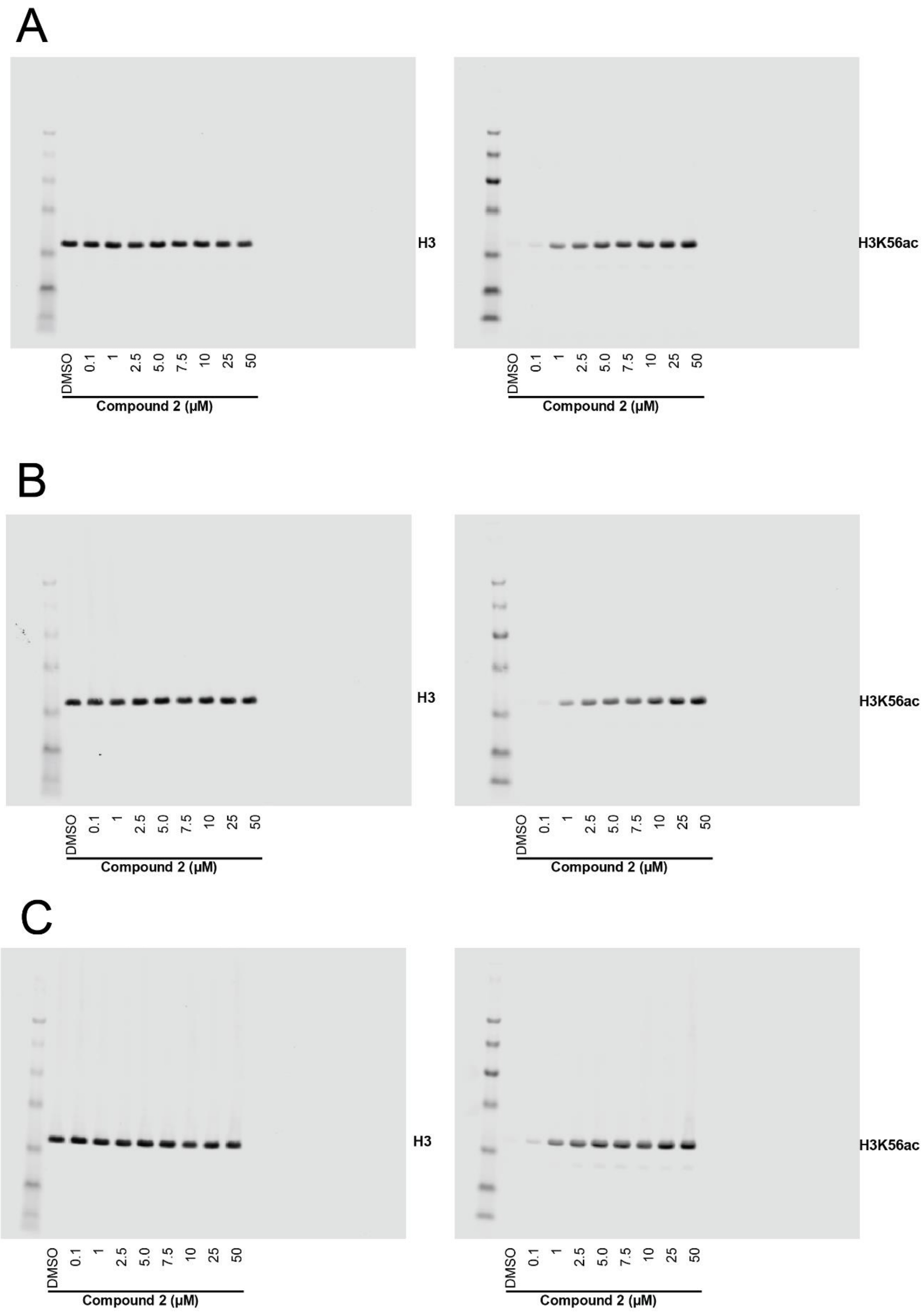

Figure S23 - (A) Unprocessed immunoblots of the H3K56Ac and H3 levels in HCT116 cells following a 24 h treatment with increasing concentrations of Compound 2; (A) Biological replicate 1 (B) Biological replicate 2 (C) Biological replicate 3.

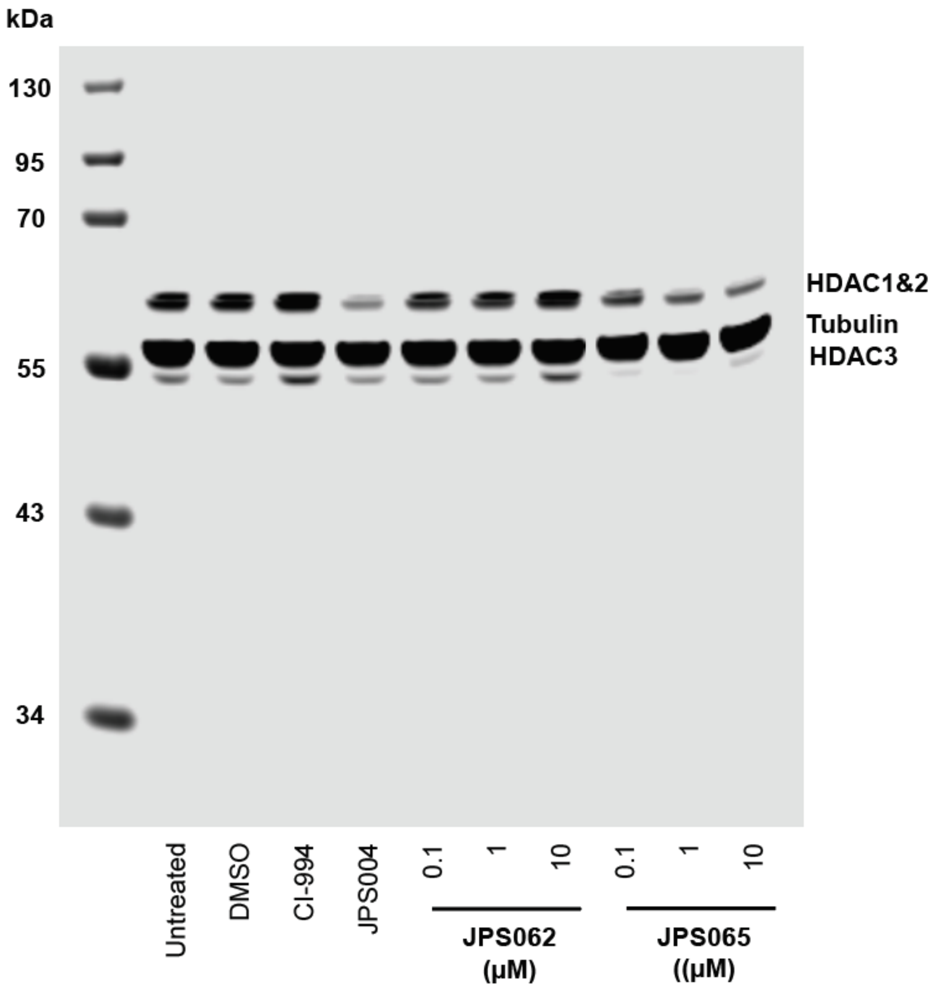

Figure S24 - Unprocessed immunoblots of the HDAC1, HDAC2 and HDAC3 levels in HCT116 cells following a 24 h treatment with increasing concentrations of JPS062 and JPS065. JPS004 was used as a positive control. Tubulin was used as a loading control.

Analytical data of HDAC inhibitors

SAHA

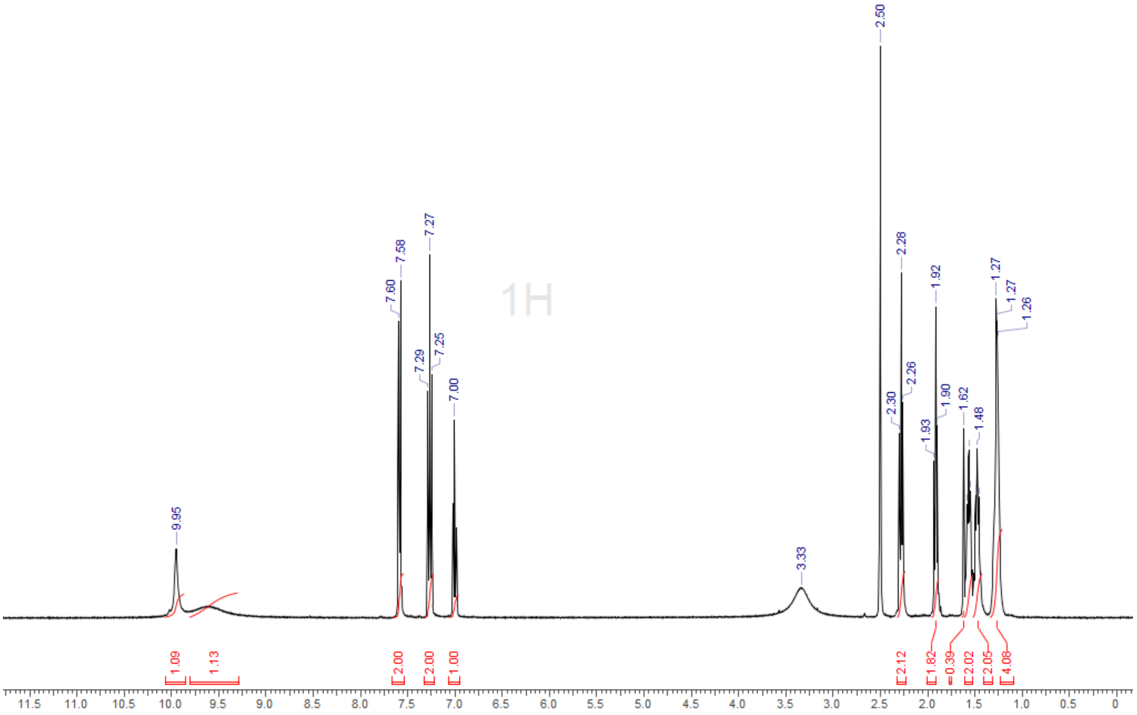

Figure S25 - ^{1}H NMR spectrum of SAHA.

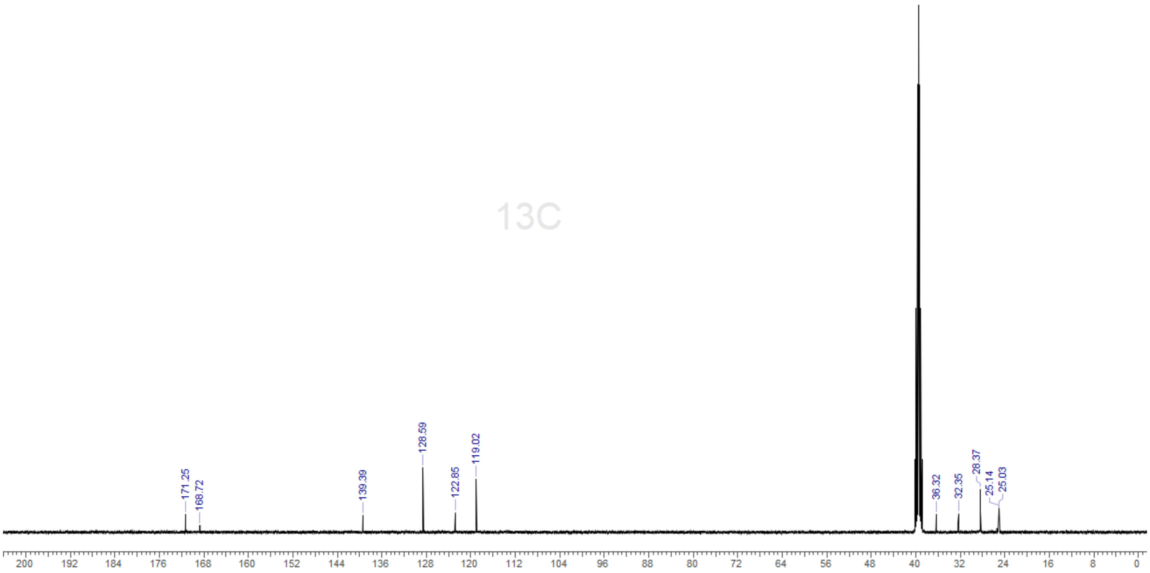

Figure S26 – ^{13}C NMR spectrum of SAHA.

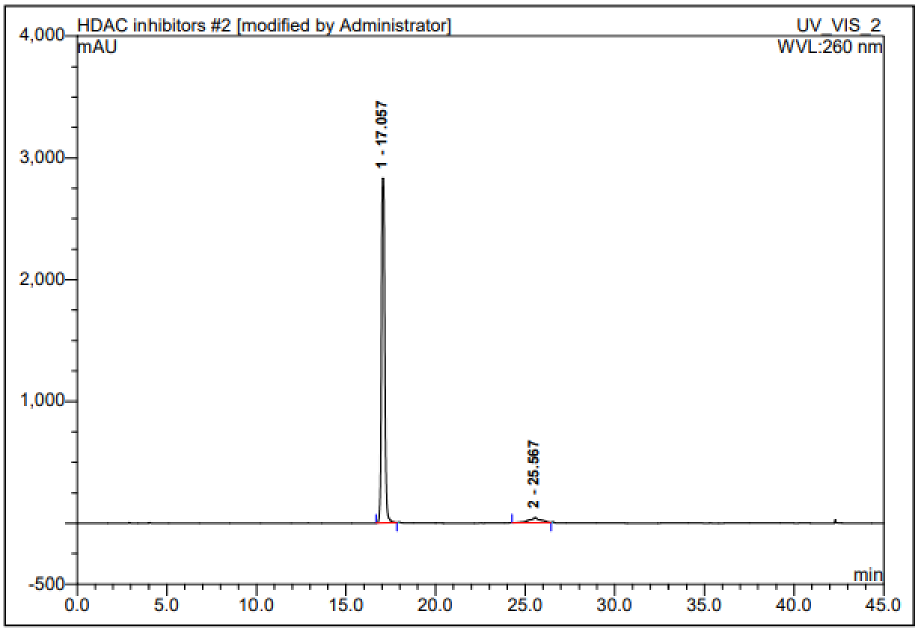

No.	Ret.Time min	Peak Name	Height mAU	Area mAU*min	Rel.Area %	Amount	Type
1	17.06	n.a.	2830.186	607.288	95.22	n.a.	BMB*
2	25.57	n.a.	41.549	30.471	4.78	n.a.	BMB*
Total:			2871.735	637.759	100.00	0.000	

Figure S27 – HLPC trace of SAHA.

Romidepsin

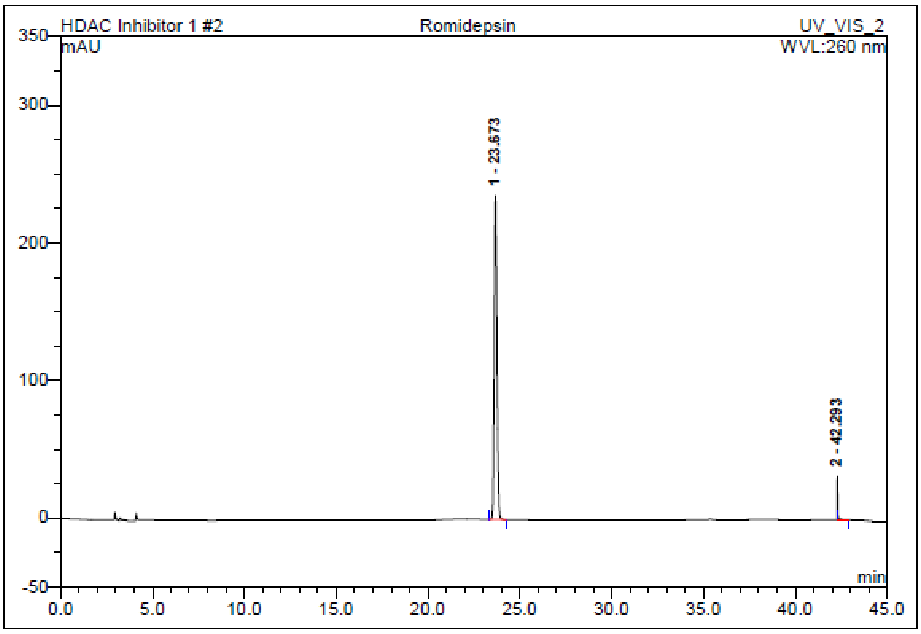

No.	Ret.Time min	Peak Name	Height mAU	Area mAU*min	Rel.Area %	Amount	Type
1	23.67	n.a.	235.116	41.483	97.37	n.a.	BMB
2	42.29	n.a.	31.449	1.120	2.63	n.a.	BMB
Total:			266.565	42.603	100.00	0.000	

Figure S28 – HPLC trace of Romidepsin.

MS-275

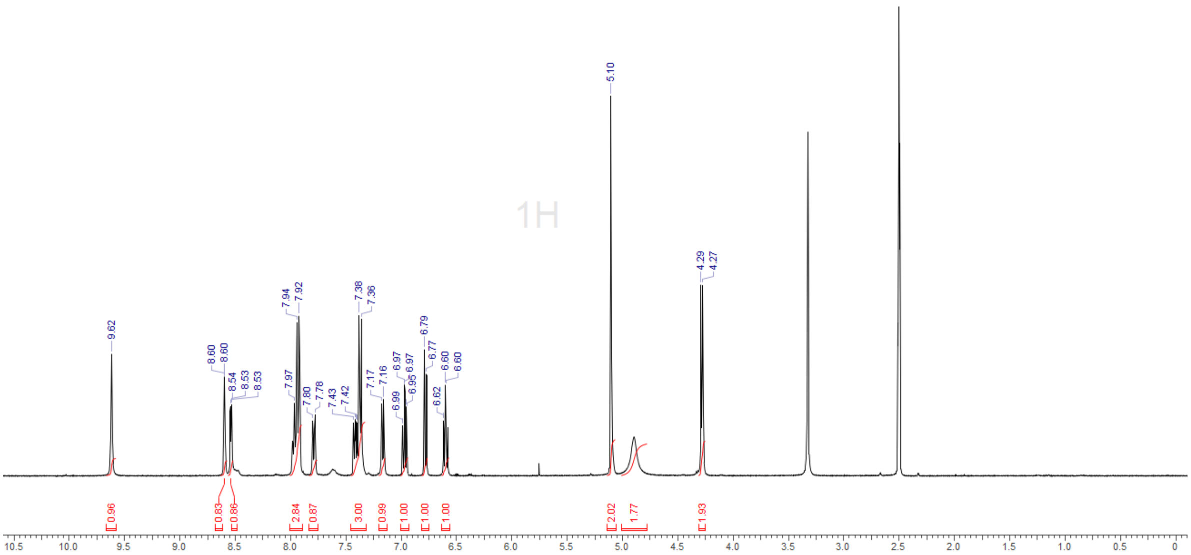

Figure S29 - ^{1}H NMR spectrum of MS-275.

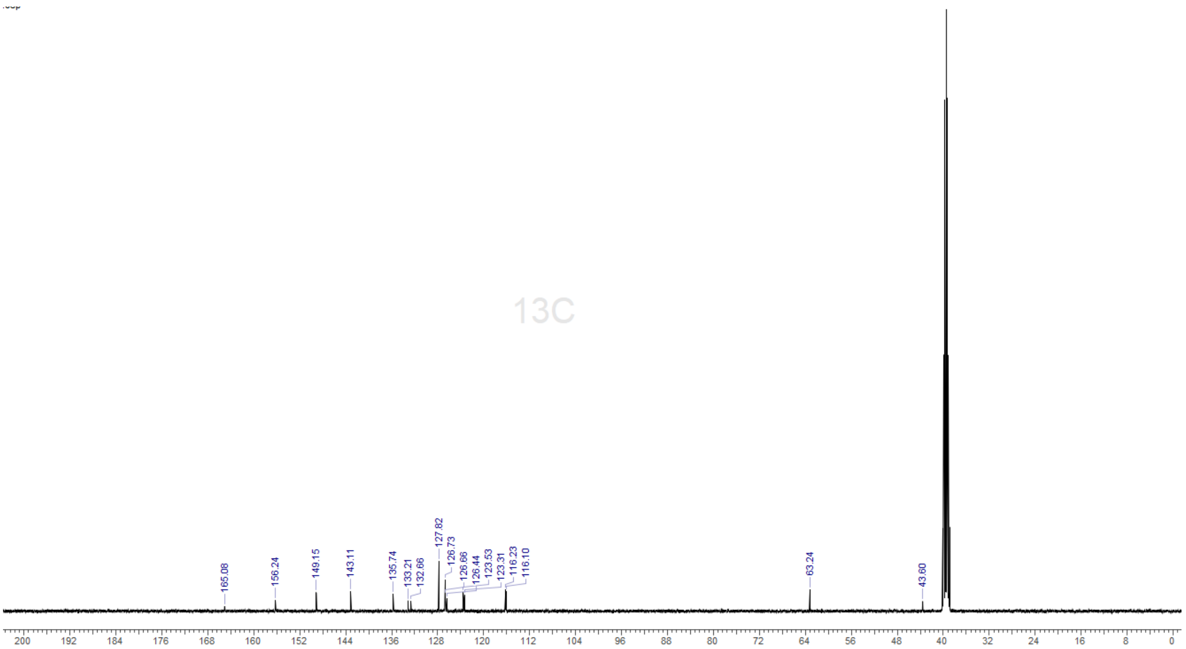

Figure S30 – ^{13}C NMR spectrum of MS-275.

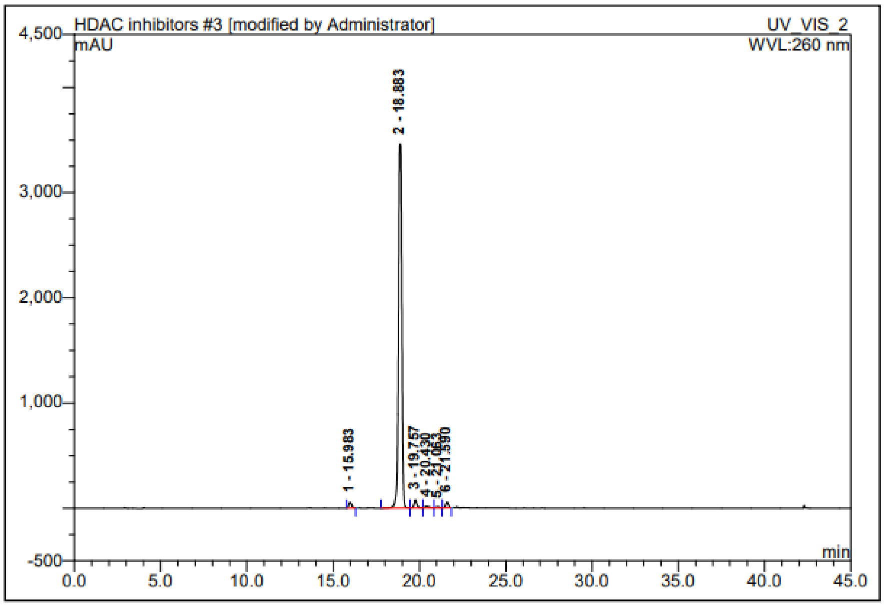

No.	Ret.Time min	Peak Name	Height mAU	Area mAU*min	Rel.Area %	Amount	Type
1	15.98	n.a.	55.070	10.326	1.29	n.a.	BMB*
2	18.88	n.a.	3461.361	759.090	95.04	n.a.	BMb
3	19.76	n.a.	78.504	13.456	1.68	n.a.	bMB
4	20.43	n.a.	13.866	3.845	0.48	n.a.	BMB
5	21.06	n.a.	9.498	2.129	0.27	n.a.	BMB
6	21.59	n.a.	56.272	9.884	1.24	n.a.	BMB
Total:			3674.571	798.730	100.00	0.000	

Figure S31 – HPLC trace of MS-275.

Compound 1

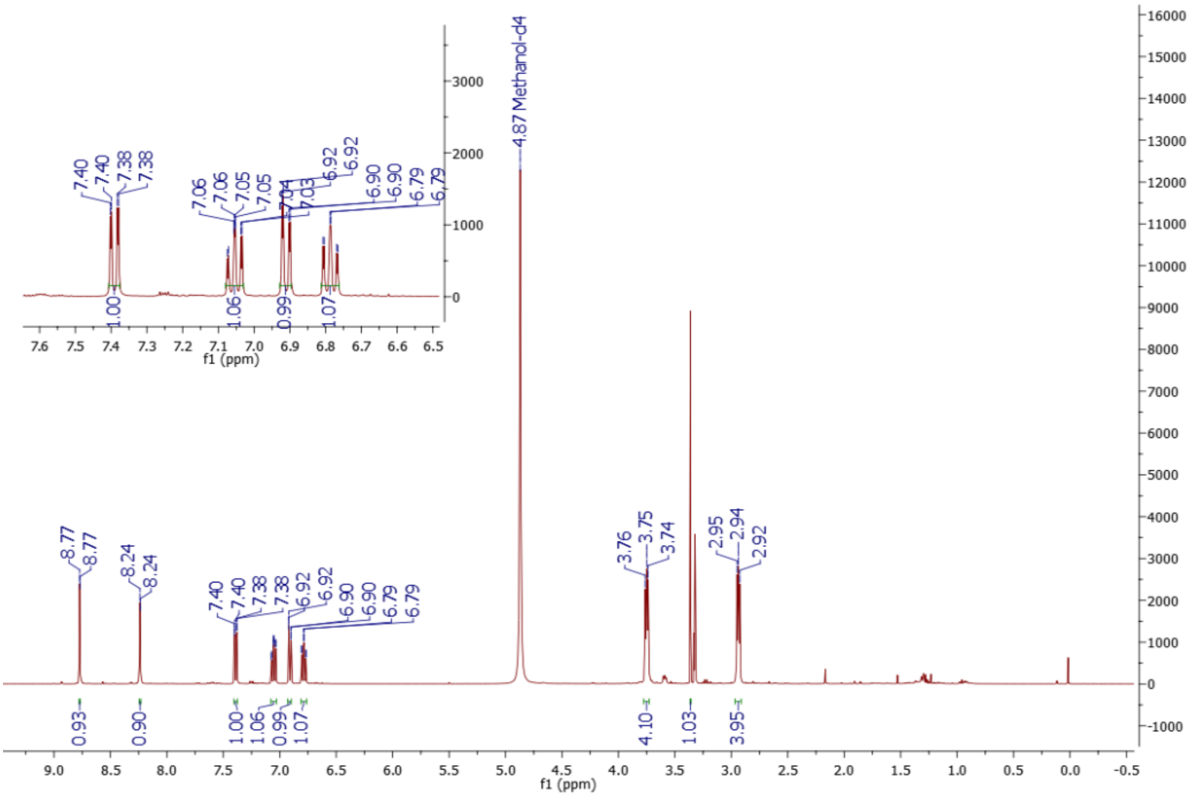

Figure S32 - ^{1}H NMR spectrum of Compound 1.

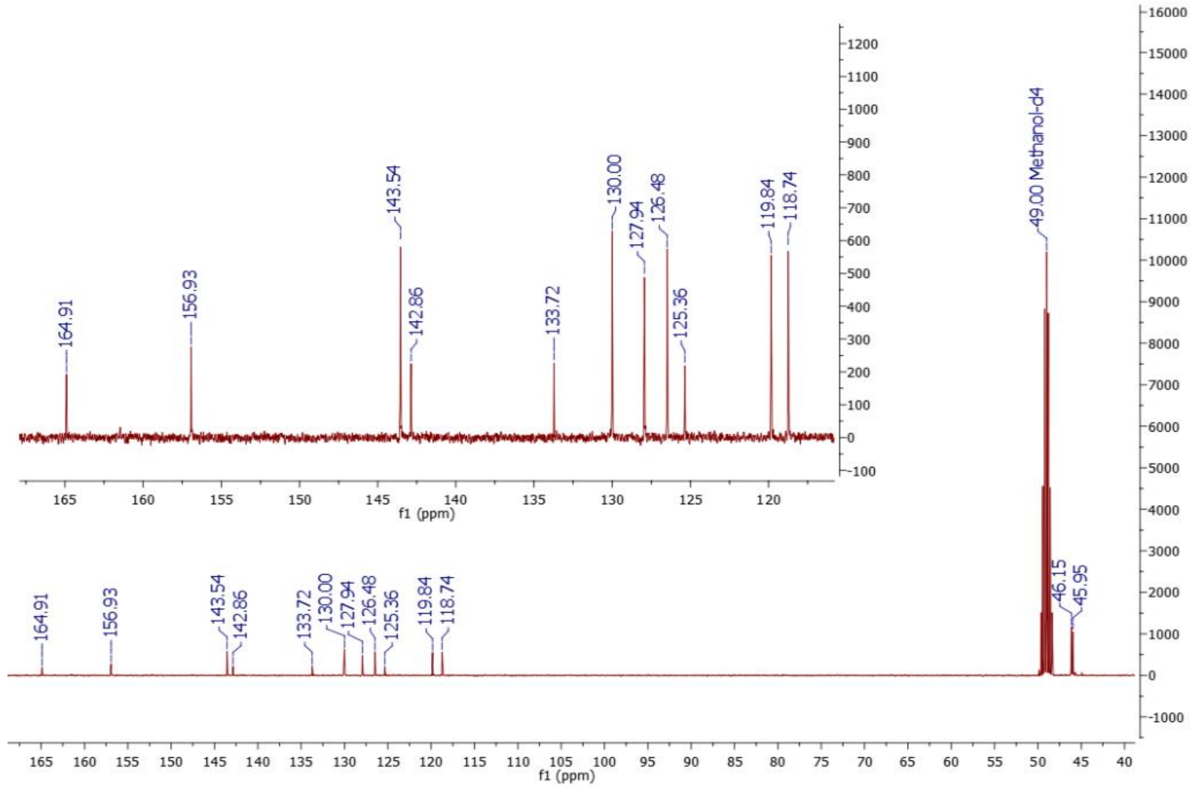

Figure S33 – ^{13}C NMR spectrum of Compound 1.

Compound 2

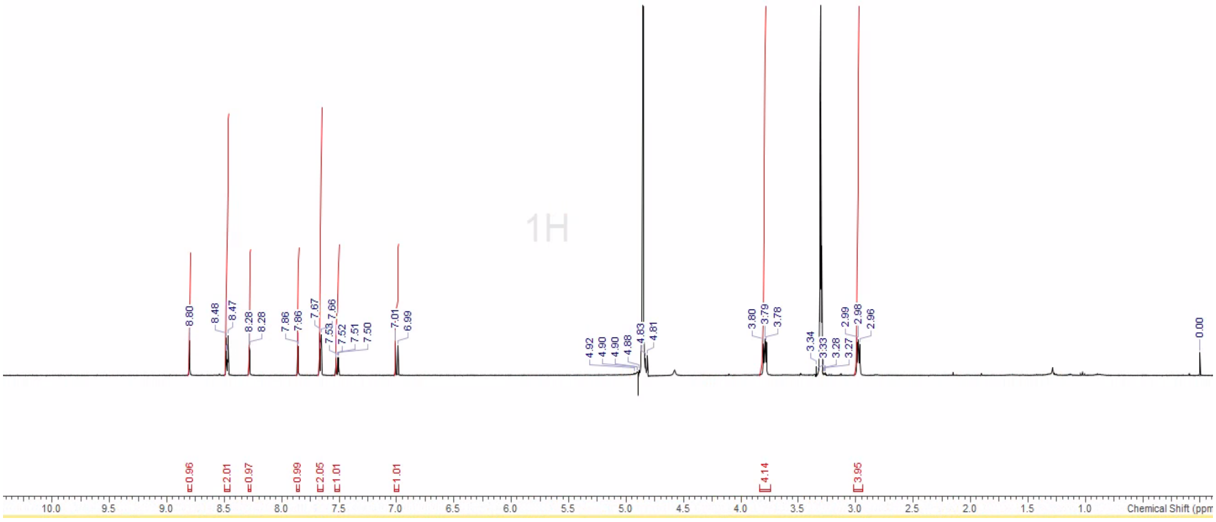

Figure S34 - ^{1}H NMR spectrum of Compound 2.

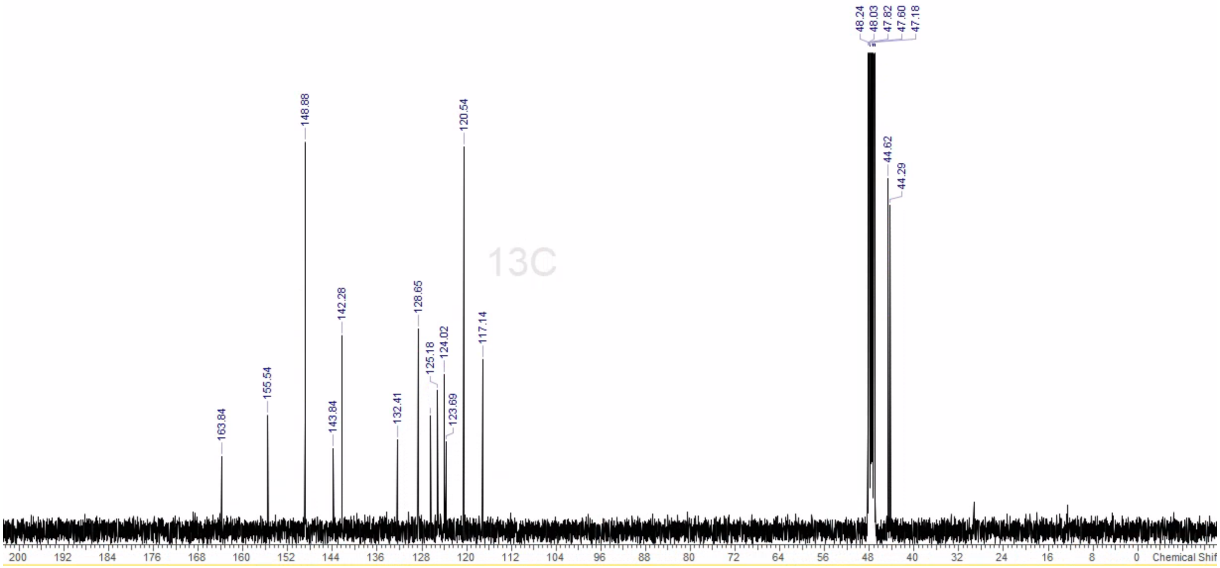

Figure S35 - ^{13}C NMR spectrum of Compound 2.

JPS062

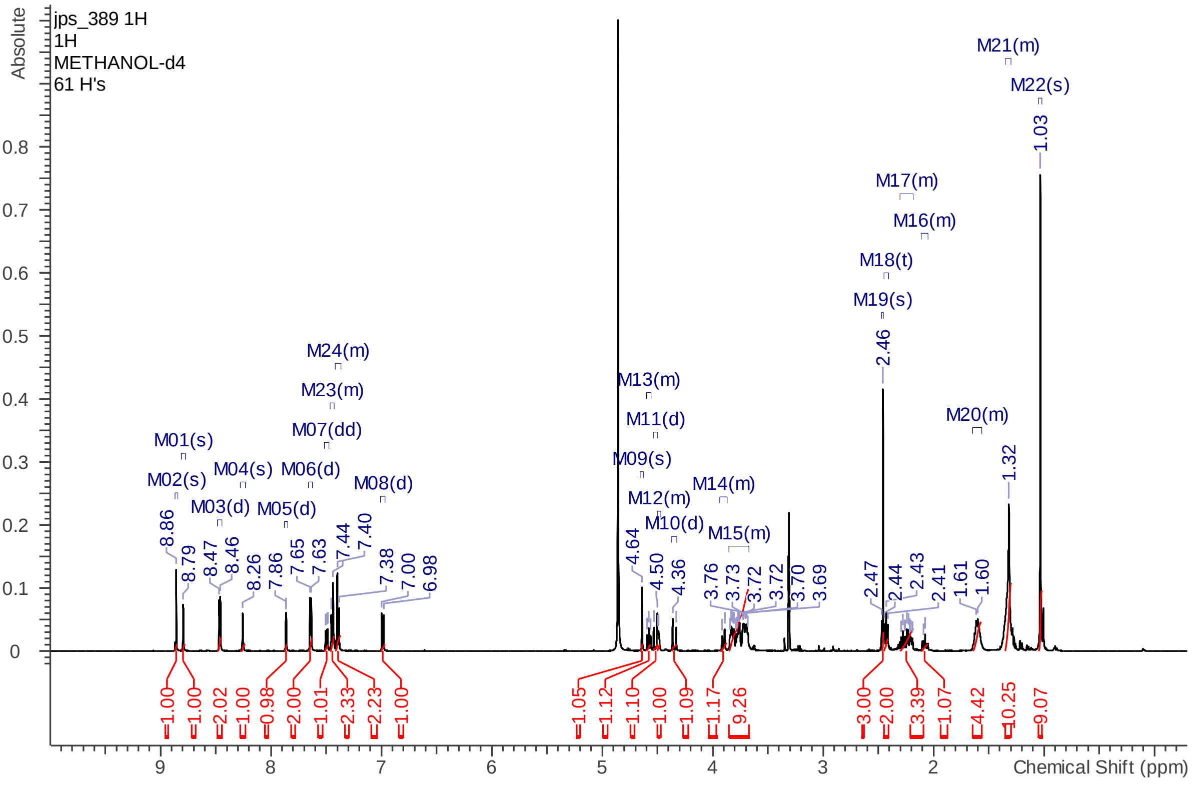

Figure S36 - ^{1}H NMR spectrum of JPS062.

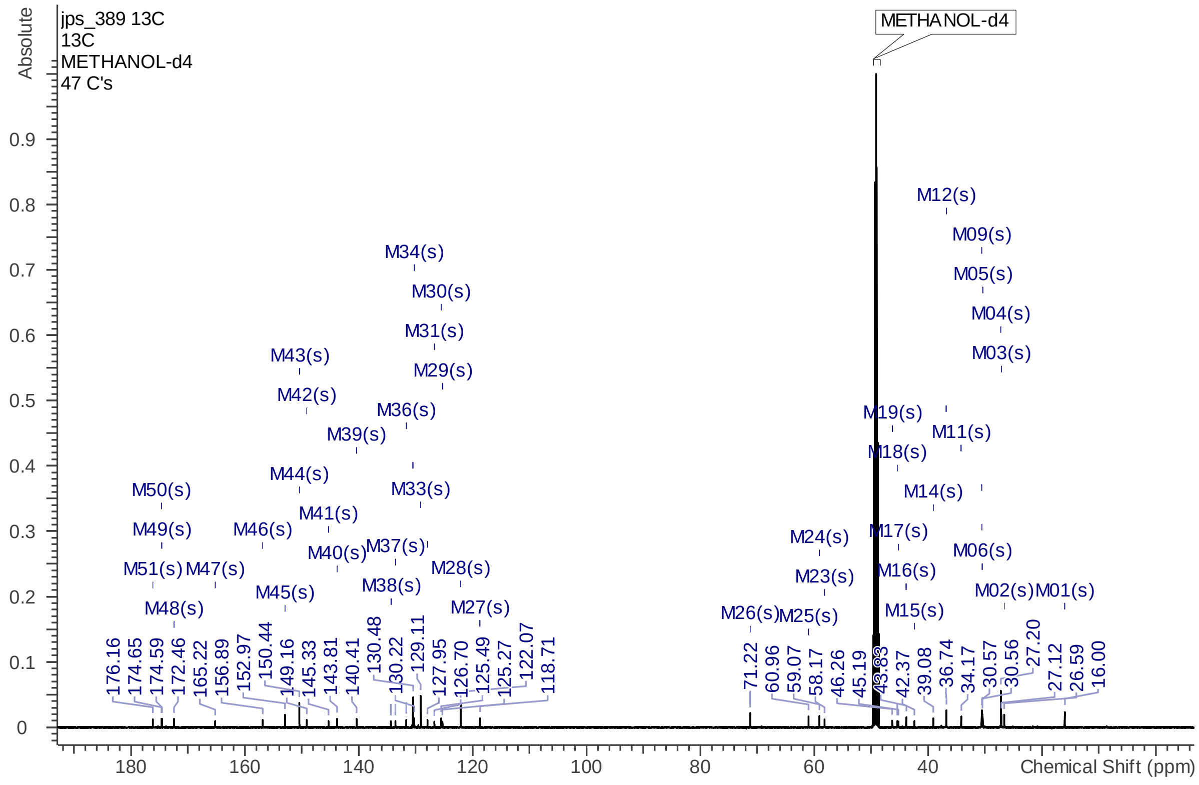

Figure S37 - ^{13}C NMR spectrum of JPS062.

JPS065

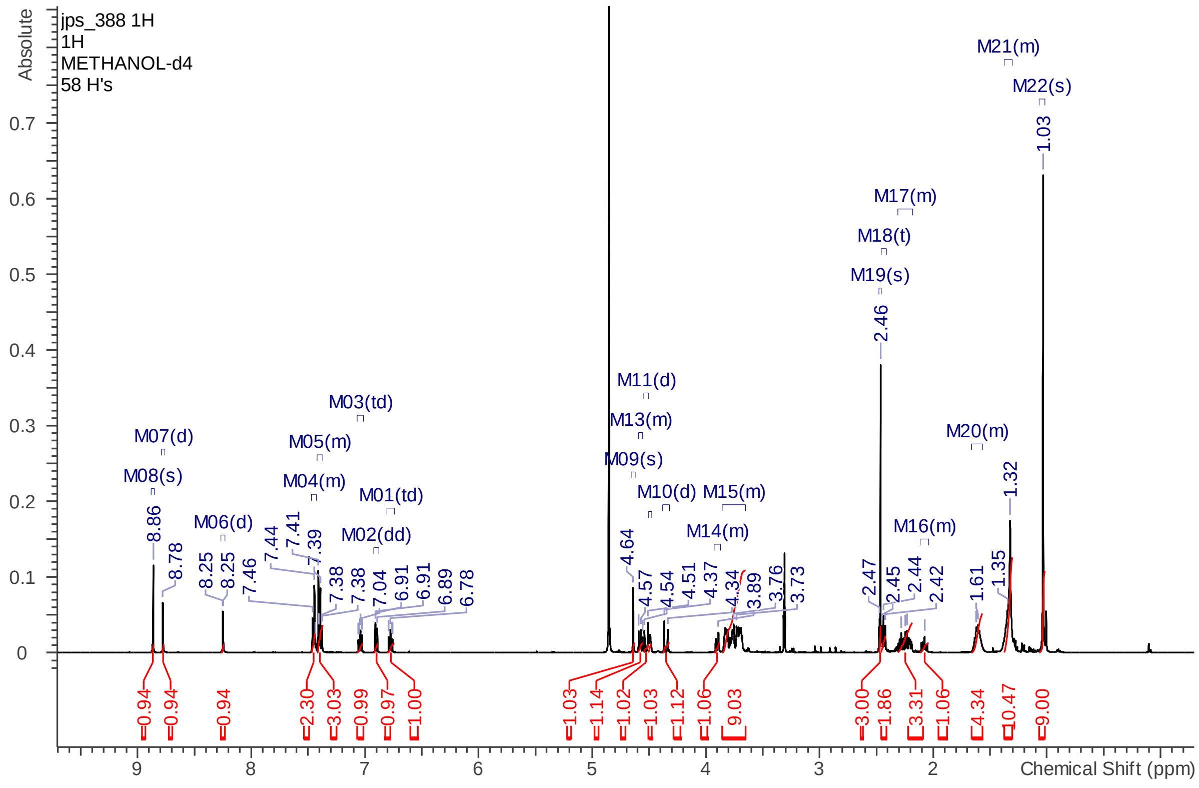

Figure S38 - ^{1}H NMR spectrum of JPS065.

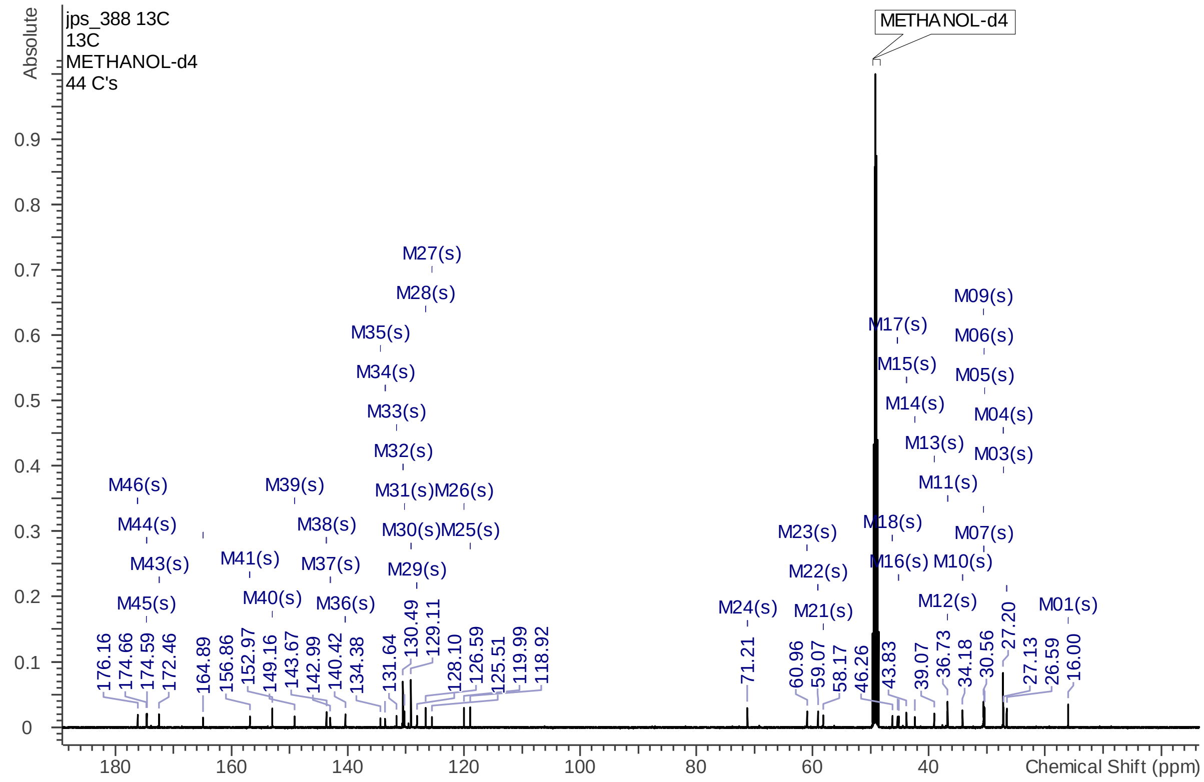

Figure S39 - ^{13}**C NMR spectrum of JPS065.**

References:

1 J. P. Smalley, G. E. Adams, C. J. Millard, Y. Song, J. K. S. Norris, J. W. R. Schwabe, S. M. Cowley and J. T. Hodgkinson, *Chem. Commun.*, 2020, **56**, 4476–4479.

2 A. R. Pavan, J. P. Smalley, U. Patel, W. A. Pytel, J. L. dos Santos, S. M. Cowley, J. W. R. Schwabe and J. T. Hodgkinson, *Chem. Commun.*, 2024, **60**, 13879–13882.

3 N. Portolano, P. J. Watson, L. Fairall, C. J. Millard, C. P. Milano, Y. Song, S. M. Cowley and J. W. R. Schwabe, *J Vis Exp*, 2014, 51897.
